# Employing Information Theoretic Measures and Mutagenesis to Identify Residues Critical for Drug-Proton Antiport Function in Mdr1p of *Candida albicans*

**DOI:** 10.1371/journal.pone.0011041

**Published:** 2010-06-10

**Authors:** Khyati Kapoor, Mohd Rehan, Andrew M. Lynn, Rajendra Prasad

**Affiliations:** 1 School of Life Sciences, Jawaharlal Nehru University, New Delhi, India; 2 School of Information Technology, Jawaharlal Nehru University, New Delhi, India; University of Minnesota, United States of America

## Abstract

By employing information theoretic measures, this study presents a structure and functional analysis of a multidrug-proton antiporter Mdr1p of *Candida albicans*. Since CaMdr1p belongs to drug-proton antiporter (DHA1) family of **M**ajor **F**acilitator **S**uperfamily (MFS) of transporters, we contrasted DHA1 (antiporters) with Sugar Porter family (symporters). Cumulative Relative Entropy (CRE) calculated for these two sets of alignments enabled us to selectively identify conserved residues of not only CaMdr1p but for the entire DHA1 family. Based on CRE, the highest scoring thirty positions were selected and predicted to impart functional specificity to CaMdr1p as well as to other drug-proton antiporters. Nineteen positions wherein the CaMdr1p residue matched with the most frequent amino acid at a particular alignment position of DHA1 members were subjected to site-directed mutagenesis and were replaced with either alanine or leucine. All these mutant variants, except one, displayed either complete or selective sensitivity to the tested drugs. The enhanced susceptibility of these mutant variants was corroborated with the simultaneously abrogated efflux of substrates. Taken together, based on scaled CRE between two MFS sub-families, we could accurately predict the functionally relevant residues of CaMdr1p. An extrapolation of these predictions to the entire DHA1 family members as validated from previously published data shows that these residues are functionally critical in other members of the DHA1 family also.

## Introduction

The azoles are a major class of drugs used to treat many fungal infections, and although they have good pharmacokinetic properties and are well tolerated, several fungi including pathogenic *Candida albicans* show innate or acquired azole resistance. A significant mechanism of azole resistance includes an over-expression of membrane proteins that actively efflux the incoming drugs [1]. Among the 28 putative **A**TP-**B**inding **C**assette (ABC) transporter genes and 95 putative Major Facilitator Superfamily (MFS) transporter genes identified in the *C. albicans* genome, only CaCdr1p and CaCdr2p among the ABC transporters and CaMdr1p among the MFS transporters are multidrug transporters involved in clinically encountered azole resistance of this fungal pathogen. Thus, azole resistant clinical isolates mostly show an increased expression of the plasma membrane efflux pumps encoding genes, viz. *CaCDR1*, *CaCDR2 and CaMDR1* [2], [3].

Studies in the major ABC transporters of *C. albicans* such as Cdr1p, Cdr2p have revealed that these transporters harbor multiple drug binding sites which are scattered within TMDs [4]. Notwithstanding similar topology and promiscuity towards substrates specificity, these ABC multidrug transporters of *Candida* also display selectivity to the range of substrates they export [5]. In comparison to ABC transporters, the structural and functional aspects of MFS multidrug transporters are only beginning to emerge. MFS is one of the largest families of transporters and includes members that function as uniporters, symporters or antiporters. Structural studies of MFS transporters revealed that the members of this superfamily share structural homology but have relatively weak sequence similarities. MFS transporters are an important superfamily of membrane proteins which import or export diverse substrates and catalyze different modes of transport using unique combinations of functional residues [6]. The structural studies conducted so far suggest the possibility that the fold of these transporters constitutes a scaffold for all MFS members with 12 helices. Although the fold is conserved, the specific function is obtained by varying sets of amino acids at the substrate binding and translocation domains. This is quite evident from the structural differences between Lactose Permease (LacY) of *E.coli* (symporter) and Glycerol-3-Phosphate (GlpT) of *E.coli* (antiporter) [7]. Very recently, we identified residues important for the entire MFS family irrespective of its mechanism of transport. However, our method did not identify subfamily function-specific residues [8].

Predicting functionally important residues from a Multiple Sequence Alignment (MSA) by using the criteria of amino acid conservation and then employing site-directed mutagenesis for validation has been a common approach. Multiple methods for scoring the amino acid conservation are being used frequently [9]. However, this process has serious limitations, particularly since the criterion of conservation across the sequence alignment, does not necessarily predict the functional importance of a residue for a given protein. A residue shown to be conserved across MSA can be important for the entire class and can have a role in maintaining the fold of the protein rather than playing a more specific role in its function [8]. Moreover, if a dataset has a significant number of recently evolved sequences, the conservation score of the alignment columns picked up as conserved might be an erroneous representation of the data set.

The neutral theory of molecular evolution states that once a protein has evolved to a useful level of functionality the majority of mutations are selectively neutral at the molecular level and do not affect the function and folding of the protein whereas those mutations which are deleterious (loss of function or misfolding) provide selection pressure for residue conservation [10]. Thus, the residue conservation in a multiple alignment of a protein and its homologues indicates the importance of the residue for maintaining the structure and function of the protein. Claude Shannon founded information theory in 1940s and this theory has long been known to be closely related to thermodynamics and physics [11]. In 1991, Sander and Schneider used Shannon entropy derived scores for positional conservation in alignment of proteins [12].

The commonly used and well known information theoretic scores are entropy (H) and relative entropy (RE). The entropy score for each aligned column in a MSA can be expressed as:
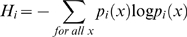
where P(x_i_) is the probability of finding amino acid type **x** at aligned column **i**.

The entropy value is maximum for an alignment column having randomly distributed amino acids while it is zero for a completely conserved column.

Relative entropy is the measure of divergence between the two probability distributions of residues. This calculates conservation of aligned column over background probability distribution.
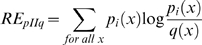
where q(x) is the background probability of amino acid type **x**.

Background probability means the probability of occurrence of a particular amino acid in nature (calculated from protein sequence database, Swissprot) [13]. Often, the mutational changes at particular conservation site after the gene duplication leads to functional divergence [14]. The residues of a protein at these sites are called specificity determining residues (SDR) which when mutated lead to changes in the protein's function. The information theoretical methods have been increasingly applied in bioinformatics to identify such SDRs [11], [15], [16]. Given an alignment, and a set of proteins grouped into sub-families, according to some definition of function, such as catalytic activity, the method identifies positions that are indicative of these functional differences between the mentioned sub-families [9]. In view of this, we used information theoretic measure, scaled cumulative RE (CRE_S_) in preference to traditional conservation measures [16], to predict residues important for DHA1 family of which CaMdr1p is a member, and extrapolated it to other members of its family. In this manuscript, we contrast DHA1; a family of drug-proton antiporters, with Sugar Porter (SP); a family of symporters to predict the residues important for a drug-proton antiporter exclusively, on the basis of CRE_S_. We selected thirty residues with highest CRE_S_ for analysis. Selected residues were subjected to site-directed mutagenesis and replaced with alanines or leucines. Notably, the mutant variants of these residues showed abrogation of resistance to either all or to selected drugs. The enhanced susceptibility of these mutant variants to the drugs was corroborated with abrogated efflux of the substrates. From previously published data, we validated our predictions by showing that the equipositional residues in other members of the DHA1 family are also functionally critical. Our study shows that the use of CRE_S_ provides an impartial score to select differentially conserved residues, which can be evaluated for their functional significance.

## Results

### CRE_S_ scores predict functionally important residues of CaMdr1p

We attempted to rationalize the site-directed mutational strategy in order to predict functionally critical residues of CaMdr1p. As CaMdr1p belongs to DHA1 family; a family of drug-proton antiporters, we contrasted its sequences with another family of MFS transporters having totally different function. For this sort of comparison, SP family; a family of symporters was chosen as it completely differs from DHA1 in terms of mechanism of transport as well as its substrate specificity. Upon alignment of sequences from these two families, it was hypothesized that those residues which are exclusively conserved or are differentially conserved in DHA1 will be critical for DHA1-specific function. We speculated that such a contrast will predict residues exclusively important for DHA1 family. The dataset included 37 of DHA1 and 44 of SP sequences with no redundancy. Other such contrasting families of MFS have very few members and were not included since the use of smaller dataset is prone to statistical errors.

**Figure 1:**
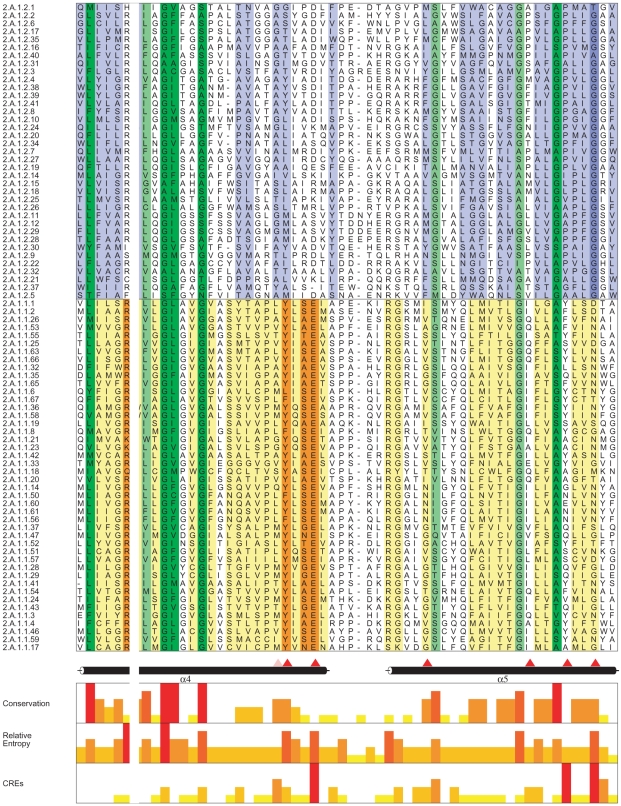
Representative alignment of MFS sequences. Figure showing a representative portion of the Multiple Sequence Alignment of the MFS sequences and is generated using Alscript [31]. The top 37 sequences which are boxed belong to the DHA1 family and the next 44 belong to the SP family. The part of the MSA spanning helix 4 and 5 is highlighted in the figure. Alignment columns are coloured in a gradient based on the degree of conservation. TCDB ID of CaMdr1p Sequence is 2.A.1.2.6 and is third from the top. The complete MSA is shown in Supplementary Data DS1. Green columns denote family-wide conservation, while conserved columns in DHA1 and SP family are coloured in blue and yellow respectively. A comparison of conservation, RE and CRE_S_ scores are shown as histograms [28]. CRE_S_ identifies columns which are differentially conserved in the DHA1 family. The phenotypes of the mutated positions in CaMdr1p are indicated by triangles, red being sensitive and pink being differential.

**Figure 2:**
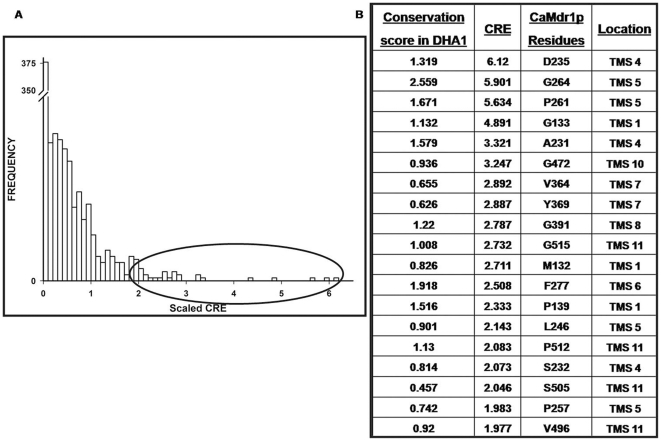
Distribution curve of CRE_S_ values from the complete MSA. A: Histogram of the CRE_S_ scores for all positions of an MSA of DHA1 and SP family. The 30 highest scoring CRE_S_ positions depicted in the marked region were selected for further analysis. B: The table shows 19 out of the 30 highest scoring CRE_S_ alignment positions wherein the CaMdr1p residue matched with the most frequent amino acid at that position in the MSA. Their predicted location with respect to CaMdr1p is also displayed in the next column.

We then employed a well-known information theoretic measure, CRE, with this comprehensive non-redundant dataset. The complete alignment in clustalw format is shown in Supplementary Data S1. The RE within a family (conservation across a subfamily) and CRE_S_ scores across all columns of this profile MSA were calculated Supplementary Data S2. Figure 1 shows a representative part of this profile alignment with Cumulative Relative Entropy (CRE_S_), Relative Entropy (RE) and conservation scores for each alignment position. We predicted that for a residue to be functionally critical, it should not only have a high CRE score but should also be highly conserved in the DHA1 family. Thus, a criterion of selection which took both the conservation score and the CRE score in consideration was required. For this, a scaled CRE score (CRE_S_), a product of conservation across only DHA1 (measured using RE_NULL_), and CRE between the two families were calculated. A residue position with high CRE_S_ was predicted to be either differentially conserved across the two families or exclusively conserved in the DHA1 family vis-à-vis the SP family and thus was predicted to impart functional specificity to DHA1. The distribution curve generated on the basis of CRE_S_ scores of the entire MSA is shown in Figure 2A. The thirty residues with highest CRE_S_ were shortlisted for further studies. The range of selection spanned from a scaled score of 6.12-1.96 and is highlighted in Figure 2A.

### Site-specific mutagenesis confirms CRE_S_ based selection of functionally critical residues

Nineteen out of the thirty highest CRE_S_ alignment positions had the most frequent amino acid matching with the CaMdr1p residue. These are enlisted in Figure 2B and include D235, G264, P261, G133, A231, G472, V364, Y369, G391, G515, M132, F277, P139, L246, P512, S232, S505, P257 and V496. These residues which were predicted to be functionally relevant were subjected to site-directed mutagenesis and were replaced with alanine. In the cases where glycines were present, they were replaced with leucine while the existing alanines were replaced with glycines. For functional analysis of the mutant variants, we used a heterologous hyper-expression system, where GFP-tagged CaMdr1p (CaMDR1-GFP) was stably over-expressed from a genomic *PDR5* locus in AD1-8u^-^, a *S. cerevisiae* mutant. The host AD1-8u^-^ developed by Goffeau's group, was derived from a *Pdr1-3* mutant strain with a gain-of-function mutation in the transcription factor Pdr1p, resulting in constitutive hyper-induction of the *PDR5* promoter. A single-copy integration of each transformant at the *PDR5* locus was confirmed by Southern hybridization (data not shown). Two positive clones of each mutant were selected to rule out clonal variations.

**Figure 3:**
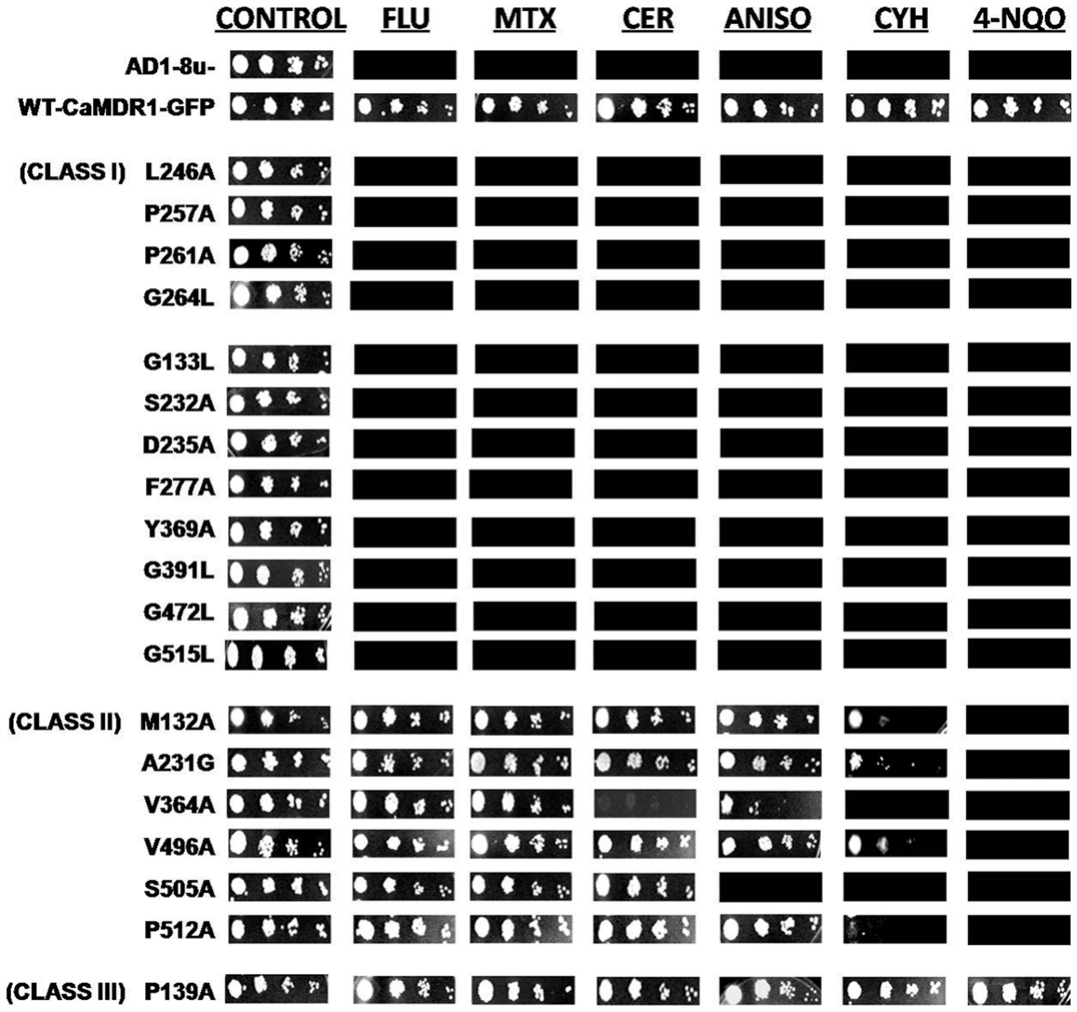
Drug susceptibility assay of mutant variants of CaMDR1-GFP. A: Drug resistance profile of wild type and mutant CaMDR1-GFP yeast strains as determined by spot assay. 5 µl of five-fold serial dilutions, namely 1 (1∶5), 2 (1∶25), 3 (1∶125) and 4 (1∶625), of each strain was spotted on to YEPD plates as described previously in the absence (control) and presence of the following drugs: FLU (0.20 µg/ml), MTX (65 µg/ml), CER (3 µg/ml), ANISO (3 µg/ml), CYH (0.20 µg/ml) and 4-NQO (0.20 µg/ml). Growth differences were recorded following incubation of the plates for 48 hrs at 30°C. Growth was not affected by the presence of the solvents used for the drugs (data not shown).

The mutant variants of CaMdr1p were analyzed for their drug susceptibility by employing microtiter plate and spot assays. All the high CRE_S_ residues except P139A showed decreased resistance to tested drugs. Though all the cells expressing mutant variant CaMdr1p, grew poorly on solid as well as in liquid media, a closer look revealed the variation in specificity of these residues to drugs and their magnitude of resistance. On the basis of selective sensitivity to various drugs, these mutant variants could be grouped into three classes. Class I included the mutant variants such as D235A, G264L, G133L, G472L, Y369A, G391L, G515L, F277A and S232A which displayed sensitivity towards all the drugs. Notably, P261A, L246A and P257A belong to antiporter motif which also bunched within this class. The residues which showed selective increase in sensitivity towards the considered spectrum of drugs were grouped in Class II. The six mutant variants A231G, M132A, P512A, V496A, S505A and V364A of this class showed selective decrease in resistance at differential levels for various drugs. For example, mutant variants A231G, M132A, P512A and V496A continued to display resistance to FLU, MTX, ANISO, CER but exhibited sensitivity towards CYH and 4-NQO. S505A was susceptible to ANISO along with CYH and 4-NQO. V364A, on the other hand was different from these as it was resistant only to FLU and MTX while showed sensitivity towards, CER, ANISO, CYH and 4-NQO. The mutant variant P139A which behaved like WT-CaMdr1p-GFP was placed under Class III (Figure 3). The spot assay results were confirmed by microtiter assays as well. The MIC_80_ values of all these mutant variants are listed in Table 1 and are the index of susceptibility in the liquid medium.

**Table 1:** MIC_80_ values of the mutant variants of CaMdr1p.

	MIC_80_ (µg/ml)					
STRAIN	FLU	MTX	ANISO	CER	CYH	4-NQO
AD1-8u-	0.5	16	0.5	0.5	0.015	0.03
WT-CaMDR1-GFP	16	128	32	8	0.5	1.0
**CLASS I**						
**Residues in Antiporter motif**						
P261A	0.5	16	0.5	0.5	0.015	0.03
L246A	0.5	16	0.5	0.5	0.015	0.03
P257A	0.5	16	0.5	0.5	0.015	0.03
G264L	0.5	16	0.5	0.5	0.015	0.03
**Other Residues**						
D235A	0.5	16	0.5	0.5	0.015	0.03
G133L	0.5	16	0.5	0.5	0.015	0.03
G472L	0.5	16	0.5	0.5	0.015	0.03
Y369A	0.5	16	0.5	0.5	0.015	0.03
G391L	0.5	16	0.5	0.5	0.015	0.03
G515L	0.5	16	0.5	0.5	0.015	0.03
F277A	0.5	16	0.5	0.5	0.015	0.03
S232A	0.5	16	0.5	0.5	0.015	0.03
**CLASS II**						
A231G	8	64	8	4	0.125	0.06
M132A	4	64	32	4	0.125	0.03
P512A	8	64	4	4	0.125	0.06
V496A	8	64	16	2	0.125	0.06
S505A	8	64	0.5	4	0.03	0.06
V364A	4	32	2	1	0.03	0.03
**CLASS III**						
P139A	16	128	32	8	0.5	1.0

The residues at high CRE_S_ positions were tested for their drug susceptibility and their MIC_80_ (µg/ml) values are listed in comparison with the WT-CaMDR1-GFP and AD1-8u^-^ (Negative control). More than 2-well difference in Microtiter plates was considered as significant, which matched with the results reported on the solid medium in the spot assays [30].

### All the mutant variants of CaMdr1p are normally expressed and properly localized

**Figure 4:**
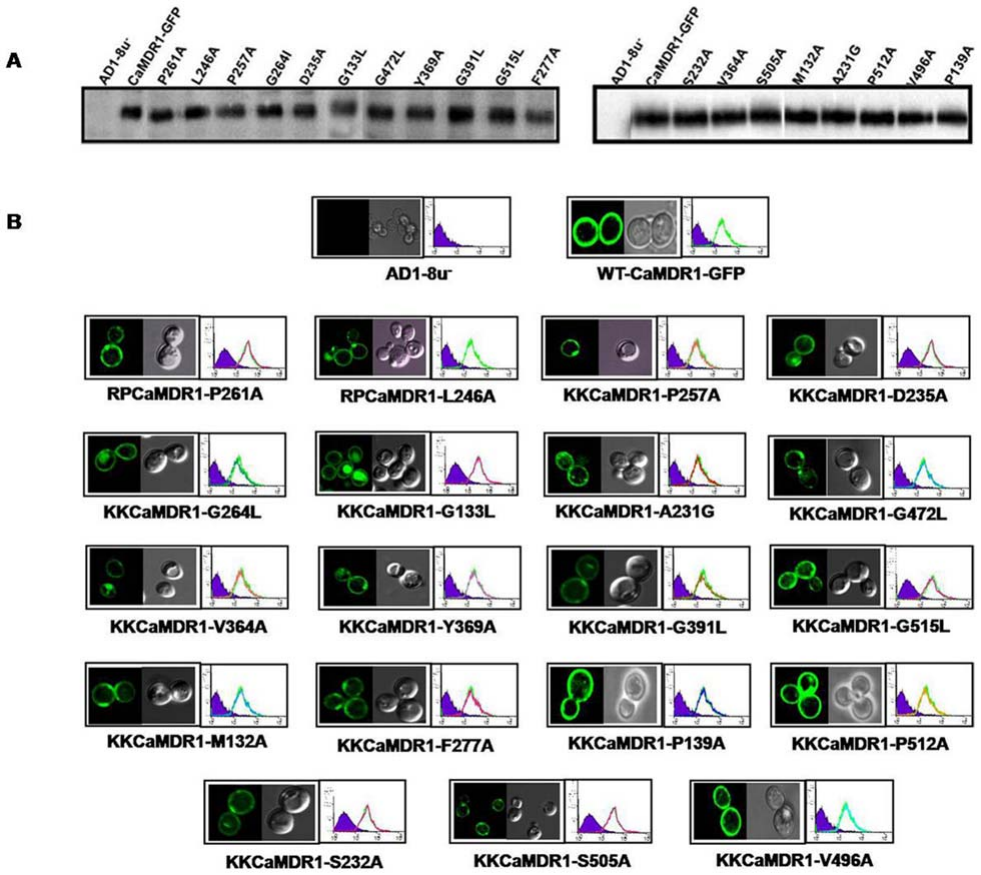
Expression profiles of CaMdr1p-GFP and its mutant variants. A: Western Blot analysis of the PM fraction of the mutant variants with anti-GFP antibody. B: Confocal and FACS analysis of the all the mutant variants to check their expression and localization in comparison with AD1-8u^-^ (negative control) and WT-CaMDR1-GFP (positive control).

To confirm that the change in susceptibility of mutant variants to various drugs was not due to their poor expression or surface localization, we compared the surface expression and localization of GFP-tagged version of CaMdr1p (CaMDR1-GFP) and its mutant variants. Notwithstanding the fact that all the mutant variants displayed decreased resistance to drugs, their expression levels matched with that of WT-CaMdr1p-GFP as confirmed by Western Blot Analysis (Figure 4A) while both FACS and confocal imaging confirmed their proper surface localization (rimmed appearance of GFP-tagged CaMdr1p) (Figure 4B).

### High CRE_S_ residues displayed abrogated efflux

**Figure 5:**
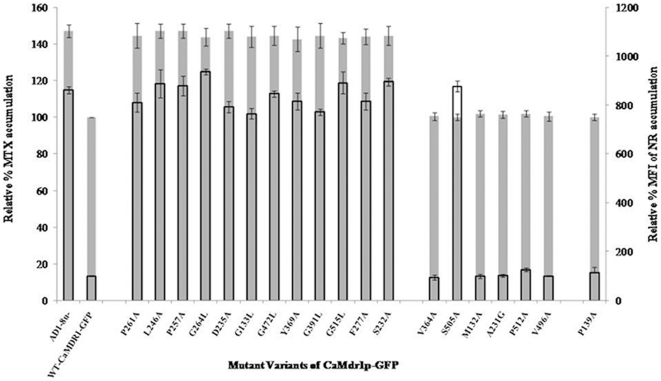
[^3^H] MTX and NR accumulation in the mutant variants of CaMdr1p-GFP. The graph shows accumulation levels of these substrates relative to WT-CaMDR1-GFP. The grey colored bars (no border) indicate levels of accumulation for [^3^H] MTX while empty bars (black border) indicate that for NR. Controls AD1-8u^-^ and CaMdr1p-GFP have also been included for comparison. The accumulation assays with [^3^H] FLU gave similar results as [^3^H] MTX for all the mutant variants (data not shown). The results are means ± standard deviations for three independent experiments.

To validate the drug susceptibility data of the mutant variants, we conducted efflux measurements by employing three substrates, fluorescent NR, and radiolabel [^3^H] FLU and [^3^H] MTX. All the three compounds have been shown to be the substrates of CaMdr1p [8], [17]. The intracellular concentration of these substrates was determined with respect to time. It was observed that there was minimum accumulation of substrates at 30 minutes. Hence a time point of 30 minutes was selected for checking intracellular accumulation of all these three substrates in the mutant variants and then compared with host AD1-8u^-^. Notably, the level of intracellular accumulation of [^3^H] FLU, [^3^H] MTX and NR is indicative of high (low accumulation) or low (high accumulation) efflux activity of the cell mediated by CaMdr1p. Site-directed mutant variants of these high CRE_S_ positions showed increased accumulation at 30 minutes as compared to that of WT-CaMdr1p and thus indicating a decrease in their ability to efflux NR, [^3^H] FLU and [^3^H] MTX. The increased accumulation of NR and [^3^H] MTX in mutant variants D235A, G264L, P261A, G133L, G472L, Y369A, G391L, G515L, F277A, L246A, S232A and P257A, was attributed to the loss of efflux activity upon mutation. All the other mutant variants which though exhibited different levels of sensitivity to various drugs i.e. V364A, M132A, A231G, P512A and V496A or which remained resistant to all drugs i.e. P139A, continued to efflux these three substrates as efficiently as WT-CaMdr1p. Exceptionally, S505A, which exhibited differential sensitivity towards the drugs also showed differential accumulation of these substrates. For example, S505A displaying decreased accumulation in case of [^3^H] FLU and [^3^H] MTX, showed no change in NR accumulation which was comparable to AD1-8u^-^ cells (Figure 5).

## Discussion

CaMdr1p is one of the major MDR transporter involved in frequently occurring azole resistance in *C. albicans*. The efflux pump proteins display promiscuity towards substrate specificity wherein a very large number of structurally diverse compounds can be extruded by the transporter. In-depth knowledge of protein structure and function is essential for any logical approach to block the activity of such protein in MDR isolates of *Candida*. This study represents an attempt in that direction wherein a rational approach is applied to predict functionally critical amino acids of this transporter. For this, we have employed information theoretic measures which provide objectivity in scoring the differentially conserved residues between two contrasting families [18]. While it is intuitive to select these conserved residues across the subfamily, by a visual analysis of a multiple alignment, however, one tends to miss out the residues which may have a lower conservation but still are functionally important. As CaMdr1p is an antiporter and belongs to DHA1 family, we contrasted DHA1 (antiporters) and SP family (symporters) to identify residues differentially conserved between these two families. We then scaled the CRE across the two families with the conservation in DHA1 which helped us to enlist the alignment positions on the basis of high CRE as well as high conservation across the entire DHA1 family. This enabled us to identify residues conserved exclusively in DHA1 and those which were differentially conserved among the two families.

We selected thirty highest CRE_S_ scoring residues and further short-listed those which were present in CaMdr1p. The 19 such residues of CaMdr1p D235, G264, P261, G133, A231, G472, V364, Y369, G391, G515, M132, F277, P139, L246, P512, S232, S505, P257 and V496 were subjected to site-directed mutagenesis. Our analysis revealed that all the mutant variants except P139A displayed decreased resistance to the tested drugs though at varying degree and specificity. This ranged from having twelve mutant variants D235A, G264L, P261A, G133L, G472L, Y369A, G391L, G515L, F277A, L246A, S232A and P257A showing hypersensitivity to all the drugs, to six variants A231G, V364A M132A, P512A, S505A and V496A which displayed loss in resistance to only selective drugs. The efflux of MTX, FLU and NR by mutant variants was abrogated which matched well with the loss in drug resistance. We ensured that such changes were not related to poor expression and surface localization of mutant variant proteins (Figure 4).

We predict that the residues with high CRE_S_ scores will have a role in drug-proton antiporter function of CaMdr1p. As the CRE_S_ scores decrease, the residues might not turn out to be important for drug-proton antiport function. CRE_S_ calculations also assign a low score to residues which are identically conserved in both these families. Such residues are shown to be MFS-wide-function-specific in our previous study [8]. Thus, we hypothesize that residues having high CRE_S_ score are critical for drug-proton antiport function while residues with low CRE_S_ scores are not expected to be critical for drug-proton antiport function but may be important for family-wide function. For example, we have earlier shown that residues G165, R215 and P296 are involved in MFS-wide functions and this study shows that these residues have low CRE_S_ scores. Thus, these residues are predicted to be critical for MFS-wide functions such as inter-helical interactions but are not involved directly in drug-proton antiport function of CaMdr1p [8].

As an exception, our criteria of CRE_S_ also picked up two residues D235 and F277, which were earlier reported to be family-wide function-specific [8]. This could probably be because our program does not discriminate between the nature of amino acid present in DHA1 and SP families at a given alignment position. Some positions that are scored using this method may be actually excluded using our knowledge of amino acid similarity. For example, at position 235 of CaMdr1p, an aspartate is present. However, a glutamate occurs with same frequency at the respective alignment position in the SP family. This has been identified to be differentially conserved in DHA1 and SP. Another exception, P139, though predicted to be important, failed to show any effect when replaced with alanine.

**Figure 6:**
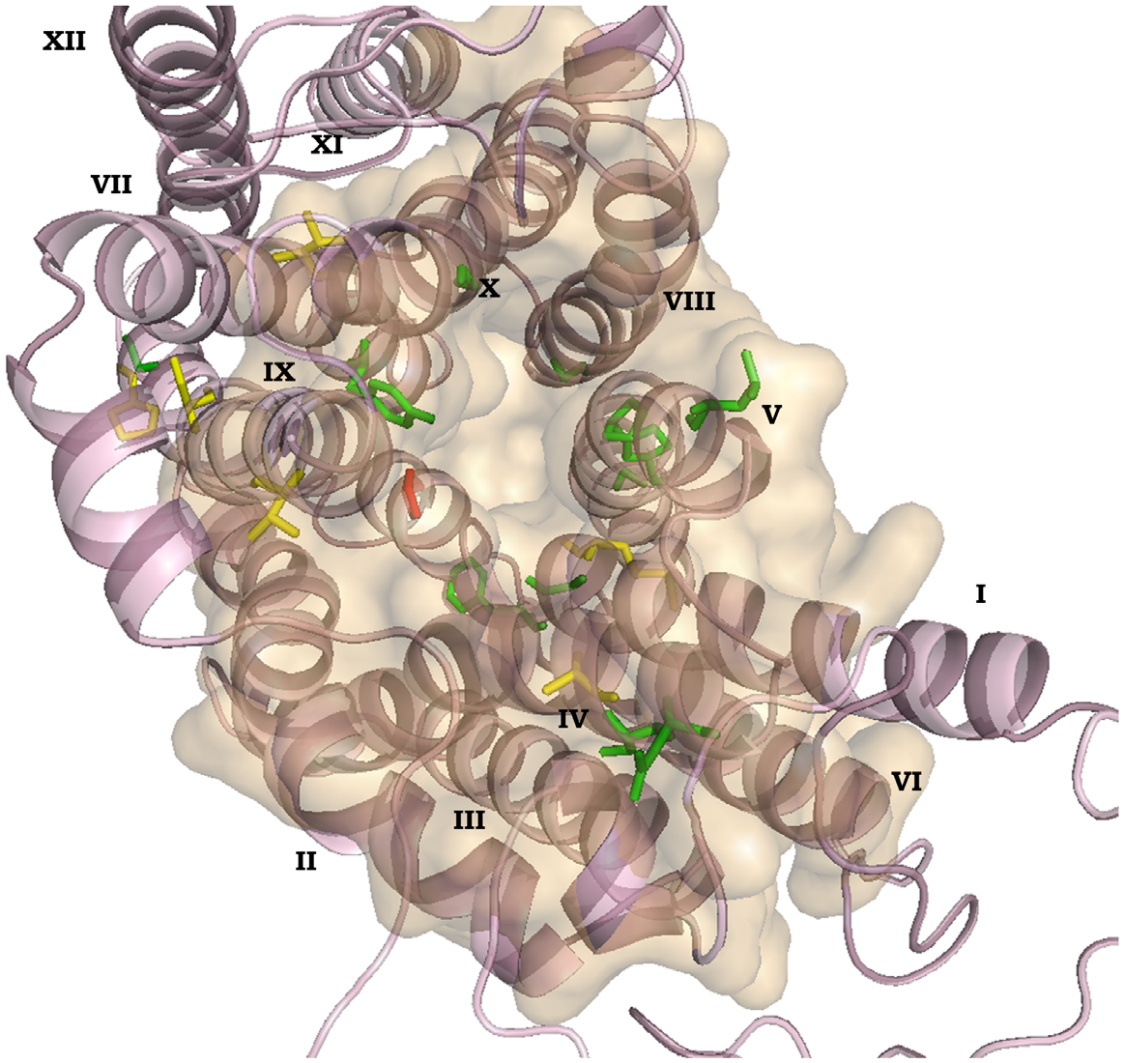
The homology model of CaMdr1p highlighting the 3D location of the mutated residue positions. The 3D homology model of CaMdr1p wherein the mutated positions are marked onto the model and are coloured on the basis of the phenotypes exhibited upon mutation. Green denotes sensitive, red shows resistant while yellow marks those which exhibit differential sensitivity towards drugs upon mutation. The twelve transmembrane segments (TMS) of CaMdr1p are numbered and are shown as α-helices. The pore is highlighted which clearly exhibits that the mutated positions lie in or around the pore. The structure is viewed using pymol v0.99 (http://www.pymol.org).

Our study puts forward a list of residues important for DHA1, drug-proton antiporters, when compared against SP, a symporter family. Since functionally critical residues of CaMdr1p are predicted to be DHA1 family specific, we validated this by comparing observations from previously published studies. In VAChT, a human vesicular acetylcholine transporter, a member of DHA1 family, six glycine and proline rich motifs are predicted to promote the formation of special backbone conformations including kinks in TMS, tight interactions between TMS and very flexible β-turns. The hypothesized rocking motions in the MFS family presumably require conformational changes in TMS and β-turns which occur due to the presence of these kinks and notches in the protein [19]. Interestingly, an analysis of the twelve mutant variants of CaMdr1p which displayed complete sensitivity to all the drugs, and also show abrogated efflux, reveals that most of these are glycines and prolines. Taken together, it can be hypothesized that these residues might be important for functions such as proton-antiport, rocker-switch mechanism or drug binding and translocation and if replaced, would result in a non-functional protein displaying susceptibility to all the drugs. This high propensity for kinks and notches are robustly predicted for motif D2 (lgxxxxxPvxP), motif C (gxxxGPxxGGxl) and motif C' (GxxxGPL). Interestingly, most of these nineteen residues of CaMdr1p with high CRE_S_ turned out to be part of the well-known motifs of the antiporters. These motifs are identified as Motif C and Motif C'. It is known that the two halves of the protein must have been formed due to a gene duplication event and thus Motif C' of C-terminal is degenerate as compared to Motif C of N-terminal. Four out of the nineteen residues L246, P257, P261 and G264 are a part of Motif C, a characteristic motif of the antiporters [20], while residues S505, P512 and G515 are a part of Motif C'. Their corresponding positions in VAChT, L214, S225, P229 and G232 in Motif C and V420, P436 and A439 in Motif C' are predicted to form a notch which allows two helical TMS to approach each other closely because small side chains are located at the interface. Motif D2 is located where it might help block non-specific leakage of protons. It is known to hold a kink, which might be involved in bend or swivel during conformational transitions, also called as “molecular hinges”. M132 and G133 of CaMdr1p lie in this motif D2. Notably, different levels of sensitivity seen in six other mutant variants A231G, V364A, M132A, P512A, S505A and V496A points to the fact that even though there is a single pore for proton antiport and drug binding and translocation, yet there are residues which impart selectivity in drug recognition along with those which affect the transport of all the drugs. Of note, most of these residues with high CRE_S_ are located in or near the pore in the 3D homology model of CaMdr1p, thus validating their relevance (Figure 6). This 3D homology model was deduced on the basis of crystal structures of MFS transporters such as lac permease of *E. coli* (1pv6), glycerol-3-phophate of *E. coli* (1pw4) and oxalate: formate transporter of *O. formigenes* (1zc7), as described in our previous study [8]. The application of our method to another MFS-MDR transporter EmrD of *E. coli* further reveals that residues which are shown to be functionally critical were found to have high CRE_S_ score and could be matched with critical residues of CaMdr1p. For example, P257, P261 and S505 of CaMdr1p which correspond to P145, P149 and G340 of EmrD, respectively, are predicted to be non-cavity lining residues as explained by the known crystal structure of EmrD [21]. Thus, our predictions are validated by CaMdr1p and may be extended across the DHA1 family.

**Figure 7:**
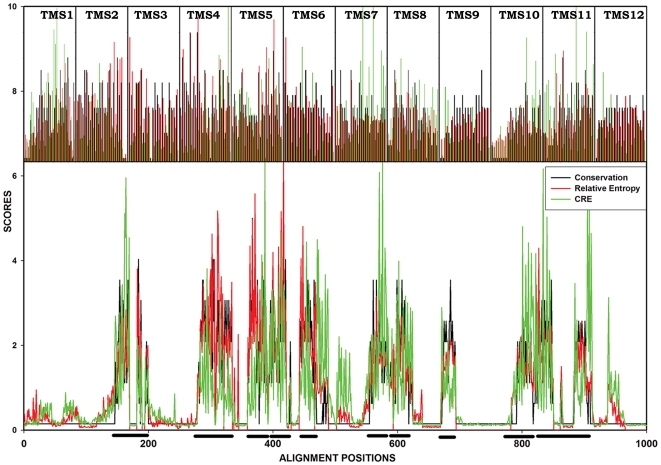
Comparative plot of Conservation, RE_NULL_ and CRE_S_ across the entire alignment. The three scores: Conservation in DHA1 family (black), RE_NULL_ across DHA1 family (red) and CRE_S_ (green) are drawn as a line graph for all the alignment positions in the bottom panel of the Figure. Locations of the TMS are marked by black bars on the x-axis. These twelve TMS are shown separately as an inset in the top panel so to highlight the effectiveness of CRE_S_ over RE and traditional conservation. The conservation scores remain almost constant throughout the TMS while RE_NULL_ is more prominent in the N-terminal half of the protein. High CRE_S_ positions span all the TMS, thus showing the functionally relevant residues to be scattered over both the halves of the protein.

Conservation, RE_NULL_ and CRE_S_ are three different scores which are routinely used to predict functionally critical residues of a protein. Figure 7 shows a comparison among them and reveals an interesting correlation. Traditional conservation, which uses amino acid similarity, selects residues indiscriminately across the transmembrane regions of the protein. These regions are predominantly hydrophobic, as required for optimal positioning within the hydrophobic environment of the lipid membrane. RE_NULL_ helps in subtracting these background conservation signals but can allow us to select only the residues which are important for family-wide-function [8]. Discernible from the Figure 7, is that the RE_NULL_ of DHA family is higher in the N-terminal half as compared to the C-terminal half of the protein, which supports the hypothesis that MFS proteins after duplication, have concentrated family-wide-function specific residues in the N-terminal half, while allowing divergence in the C-terminal half for more specific function such as substrate binding and translocation [6]. A comparison of an antiporter-specific alignment with a symporter-specific alignment further helped us to subtract MFS family-wide signals, so as to highlight residues, responsible for antiporter specificity. From this differential comparison, a concentration of signals around TMS 5-a known “antiporter motif” is seen where residues are highlighted by the use of CRE_S_ and this provides internal validation for the method. Other high-scoring residues are distributed over both the N-terminal and the C-terminal halves thus satisfying the earlier observation that substrate specificity is contributed by residues in the C-terminal, and providing a much sought after selection of novel residues, which we predict are important for drug antiporter function. In conclusion, we show that by using an information theoretic measure, it is possible to rationally conduct structure and functional study of a major MFS antiporter Mdr1p of *Candida albicans*. The function-specific residues identified for the entire DHA1 family are validated by CaMdr1p. These residues are predicted to be responsible for subfamily-specific functions such as drug binding and transport and proton translocation. Our analyses predict that few residues in or around the pore differ on the basis of the mechanism and substrate specificity of an MFS transporter and are thus predicted to be specific for a particular subfamily.

## Materials and Methods

### Materials

Anti-GFP monoclonal antibody was purchased from BD Biosciences Clontech, Palo Alto, CA, USA. DNA modifying enzymes were purchased from NEB. The drugs cycloheximide (CYH), 4-Nitroquinoline oxide (4-NQO), Methotrexate (MTX), Cerulenin (CER), Anisomycin (ANISO), Nile Red (NR) and Protease inhibitors (Phenylmethylsulfonyl fluoride, Leupeptin, Aprotinin, Pepstatin A, TPCK, TLCK) and other molecular grade chemicals were obtained from Sigma Chemicals Co. (St. Louis, MO, USA). Fluconazole (FLU) was generously provided by Ranbaxy Laboratories, India. [^3^H] Fluconazole was custom prepared and [^3^H] Methotrexate (MTX) was purchased from Amersham Biosciences, United Kingdom.

### Media and Strains

Plasmids were maintained in *Escherichia coli* DH5α. *E.coli* was cultured in Luria-Bertani medium (Difco, BD Biosciences, NJ, USA) to which ampicillin was added (100 µg/ml). The *Saccharomyces cerevisiae* strain used was AD1-8u^-^ (*MAT*a *pdr1-3 his1 ura3* Δ*yor1*::*hisG* Δ*snq2*::*hisG* Δ*pdr5*::*hisG* Δ*pdr10*::*hisG* Δ*pdr11*::*hisG* Δ*ycf1*::*hisG* Δ*pdr3*::*hisG* Δ*pdr15*::*hisG*), provided by Richard D. Cannon, University of Otago, Dunedin, New Zealand. The yeast strains used in this study are listed in the Supplementary Data S3 [22], [23]. The yeast strains were cultured in YEPD broth (Bio101, Vista, CA, USA) or in SD-ura^-^ dropout media (0.67% yeast nitrogen base, 0.2% dropout mix, and 2% glucose; Difco). For agar plates, 2.5% (w/v) Bacto agar (Difco, NJ, USA) was added to the medium.

### Methods

#### Multiple Sequence Alignment (MSA)

MFS sequences belonging to DHA1 (Drug-Proton Antiporters Class I) and SP (Sugar Porter) families were extracted from TCDB (http://www.tcdb.org/index.php). The resultant dataset thus included 37 members of DHA1 and 44 members of SP. Redundant sequences, if any, were identified using blastclust [24], by setting the identity threshold S to 90, L to 0.9 and keeping all other parameters to default values. The DHA1 and SP sequences were then aligned separately by PRALINETM [25] using TMHMM [26] as the method for predicting TM lengths and keeping all other parameters at their default values. These resulting separate DHA1 and SP PRALINETM alignments were then aligned together by using the profile-profile alignment option of MUSCLE [27]. Conservation of amino acids in a column, for the entire dataset and for each family individually, was calculated using the method described by Livingstone *et al* [28].

#### Calculation of Scaled Cumulative Relative Entropy (CRE_S_) score

Alignments for each of these two families were extracted individually from the complete MSA. HMMbuild program of HMMER (http://hmmer.wustl.edu) was used to build profiles for these individual alignments. The probability of occurrence of each amino acid is read from the HMM profiles, for each alignment position and for both the subfamilies [29].

Relative Entropy (RE) is calculated as the deviation of the amino acid distribution (for a particular column) of one subfamily from that of another subfamily in the complete alignment. RE for the two subfamilies y_1_ and y_2_ at column position *i*, is given by



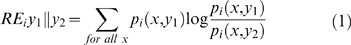
 where, **p_i_(x, y_n_)** is the probability of the occurrence of amino acid ***x*** in subfamily **y_n_** at column ***i*** of the MSA

RE_NULL_ for subfamily Y at column position i is given by



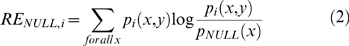
 p_NULL_(x) is the background probability of the amino acid, based on its occurrence in SWISSPROT. RE_NULL,*i*_ gives the conservation of column ***i*** with respect to the background probability distribution of amino acids.

While CRE at column *i* is given by the Kullback-Liebler distance between the corresponding *i*th columns



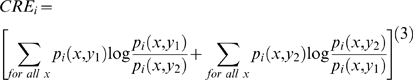



A classical description of entropy does not take into consideration the absence of data points which are gaps in the case of a multiple alignment column. Normalized CRE_i_ scores were obtained using a scaling factor which is equal to number of amino acid positions excluding gaps in column ***i*** divided by total number of sequences [8].

To select the alignment positions on the basis of both, high conservation across DHA1 and high CRE_i_, the normalized CRE_i_ was further scaled with respect to the RE_NULL_ as given by Equation 2. This was referred to as CRE_S_ and represented as per the following equation.
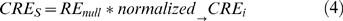
All calculations were made using PERL scripts, written in-house.

#### Site-directed mutagenesis of CaMdr1p

Site-directed mutagenesis was performed by using the Quick-Change Mutagenesis kit (Stratagene, La Jolla, CA, USA) as described previously. The mutations were introduced into the plasmid pRPCaMDR1-GFP according to the manufacturer's instructions, and the desired nucleotide sequence alterations were confirmed by DNA sequencing of the ORF. The primers used for the purpose are listed in Supplementary Data S4. The mutated plasmid, after linearising with *Xba1*, was used to transform AD1-8u^-^ cells for uracil prototrophy by lithium acetate transformation protocol [4]. Integration was confirmed by Southern Blot analysis (data not shown).

#### Drug Susceptibility

The susceptibilities of yeast cells, harbouring wild type CaMDR1-GFP and its mutant variants, was tested to different drugs by two independent methods: microdilution assay (liquid medium) and spot assays (solid medium). The MIC_80_ values for the strains were determined with a broth microdilution method as described earlier [30]. For spot assay, 5 µl samples of five-fold serial dilutions of yeast culture each with cells suspended in normal saline to an OD_600_ of 0.1 (1×10^6^ cells) at A_600_ were spotted onto YEPD plates in the absence (control) or in the presence of the drugs. Growth differences were recorded following incubation of the plates for 48 hrs at 30°C.

#### Immunodetection of CaMdr1p and its mutant variants

The plasma membranes (PM) were prepared from *S. cerevisiae* cells, as described previously [4]. The PM protein concentration was determined by bicinchonic acid assay using bovine serum albumin as the standard. For Western Blot analysis, the immunoblot was incubated with anti-GFP monoclonal antibody (1∶5000) (JL-8) (BD Biosciences) as described previously. Immunoreactivity of GFP antibody was detected using goat anti-mouse horseradish peroxidase-labelled antibody (1∶5,000) and was visualized using the enhanced chemiluminescence assay system (ECL kit, Amersham Biosciences, Arlington Heights, IL, USA) [4].

#### Transport of Nile Red

The accumulation of NR in cells expressing WT or mutant variant CaMdr1p-GFP was measured by flow cytometry with a FACSort flow cytometer (Becton-Dickinson Immunocytometry Systems, San Jose, Calif.) [18]. Briefly 0.1 OD_600_ cells were inoculated and were incubated at 30°C with shaking until the cultures reached an OD_600_ of 0.25. Cells were then harvested to be resuspended as 5% cell suspension in diluted medium (containing one part YEPD and two parts water). NR was added to a final concentration of 7 µM and cells were incubated in shaking water bath at 150 rpm at 30°C for 30 minutes. The cells were then harvested, washed and then resuspended in the diluted medium. Ten thousand cells were analyzed in acquisition. Analysis was performed with CellQuest software (Becton-Dickinson Immunocytometry Systems). The mean fluorescence intensity was calculated using the histogram stat program.

#### Transport of [^3^H] MTX and [^3^H] FLU

The accumulation of [^3^H] MTX (specific activity, 8.60 Ci/mmol) and that of [^3^H] FLU (specific activity, 19 Ci/mmol) was determined by protocol described previously [8]. Cells from mid-log phase were centrifuged at 500× g for 3 min and resuspended in fresh YEPD medium as 5% cell suspension. 100 µl of cell suspension was incubated in shaking water bath at 150 rpm at 30°C and then the radiolabelled drugs were added. The cells were incubated in either [^3^H] MTX (25 µM) or [^3^H] FLU (100 nM) for 30 min, filtered rapidly and washed twice with 1x PBS, pH 7.4 on Millipore manifold filter assembly using 0.45 µm nitrocellulose filter discs (Millipore, U.S.A). The filter discs were dried and put in cocktail-O and the radioactivity was measured in a liquid scintillation counter (Packard, Beckman, USA). The accumulation was expressed relative to the WT- CaMDR1-GFP.

## Supporting Information

Supplementary Data S1 The PRALINETM alignment of 34 sequences of DHA1 and 44 sequences of SP families as described in Materials and Methods.
(0.23 MB PDF)

Supplementary Data S2 RE across DHA1 and SP families, CRE and CRE_S_ scores for the entire MSA.
(1.32 MB DOC)

Supplementary Data S3 List of yeast strains used in this study.
(0.05 MB DOC)

Supplementary Data S4 List of oligonucleotides used for site-directed mutagenesis.
(0.06 MB DOC)

## References

[1] Prasad R, Kapoor K (2005). Multidrug resistance in yeast *Candida*.. Int Rev Cytol.

[2] Gaur M, Choudhury D, Prasad R (2005). Complete inventory of ABC proteins in human pathogenic yeast, *Candida albicans*.. J Mol Microbiol Biotechnol.

[3] Gaur M, Puri N, Manoharlal R, Rai V, Mukhopadhayay G (2008). MFS transportome of the human pathogenic yeast *Candida albicans*.. BMC Genomics.

[4] Saini P, Prasad T, Gaur NA, Shukla S, Jha S (2005). Alanine scanning of transmembrane helix 11 of Cdr1p ABC antifungal efflux pump of *Candida albicans*: identification of amino acid residues critical for drug efflux.. J Antimicrob Chemother.

[5] Smriti, Krishnamurthy S, Dixit BL, Gupta CM, Milewski S (2002). ABC transporters Cdr1p, Cdr2p and Cdr3p of a human pathogen *Candida albicans* are general phospholipid translocators.. Yeast.

[6] Paulsen IT, Brown MH, Skurray RA (1996). Proton-dependent multidrug efflux systems.. Microbiol Rev.

[7] Law CJ, Maloney PC, Wang DN (2008). Ins and outs of major facilitator superfamily antiporters.. Annu Rev Microbiol.

[8] Kapoor K, Rehan M, Kaushiki A, Pasrija R, Lynn AM (2009). Rational mutational analysis of a multidrug MFS transporter CaMdr1p of *Candida albicans* by employing a membrane environment based computational approach.. PLoS Comput Biol.

[9] Valdar WS (2002). Scoring residue conservation.. Proteins.

[10] Kimura M (1979). The neutral theory of molecular evolution.. Sci Am.

[11] Shannon CE (1948). The mathematical theory of communication.. Bell System Technical Journal.

[12] Sander C, Schneider R (1991). Database of homology-derived protein structures and the structural meaning of sequence alignment.. Proteins.

[13] Durbin R, Eddy E, Krogh A, Mitchison G (1998). Biological Sequence Analysis: Probabilistic Models of Prteins and Nucleic Acid..

[14] Ohno S (1970). Evolution by Gene Duplications..

[15] Li L, Shakhnovich EI, Mirny LA (2003). Amino acids determining enzyme-substrate specificity in prokaryotic and eukaryotic protein kinases.. Proc Natl Acad Sci U S A.

[16] Hannenhalli SS, Russell RB (2000). Analysis and prediction of functional sub-types from protein sequence alignments.. J Mol Biol.

[17] Ivnitski-Steele I, Holmes AR, Lamping E, Monk BC, Cannon RD (2009). Identification of Nile red as a fluorescent substrate of the *Candida albicans* ATP-binding cassette transporters Cdr1p and Cdr2p and the major facilitator superfamily transporter Mdr1p.. Anal Biochem.

[18] Chakrabarti S, Bryant SH, Panchenko AR (2007). Functional specificity lies within the properties and evolutionary changes of amino acids 2.. J Mol Biol.

[19] Chandrasekaran A, Ojeda AM, Kolmakova NG, Parsons SM (2006). Mutational and bioinformatics analysis of proline- and glycine-rich motifs in vesicular acetylcholine transporter.. J Neurochem.

[20] Ginn SL, Brown MH, Skurray RA (2000). The TetA(K) tetracycline/H(+) antiporter from *Staphylococcus aureus*: mutagenesis and functional analysis of motif C.. J Bacteriol.

[21] Jeon J, Yang JS, Kim S (2009). Integration of evolutionary features for the identification of functionally important residues in major facilitator superfamily transporters.. PLoS Comput Biol.

[22] Nakamura K, Niimi M, Niimi K, Holmes AR, Yates JE (2001). Functional expression of *Candida albicans* drug efflux pump Cdr1p in a *Saccharomyces cerevisiae* strain deficient in membrane transporters.. Antimicrob Agents Chemother.

[23] Decottignies A, Grant AM, Nichols JW, De Wet H, McIntosh DB (1998). ATPase and multidrug transport activities of the overexpressed yeast ABC protein Yor1p.. J Biol Chem.

[24] Altschul SF, Gish W, Miller W, Myers EW, Lipman DJ (1990). Basic local alignment search tool.. J Mol Biol.

[25] Pirovano W, Feenstra KA, Heringa J (2008). PRALINETM: a strategy for improved multiple alignment of transmembrane proteins.. Bioinformatics.

[26] Krogh A, Larsson B, von HG, Sonnhammer EL (2001). Predicting transmembrane protein topology with a hidden Markov model: application to complete genomes.. J Mol Biol.

[27] Edgar RC (2004). MUSCLE: multiple sequence alignment with high accuracy and high throughput.. Nucleic Acids Res.

[28] Livingstone CD, Barton GJ (1993). Protein sequence alignments: a strategy for the hierarchical analysis of residue conservation.. Comput Appl Biosci.

[29] Srivastava PK, Desai DK, Nandi S, Lynn AM (2007). HMM-ModE–improved classification using profile hidden Markov models by optimising the discrimination threshold and modifying emission probabilities with negative training sequences.. BMC Bioinformatics.

[30] Pasrija R, Banerjee D, Prasad R (2007). Structure and function analysis of CaMdr1p, a major facilitator superfamily antifungal efflux transporter protein of *Candida albicans*: identification of amino acid residues critical for drug/H+ transport.. Eukaryot Cell.

[31] Barton GJ (1993). ALSCRIPT: a tool to format multiple sequence alignments.. Protein Eng.

