# Supplementary material for: Employing Information Theoretic Measures and Mutagenesis to Identify Residues Critical for Drug-Proton Antiport Function in Mdr1p of Candida albicans
Source: PLoS One. 2010 Jun 10;5(6):e11041. doi: 10.1371/journal.pone.0011041 (PMC2883579; doi:10.1371/journal.pone.0011041)
Supplement: Supplementary Data S1 — The PRALINETM alignment of 34 sequences of DHA1 and 44 sequences of SP families as described in Materials and Methods. (0.23 MB PDF) [file pone.0011041.s001.pdf]

The PRALINETM alignment of 34 sequences of DHA1 and 44 sequences of SP families as described in Materials and Methods.

2.A.1.2.1   ----------MASKIASLFSPSETASKDQHENVAE-------------------------

2.A.1.2.2   --MAAFIKDSFWGQIIYRLS--GRKLFRHNDELPDYVVPEKYLLD---------------

2.A.1.2.6   -MHYRFLRDSFVGRVTYHLS--KHKYFAHPEEAKNYIIPEKYLADYKPTLADDTSINFEK

2.A.1.2.17   MVYTSTYRHTIVVDLLEYLG--IVSNLETLQSAREDETRKPENTD--------------K

2.A.1.2.35   ---MGFLRETVFGELVEYVS--HNSNDNDLEKLR--------------------------

2.A.1.2.16   MSDHSPISNKENHLLPSDSS--RSSSSDMHSTGTTGTTGVEPVDFTGEGAKYTTATEGNG

2.A.1.2.40   MGSEPFQKKNLGLQINSQESGTTRSTFHSLEDLGDDVINESWDQVNQKRA---------N

2.A.1.2.31   ---M

2.A.1.2.3   --

2.A.1.2.4   --

2.A.1.2.38   --

2.A.1.2.39   --

2.A.1.2.41   --

2.A.1.2.8   --

2.A.1.2.10   --

2.A.1.2.24   --

2.A.1.2.20   --

2.A.1.2.34   --

2.A.1.2.7   --

2.A.1.2.27   --

2.A.1.2.19   --

2.A.1.2.14   --

2.A.1.2.15   --

```
2.A.1.2.18      ------------------------------------------------------------
2.A.1.2.25      ------------------------------------------------------------
2.A.1.2.26      ------------------------------------------------------------
2.A.1.2.11      ------------------------------------------------------------
2.A.1.2.12      ------------------------------------------------------------
2.A.1.2.29      ------------------------------------------------------------
2.A.1.2.28      ------------------------------------------------------------
2.A.1.2.30      ------------------------------------------------------------
2.A.1.2.9       ------------------------------------------------------------
2.A.1.2.22      ------------------------------------------------------------
2.A.1.2.32      ------------------------------------------------------------
2.A.1.2.21      ------------------------------------------------------------
2.A.1.2.37      ------------------------------------------------------------
2.A.1.2.5       ------------------------------------------------------------
2.A.1.1.1       ------------------------------------------------------------
2.A.1.1.2       ------------------------------------------------------------
2.A.1.1.26      ------------------------------------------------------------
2.A.1.1.53      ---------------------------------MASTFIQADSP----------------
2.A.1.1.55      ---------------------------------------MKNTP----------------
2.A.1.1.25      --------------------------MGERRRKQPEPDAASAAG----------------
2.A.1.1.63      -----------------------------MEGGIIHGGADESAF----------------
2.A.1.1.66      --------------------------MTLTIPNAPGSSGYLDMF----------------
2.A.1.1.32      ------------------------------------------------------------
2.A.1.1.35      ------------------------------------------------------------
2.A.1.1.65      -----------------------------MNVIGITLLPRGRIM---------------S
2.A.1.1.6       ---------------------------MAVEENNMPVVSQQPQAGEDVISSLSKDSHLSA
```

```
2.A.1.1.67    ---------------------------MSQDSHSSGAATPVNGS----------------
2.A.1.1.36    --------------------------MGVDIKGLFKPKAEQQEH----------------
2.A.1.1.58    ------------------------MGVSNMMSRFKPQADHSESS----------------
2.A.1.1.19    -----------------------------MNDSQNCLRQREENS----------------
2.A.1.1.8     -----------------------------MGIHIPYLTSKTSQS----------------
2.A.1.1.21    ------------------------------------------------------------
2.A.1.1.23    ------------------------------------------------------------
2.A.1.1.42    -------------------------------------MTTTTAS----------------
2.A.1.1.33    ---------------------------MSSNLSEKAVDQQKEFG----------------
2.A.1.1.18    --------------------------MKNLSFLINRRKENTSDS----------------
2.A.1.1.20    ------------------------------------------------------------
2.A.1.1.14    ------------------------------------MAGGGVVV----------------
2.A.1.1.50    ----------------------------------------MTGG----------------
2.A.1.1.60    ----------------------------------------MPAG----------------
2.A.1.1.61    ---------------------------------------MAGGA----------------
2.A.1.1.56    ------------------------------------------MA----------------
2.A.1.1.37    -----------------------------------------------------------M
2.A.1.1.47    ------------------------------------------------------------
2.A.1.1.52    ------------------------------------------------------------
2.A.1.1.51    ------------------------------------MGLEDNRM----------------
2.A.1.1.57    -------------------------------------------M----------------
2.A.1.1.28    ------------------------------------------------------------
2.A.1.1.29    ------------------------------------------------------------
2.A.1.1.41    ------------------------------------------------------------
2.A.1.1.54    -----------------------------------------------------------M
2.A.1.1.24    ------------------------------------------------------------
```

```
2.A.1.1.43    --------------------------MNPETSDKNEPIISSQPAANDDDDNNNTEVKEKQ

2.A.1.1.3     ------------------------------------------------------------

2.A.1.1.4     ------------------------------------------------------------

2.A.1.1.46    ------------------------------------MSPEDPQE----------------

2.A.1.1.59    ------------------------------------------------------------

2.A.1.1.17    --------------------------MTERRDNVSHAPDAIEGP----------------



2.A.1.2.1     ---DLELGTASSQSDGIHETNSEYDEKK-----------------------------REE

2.A.1.2.2     PKEEVLNSSDKSQSSENKEQTEGDQATI-----------------------------QNE

2.A.1.2.6     EEIDNQGEPNSSQSSSSNNTIVDNNNNN----------------------------NDND

2.A.1.2.17    KECKPDYDIECGPNRSCSESSTDSDSSG-----------------------------SQI

2.A.1.2.35    -----VWKDISKVDSNDSCSSESGDLKI-----------------------------HEV

2.A.1.2.16    GADLAIQRTTTMNSAAESEVNITRRLTKILTGSVNEPDRVEVDYTNCAPMGGDRPYPPSL

2.A.1.2.40    IDHDVFHEHPDSSPSLSAQKAKTKEEEV-----------------------------AVK

2.A.1.2.31    AGATSSIIRENDFEDELAESMQSYNRET----------------------------ADKL

2.A.1.2.3     ------------------------------------------------------------

2.A.1.2.4     ------------------------------------------------------------

2.A.1.2.38    ------------------------------------------------------------

2.A.1.2.39    ------------------------------------------------------------

2.A.1.2.41    ------------------------------------------------------------

2.A.1.2.8     ------------------------------------------------------------

2.A.1.2.10    ------------------------------------------------------------

2.A.1.2.24    ------------------------------------------------------------

2.A.1.2.20    ------------------------------------------------------------

2.A.1.2.34    ------------------------------------------------------------
```

```
2.A.1.2.7     ------------------------------------------------------------
2.A.1.2.27    ------------------------------------------------------------
2.A.1.2.19    ------------------------------------------------------------
2.A.1.2.14    ------------------------------------------------------------
2.A.1.2.15    ------------------------------------------------------------
2.A.1.2.18    ------------------------------------------------------------
2.A.1.2.25    ------------------MRVSTRNRKI-------------------------------L
2.A.1.2.26    ------------------------------------------------------------
2.A.1.2.11    ------------------------------------------------------------
2.A.1.2.12    ------------------------------------------------------------
2.A.1.2.29    ------------------------------------------------------------
2.A.1.2.28    --------------------------ME-----------------------------SAE
2.A.1.2.30    ------------------------------------------------------------
2.A.1.2.9     ------------------------------------------------------------
2.A.1.2.22    ------------------------------------------------------------
2.A.1.2.32    ------------------------------------------------------------
2.A.1.2.21    ------------------------------------------------------------
2.A.1.2.37    ------------------------------------------------------------
2.A.1.2.5     ------------------------------------------------------------
2.A.1.1.1     --------MPDAKKQGRSNK----------------------------------------
2.A.1.1.2     -MVTINTESALTPRSLRDTR----------------------------------------
2.A.1.1.26    -----------MNKQGNQMS----------------------------------------
2.A.1.1.53    ----EKSKKLPPLTEGPYRK----------------------------------------
2.A.1.1.55    ----TQLEPNVPVTRSHSMG----------------------------------------
2.A.1.1.25    ---ECSLLAAAESSTSLQSAGA--------------------------------------
2.A.1.1.63    ---KECFSLTWKNP----------------------------------------------
```

```
2.A.1.1.66    ---PERRMSYFGNS----------------------------------------------
2.A.1.1.32    --------MNPSSSPSQSTANV--------------------------------------
2.A.1.1.35    --MASTSQAPSPGAGTAHPDHL--------------------------------------
2.A.1.1.65    HGPVSDDTPSIFGDDDQAASSG--------------------------------------
2.A.1.1.6     QSQKYSNDELKAGESGSEGSQS--------------------------------------
2.A.1.1.67    ---ILEKEKEDSPVLQVDAPQK--------------------------------------
2.A.1.1.36    ---SQATTPSRTDSIAEKDNGI--------------------------------------
2.A.1.1.58    ---TEAPTPARSNSAVEKDNVL--------------------------------------
2.A.1.1.19    ---HLNPGNDFGHHQGAECTINHNNMPHRNAYTESTNDTEAKSIVMCDDPNAYQISYTNN
2.A.1.1.8     ---NVGDAVGNADSVEFNSEHDSPSKRGKITLESHEI-------------QRAPASDDED
2.A.1.1.21    ------------------------------------------------------------
2.A.1.1.23    ------------------------------------------------------------
2.A.1.1.42    ---PVSKQTASAAQETSATGA-----AATAIETIETG---------------VAGVAGAA
2.A.1.1.33    ---LLKSTSSVDSVHEVEAGVKVGEDDDNYFFSEDQILTQI---------KVYEDEIANT
2.A.1.1.18    ---NVYPGKAKSHEPSWIEMDDQTKKDGLDIVHVEFSPDTRAPS-----DSNKVITEIFD
2.A.1.1.20    ------------------------------------------------------------
2.A.1.1.14    ---VSGRGLSTGDYRGGLTV----------------------------------------
2.A.1.1.50    ---GFATSANGVEFEAKITP----------------------------------------
2.A.1.1.60    ---GFVVGDGQKAYPGKLTP----------------------------------------
2.A.1.1.61    ---FIDESGHGGDYEGRVTA----------------------------------------
2.A.1.1.56    ---VVVSNANAPAFEAKMTV----------------------------------------
2.A.1.1.37    ENKEAGTPPPIPSREGRLQP----------------------------------------
2.A.1.1.47    MKLSEKNSAETKESQRKWSF----------------------------------------
2.A.1.1.52    --------MGVASNNGITGK----------------------------------------
2.A.1.1.51    ----VKRFVNVGEKKAGSTA----------------------------------------
2.A.1.1.57    ----AEGFVDASRVEAPVTL----------------------------------------
```

2.A.1.1.28	--
2.A.1.1.29	--
2.A.1.1.41	------------------MR--
2.A.1.1.54	TDIKATSSTSATTAPTAGRP--
2.A.1.1.24	MTKSSKDICSENEGKKNGKSG---------------------------------------
2.A.1.1.43	DTKPTLDISTVGKNPDSKTAA---------------------------------------
2.A.1.1.3	------------MNTQYNSS--
2.A.1.1.4	------------MSSESSQG--
2.A.1.1.46	----TQPLLRPPEARTPRGR--
2.A.1.1.59	--
2.A.1.1.17	---NDGAHAEDTSPGFFSFEN---------------------------------------

2.A.1.2.1	SPEVIDISNLISSDHPAHPQNWHWAKRWSIVFMFCLMQIYVIWTSNGFG-----------
2.A.1.2.2	PASEHIIVTWDGDDDPENPYNWPFAWKAIAAMQIGFLTVSVYMASAIYT-----------
2.A.1.2.6	VDGDKIVVTWDGDDDPENPQNWPTLQKAFFIFQISFLTTSVYMGSAVYT-----------
2.A.1.2.17	EKNDPFRVDWNGPSDPENPQNWPLLKKSLVVFQIMLLTCVTYMGSSIYT-----------
2.A.1.2.35	NSEGHIIVRWDDANDPENPLNWPLWAKLVVTFDICFLTFAVYVGSAIFT-----------
2.A.1.2.16	PSRDLYEVTFDGPNDPLHPFNWPMKKKVLLCLVLCLDSIAIAMCSSIFA-----------
2.A.1.2.40	SSNSQSRDPSPDTQAHIPYTYFSKDQRLIIFGIIIFIGFLGPMSGNIYI-----------
2.A.1.2.31	ALTRTESVKPEPEITAPPHSRFSRSFKTVLIAQCAFTGFFSTIAGAIYY-----------
2.A.1.2.3	--------------------MSSKNFSWRYSLAATVLLLSPFDLLASLG-----------
2.A.1.2.4	------------------------MKPNRPLIVILSTVALDAVGIGLIM-----------
2.A.1.2.38	--------------------------MKKSLSVILITIFLDAVGIGLIM-----------
2.A.1.2.39	--------------------------MKKPMLVILLTVLLDAVGIGLIM-----------
2.A.1.2.41	---------------MTSPTSLTRRDQNRAWIMLIVLTMLTVIGMTVVL-----------

2.A.1.2.8	-------------------MKKSINEQKTIFIILLSNIFVAFLGIGLII-----------
2.A.1.2.10	--------------------------MNKQIFVLYFNIFLIFLGIGLVI-----------
2.A.1.2.24	-------------MLTQKIELEAKPKIPEEIWVLVVAAFIIALGYGLIA-----------
2.A.1.2.20	---------------MSPCENDTPINWKRNLIVAWLGCFLTGAAFSLVM-----------
2.A.1.2.34	---------------------MTEINWKDNLRIAWFGNFLTGASISLVV-----------
2.A.1.2.7	-------------------MTTRQHSSFAIVFILGLLAMLMPLSIDMYL-----------
2.A.1.2.27	-----------------MSRAASAPSYSLMMVMLGLLSCVAPASIDAYL-----------
2.A.1.2.19	------------MQNKLASGARLGRQALLFPLCLVLYEFSTYIGNDMIQ-----------
2.A.1.2.14	--------------------------MKKVILSLALGTFGLGMAEFGIM-----------
2.A.1.2.15	----------------MTTNTVSRKVAWLRVVTLAVAAFIFNTTEFVPV-----------
2.A.1.2.18	----------------MTSASPSRSTAWLRVVTLAIAAFIFNTTEFIPV-----------
2.A.1.2.25	ITKFVYDIFCNQDFFYLSKHLSKGVCYEFQSFPVAASTIAVGLVELIVG-----------
2.A.1.2.26	---------MSEFIAENRGADAITRPNWSAVFSVAFCVACLIIVEFLPV-----------
2.A.1.2.11	-------MLQVVLGAPQRLLKEGRQSRKLVLVVVFVALLLDNMLLTVVV-----------
2.A.1.2.12	-------MLRTILDAPQRLLKEGRASRQLVLVVVFVALLLDNMLFTVVV-----------
2.A.1.2.29	--------MALSELALVRWLQESRRSRKLILFIVFLALLLDNMLLTVVV-----------
2.A.1.2.28	PAGQARAAATKLSEAVGAALQEPRRQRRLVLVIVCVALLLDNMLYMVIV-----------
2.A.1.2.30	-------MPEKRAGAQAAGSTWLQGFGRPSVYHAAIVIFLEFFAWGLLT-----------
2.A.1.2.9	---------------------MKRQRNVNLLLMLVLLVAVGQMAQTIYI-----------
2.A.1.2.22	--------------------------MSRFLICSFALVLLYPAGIDMYL-----------
2.A.1.2.32	--------------------------MPLPLYLLAVAVCAMGTSEFMLA-----------
2.A.1.2.21	-------------------MSRVSQARNLGKYFLLIDNMLVVLGFFVVF-----------
2.A.1.2.37	---------------------MNKPVQKMMLSGQFLMILALEMTNPFLP-----------
2.A.1.2.5	--------------------MKEFWNLDKNLQLRLGIVFLGAFSYGTVF-----------
2.A.1.1.1	------------------------AMTFFVCFLAALAGLLFGLDIGVIA-----------
2.A.1.1.2	------------------------RMNMFVSVAAAVAGLLFGLDIGVIA-----------

2.A.1.1.26 -------------------------FLRTIILVSTFGGLLFGYDTGVLN-----------

2.A.1.1.53 -------------------------RLFYVALVATFGGLLFGYDTGVIN-----------

2.A.1.1.55 -------------------------FVILISCAAGLGGLLYGYDTAVIS-----------

2.A.1.1.25 --GGGGVGDLERAARRQFQQDETPAFVYVVAVFSALGGFLFGYDTGVVS-----------

2.A.1.1.63 -------------------------YVLRLAFSAGIGGLLFGYDTGVIS-----------

2.A.1.1.66 -------------------------YILGLTVTAGIGGLLFGYDTGVIS-----------

2.A.1.1.32 ------------------------KFVLLISGVAALGGFLFGFDTAVIN-----------

2.A.1.1.35 ------------------------GHVIFIAAAAAMGGFLFGYDSSVIN-----------

2.A.1.1.65 ------------------------RTAVRIAAVAALGGLLFGYDSAVIN-----------

2.A.1.1.6 -----------VPIEIPKKPMSEYVTVSLLCLCVAFGGFMFGWDTGTIS-----------

2.A.1.1.67 -----------GFK--------DYIVISIFCFMVAFGGFVFGFDTGTIS-----------

2.A.1.1.36 --------------IDDSPVKYLTWRSFILGIVVSMGGFIFGYSTGQIS-----------

2.A.1.1.58 --------------LDDSPVKYLTWRSFILGIVVSMGGFIFGYSTGQIS-----------

2.A.1.1.19 EPAGDGAIETTSILLSQPLPLRSNVMSVLVGIFVAVGGFLFGYDTGLIN-----------

2.A.1.1.8 RIQIKPVNDEDDTSVMITFNQSLSPFIITLTFVASISGFMFGYDTGYIS-----------

2.A.1.1.21 -----------------MGFKRGKNFTLVMLIFVSMAGWMFGADTGSIG-----------

2.A.1.1.23 ---------------------MNRFITSILVVFISMSGWLQGADTGSIS-----------

2.A.1.1.42 TNAAANAIEDLEAAESHGFSTRFPLNSAFIFTFGALGGMLFGFDTGIIS-----------

2.A.1.1.33 PVTKTLRQRMRRMEFCEVNFSNKTYMVIMLGFFASFAGILSGVDQSTIS-----------

2.A.1.1.18 ATEDAKEADESERGMPLATALNTYPKAAAWSLLVSTTLIMEGYDTAILG-----------

2.A.1.1.20 -------------------------MRASVMLCAALGGFLFGYDTGVIN-----------

2.A.1.1.14 -------------------------YVVMVAFMAACGGLLLGYDNGVTG-----------

2.A.1.1.50 -------------------------IVIISCIMAATGGLMFGYDVGVSG-----------

2.A.1.1.60 -------------------------FVLFTCVVAAMGGLIFGYDIGISG-----------

2.A.1.1.61 -------------------------FVMITCIVAAMGGLLFGYDIGISG-----------

2.A.1.1.56 -------------------------YVFICVMIAAVGGLIFGYDIGISG-----------

2.A.1.1.37 ------------------------TLLLATLSAAFGSAFQYGYNLSVVNTPHKVGTSCGW

2.A.1.1.47 ------------------------SLVVAALVGAFGSSFLYGYNLSVVN-----------

2.A.1.1.52 -------------------------LVLTVLITCVGSSFLIGYNLGVLN-----------

2.A.1.1.51 -------------------------MAIIVGLFAASGGVLFGYDTGTIS-----------

2.A.1.1.57 -------------------------KTYLMCAFAAFGGIFFGYDSGYIS-----------

2.A.1.1.28 ---------------MEPSSKKLTGRLMLAVGGAVLGSLQFGYNTGVIN-----------

2.A.1.1.29 -----------------MTEDKVTGTLVFTVITAVLGSFQFGYDIGVIN-----------

2.A.1.1.41 -----------------------KVSTGFVYFFGALGGLLFGYDTGVIS-----------

2.A.1.1.54 -----------------------ARRLGQISLVACLGGLLFGYDTGVAN-----------

2.A.1.1.24 ------------------F----FSTSFKYVLSACIASFIFGYQVSVLN-----------

2.A.1.1.43 ------------------L----PTFCLFVALVSVIGAFENGWNTSVSN-----------

2.A.1.1.3 -------------------------YIFSITLVATLGGLLFGYDTAVIS-----------

2.A.1.1.4 -------------------------LVTRLALIAAIGGLLFGYDSAVIA-----------

2.A.1.1.46 -------------------------RVFLASFAAALGPLSFGFALGYSS-----------

2.A.1.1.59 -------------------MGHSPPVLPLCASVSLLGGLTFGYELAVIS-----------

2.A.1.1.17 ------------------L-----GVAQVQVVGGTLNGFSIGFVAVYIL-----------

2.A.1.2.1 -SIEYS-VM-----AQFNV---

2.A.1.2.2 -PGVEE-IM-----NQFNI---

2.A.1.2.6 -PGIEE-LM-----HDFGI---

2.A.1.2.17 -PGQEY-IQ-----EEFHV---

2.A.1.2.35 -PGISE-MQ-----ETMHV---

2.A.1.2.16 -SAVPQ-IC-----EIYHV---

2.A.1.2.40 -PALPL-LQ-----REYDV---

2.A.1.2.31 -PVLSV-IE-----RKFDI---

2.A.1.2.3	-MDMYL-PA-----VPFMPNALG-------------------------------------
2.A.1.2.4	-PVLPG-LL-----RD-LV-HSN-------------------------------------
2.A.1.2.38	-PILPELLR-----SLAGAE--
2.A.1.2.39	-PILPALLR-----SLGGLD--
2.A.1.2.41	-PVLPF-VV-----LQYVS-HES-------------------------------------
2.A.1.2.8	-PVMPS-FM-----KIMHL---
2.A.1.2.10	-PVLPV-YL-----KDLGL---
2.A.1.2.24	-PILPQ-FV-----VGFDV---
2.A.1.2.20	-PFLPL-YV-----EQLGVTGHS-------------------------------------
2.A.1.2.34	-PFMPI-FV-----ENLGV-GSQ-------------------------------------
2.A.1.2.7	-PALPV-IS-----AQFGV---
2.A.1.2.27	-PAFGA-LQ-----REFGV---
2.A.1.2.19	-PGMLA-VV-----EQYQA---
2.A.1.2.14	-GVLTE-LA-----HNVGI---
2.A.1.2.15	-GLLSD-IA-----QSFHM---
2.A.1.2.18	-GLLSD-IA-----NSFAM---
2.A.1.2.25	-GILPQ-IA-----NDLDI---
2.A.1.2.26	-SLLTP-MA-----QDLGI---
2.A.1.2.11	-PIVPTFLY-----ATEFKDSNSSLHRGPSVSSQQALTSPAFSTIFSFFDNTTTTVEEHV
2.A.1.2.12	-PIVPTFLY-----DMEFKEVNSSLHLGHAGSSPHALASPAFSTIFSFFNNNTVAVEESV
2.A.1.2.29	-PIIPSYLY-----SIKHEKNATEIQTARPVHTASISDSFQSIFSYYDNSTMVTGNATRD
2.A.1.2.28	-PIVPDYIA-----HMRGGGEGPTRTPEVWEPTLPLPTPANASAYTANTSASPTAAWPAG
2.A.1.2.30	-TPMLT-VL-----HETFSQH---------------------------------------
2.A.1.2.9	-PAIAD-MA-----RDLNV---
2.A.1.2.22	-VGLPR-IA-----ADLNA---
2.A.1.2.32	-GLVPD-IA-----SDLGV---

```
2.A.1.2.21    -PLISIRFV-----DQMGW-----------------------------------------
2.A.1.2.37    -LLIAH-QS-----NTPVP-----------------------------------------
2.A.1.2.5     -SSMTI-YY-----NQYL------------------------------------------
2.A.1.1.1     -GALPF-IA-----DEFQ------------------------------------------
2.A.1.1.2     -GALPF-IT-----DHFV------------------------------------------
2.A.1.1.26    -GALPY-MG-----EPDQLN----------------------------------------
2.A.1.1.53    -GALNP-MT-----R--ELG----------------------------------------
2.A.1.1.55    -GAIGF-LK-----D--LYS----------------------------------------
2.A.1.1.25    -GAMLL-LK-----RQLS------------------------------------------
2.A.1.1.63    -GALLY-IR-----DDFKSVD---------------------------------------
2.A.1.1.66    -GALLY-IK-----DDFEVVK---------------------------------------
2.A.1.1.32    -GAVAA-LQ-----KHFQ------------------------------------------
2.A.1.1.35    -GAVEA-IR-----DRYD------------------------------------------
2.A.1.1.65    -GAVDS-IQ-----EDFG------------------------------------------
2.A.1.1.6     -GFVVQ-TD-----FLRRFGMKHKDGTHY-------------------------------
2.A.1.1.67    -GFVNM-SD-----FKDRFGQHHADGTPY-------------------------------
2.A.1.1.36    -GFTTM-AD-----FKKRFAERQANGEYV-------------------------------
2.A.1.1.58    -GFETM-DD-----FLQRFGQEQADGSYA-------------------------------
2.A.1.1.19    -SITDM-PY-----VKTYIAP--NHS----Y-----------------------------
2.A.1.1.8     -SALIS-IG-----TDLDHK----------V-----------------------------
2.A.1.1.21    -GVTSM-RD-----FRERYADRYDPITDQYS-----------------------------
2.A.1.1.23    -GILGM-RD-----FQSRFADRYNPISNSYS-----------------------------
2.A.1.1.42    -GASPL-------------------IESDFG-----------------------------
2.A.1.1.33    -GASYG-------------------MKASLK-----------------------------
2.A.1.1.18    -AFYAL-PI-----FQRKFGSQ-NDKTGEWE-----------------------------
2.A.1.1.20    -AALFQ-M----------------KDHFGFS-----------------------------
```

```
2.A.1.1.14    -GVVSL-EA-----FEKKFFPDVWAK-KQEVHEDSPYCT---------------------
2.A.1.1.50    -GVTSM-PD-----FLEKFFPVVYRKVVAGADKDSNYCK---------------------
2.A.1.1.60    -GVTSM-PS-----FLKRFFPSVYRKQQEDAST-NQYCQ---------------------
2.A.1.1.61    -GVISM-ED-----FLTKFFPDVLRQMQNKRGRETEYCK---------------------
2.A.1.1.56    -GVSAM-DD-----FLKEFFPAVWERKKHVHE--NNYCK---------------------
2.A.1.1.37    GNVFQV-FK-----SFYNETYFERHATFMDGK----------------------------
2.A.1.1.47    -APTPY-IK-----AFYNGTWYRRHGQPIDPD----------------------------
2.A.1.1.52    ------------------LPRRNIEIYFNETVVPNTPEL---------------------
2.A.1.1.51    -GVMTM-DY-----VLARY--------PSNKHS---------------------------
2.A.1.1.57    -GVMGM-RY-----FIEEFEGLDYNTTPTDSFV---------------------------
2.A.1.1.28    -APQKV-IEEFYNQTWVHRYGESILPT---------------------------------
2.A.1.1.29    -APQQV-IISHYRHVLGVPLDDRKAINNYVINSTDELPTISYSMNPKPTPWAEEETVAAA
2.A.1.1.41    -GAILF-IQ-----KQMN------------------------------------------
2.A.1.1.54    -GAEGH-MA-----QELG------------------------------------------
2.A.1.1.24    -TIKNF-IV-----VEFEWCKGEKDRL---NCS---------------------------
2.A.1.1.43    -VPEKI-IR-----DCKNGQKHVNIGVLPDCLP---------------------------
2.A.1.1.3     -GTVES-LN-----TVFVAPQNLSES----------------------------------
2.A.1.1.4     -AIGTP-VD-----IHFIAPRHLSAT----------------------------------
2.A.1.1.46    -PAIPS-LR-----RTA----PPALR----------------------------------
2.A.1.1.59    -GALLP-LQ-----LDFG------------------------------------------
2.A.1.1.17    -LYEVA-TN-----CSLFKTTEACKAVGSYGCEWKDTEVCSWKKECDSDSDGVNPCESLI


2.A.1.2.1     --------------------------------------SAQVATLCLSMNILGSGLGPMF
2.A.1.2.2     --------------------------------------NSTLATLPLTMFVIGYGIGPLF
2.A.1.2.6     --------------------------------------GRVVATLPLTLFVIGYGVGPLV
```

2.A.1.2.17 --------------------------------------GHVVATLNLSLYVLGYGLGPII

2.A.1.2.35 --------------------------------------GTVPVVLGLTLFVLGYAIGPLI

2.A.1.2.16 --------------------------------------IEVVAILGITLFVLGFAASPVI

2.A.1.2.40 --------------------------------------SATTINATVSVFMAVFSVGPLF

2.A.1.2.31 --------------------------------------DEELVNVTVVVYFVFQGLAPTF

2.A.1.2.3 -------------------------------------TTASTIQLTLTTYLVMIGAGQLL

2.A.1.2.4 -------------------------------------DVTAHYGILLALYALMQFACAPV

2.A.1.2.38 -------------------------------------AGGVHYGALLAVYALMQFIFAPI

2.A.1.2.39 -------------------------------------AGSVHYGALLAAYALMQFLFSPI

2.A.1.2.41 -------------------------------------DLAIWVGVLEAINGLCAFLVAPF

2.A.1.2.8 --------------------------------------SGSTMGYLVAAFAISQLITSPF

2.A.1.2.10 --------------------------------------TGSDLGLLVAAFALSQMIISPF

2.A.1.2.24 --------------------------------------SFAAASAVVSIFAGARLLFAPM

2.A.1.2.20 -------------------------------------ALNMWSGIVFSITFLFSAIASPF

2.A.1.2.34 -------------------------------------QVAFYAGLAISVSAISAALFSPI

2.A.1.2.7 --------------------------------------PAGSTQMTLSTYILGFALGQLI

2.A.1.2.27 --------------------------------------PQETVQLTLGVYMFCYAAMLLL

2.A.1.2.19 --------------------------------------GIDWVPTSMTAYLAGGMFLQWL

2.A.1.2.14 --------------------------------------SIPAAGHMISYYALGVVVGAPI

2.A.1.2.15 --------------------------------------QTAQVGIMLTIYAWVVALMSLP

2.A.1.2.18 --------------------------------------KTEDVGLMITIYAWIVAVASLI

2.A.1.2.25 --------------------------------------SIVSAGQLISVFALGYAVSGPL

2.A.1.2.26 --------------------------------------SEGVAGQSVTVTAFVAMFASLF

2.A.1.2.11 PFRVTW---TNGTIPPPVTEASSVPKNNCLQGIEFLEEENVRIGILFASKALMQLLVNPF

2.A.1.2.12 PSGIAWMNDTASTIPPPATEAISAHKNNCLQGTGFLEEEITRVGVLFASKAVMQLLVNPF

2.A.1.2.29 LTLHQ-------TATQHMVTNASAVPSDCPSEDKDLLNENVQVGLLFASKATVQLITNPF

```
2.A.1.2.28    SAL---------------------------RPRYPTESEDVKIGVLFASKAILQLLVNPL
2.A.1.2.30    --------------------------------------TFLMNGLIQGVKGLLSFLSAPL
2.A.1.2.9     --------------------------------------REGAVQSVMGAYLLTYGVSQLF
2.A.1.2.22    --------------------------------------SEAQLHIAFSVYLAGMAAAMLF
2.A.1.2.32    --------------------------------------TVGTAGTLTSAFATGMIVGAPL
2.A.1.2.21    --------------------------------------AAVMVGIALGLRQFIQQGLGIF
2.A.1.2.37    -------------------------------------SVVIYNTLSLILPMLANILLTPI
2.A.1.2.5     --------------------------------------GSAITGILLALSAVATFVAGIL
2.A.1.1.1     -I------------------------------------TSHTQEWVVSSMMFGAAVGAVG
2.A.1.1.2     -L------------------------------------TSRLQEWVVSSMMLGAAIGALF
2.A.1.1.26    -L------------------------------------NAFTEGLVTSSLLFGAALGAVF
2.A.1.1.53    -L------------------------------------TAFTEGVVTSSLLFGAAAGAMF
2.A.1.1.55    -L------------------------------------SPFMEGLVISSIMIGGVVGVGI
2.A.1.1.25    -L------------------------------------DALWQELLVSSTVGAAAVSALA
2.A.1.1.63    -R------------------------------------NTWLQEMIVSMAVAGAIVGAAI
2.A.1.1.66    -Q------------------------------------SSFLQETIVSMALVGAMIGAAA
2.A.1.1.32    -T------------------------------------DSLLTGLSVSLALLGSALGAFG
2.A.1.1.35    -V------------------------------------GSAVLAQVIAVALIGCAIGAAT
2.A.1.1.65    -I------------------------------------GNYALGLAVASALLGAAAGALS
2.A.1.1.6     -L------------------------------------SNVRTGLIVAIFNIGCAFGGII
2.A.1.1.67    -L------------------------------------SDVRVGLMISIFNVGCAVGGIF
2.A.1.1.36    -F------------------------------------SNVRNGLIVGLLCIGTMIGALV
2.A.1.1.58    -F------------------------------------SNVRSGLIVGLLCIGTMIGALV
2.A.1.1.19    -F------------------------------------TTSQIAILVSFLSLGTFFGALI
2.A.1.1.8     -L------------------------------------TYGEKEIVTAATSLGALITSIF
2.A.1.1.21    -L------------------------------------SSARQGLLTGMVNVGSLFGCII
```

```
2.A.1.1.23    -Y------------------------------------SAWRQALLTGTINAGCLFGAML
2.A.1.1.42    -L------------------------------------SVSQTGFITSSVLIGSCAGALS
2.A.1.1.33    -L------------------------------------TSDEDSLISSLMPLGAVGGSIL
2.A.1.1.18    -I------------------------------------SASWQIGLTLCYMAGEIVGLQL
2.A.1.1.20    -E------------------------------------HSWQYALIVAIAIAGAFVGAFI
2.A.1.1.14    -Y------------------------------------DNAKLQLFVSSLFLAGLVSCLF
2.A.1.1.50    -Y------------------------------------DNQGLQLFTSSLYLAGLTATFF
2.A.1.1.60    -Y------------------------------------DSPTLTMFTSSLYLAALISSLV
2.A.1.1.61    -Y------------------------------------DNELLTLFTSSLYLAALFASFL
2.A.1.1.56    -Y------------------------------------DNQFLQLFTSSLYLAALVASFV
2.A.1.1.37    -L------------------------------------MLLLWSCTVSMFPLGGLLGSLL
2.A.1.1.47    -T------------------------------------LTLLWSVTVSIFAIGGLVGTLM
2.A.1.1.52    -D------------------------------------SSFFYTHVSTIFVVAAAIGAFS
2.A.1.1.51    -F------------------------------------TADESSLIVSILSVGTFFGALC
2.A.1.1.57    -L------------------------------------PSWKKSLITSILSAGTFFGALI
2.A.1.1.28    -T------------------------------------LTTLWSLSVAIFSVGGMIGSFS
2.A.1.1.29    QL------------------------------------ITMLWSLSVSSFAVGGMTASFF
2.A.1.1.41    -L------------------------------------GSWQQGWVVSAVLLGAILGAAI
2.A.1.1.54    -L------------------------------------NVLQLGVVISSLVFAAAFGALF
2.A.1.1.24    -N------------------------------------NTIQSSFLLASVFIGAVLGCGF
2.A.1.1.43    -M------------------------------------ENLLWGFAVGSYAIGGLIGGIS
2.A.1.1.3     -A------------------------------------ANSLLGFCVASALIGCIIGGAL
2.A.1.1.4     -A------------------------------------AASLSGMVVVAVLVGCVTGSLL
2.A.1.1.46    -L------------------------------------GDNAASWFGAVVTLGAAAGGIL
2.A.1.1.59    -L------------------------------------SCLEQEFLVGSLLLGALLASLV
2.A.1.1.17    GY------------------------------------SSLYSGIFASAMIVGSMVGSII
```

2.A.1.2.1	LGPLSD--IGGRKPVYFCSI-FVYTVFNISCALP---R-----N---------------I
2.A.1.2.2	WSPLSENSRIGRTPLYIITL-FIFFILQIPTALS---N-----H---------------I
2.A.1.2.6	FSPMSENAIFGRTSIYIITL-FLFVILQIPTALV---N-----N---------------I
2.A.1.2.17	FSPLSETARYGRLNLYMVTL-FFFMIFQVGCATV---H-----N---------------I
2.A.1.2.35	LSPLSEIPQVGRQKIYVLSL-LVFVCLQIPTALG---S-----S---------------L
2.A.1.2.16	YAPLSE--LYGRKGVLVLSA-FGFALFQFAVATA---E-----N---------------L
2.A.1.2.40	WGALAD--FGGRKFLYMVSL-SLMLIVNILLAAV---PV----N---------------I
2.A.1.2.31	MGGFAD--SLGRRPVVLVAI-VIYFGACIGLACA---Q-----T---------------Y
2.A.1.2.3	FGPLSD--RLGRRPVLLGGG-LAYVVASMGLALT---S-----S---------------A
2.A.1.2.4	LGALSD--RFGRRPVLLVSL-AGAAVDYAIMATA---P-----F---------------L
2.A.1.2.38	LGALSD--RFGRRPVLIISI-AGATADYLLMAAA---P-----S---------------L
2.A.1.2.39	LGALSD--RFGRRPVLLISL-AGAAADYLLMAFA---P-----T---------------L
2.A.1.2.41	LGRLSD--RFGRRPVIIVAA-FGAAFSMALFGFG---G-----A---------------L
2.A.1.2.8	AGRWVD--RFGRKKMIILGL-LIFSLSELIFGLG---T-----H---------------V
2.A.1.2.10	GGTLAD--KLGKKLIICIGL-ILFSVSEFMFAVG---H-----N---------------F
2.A.1.2.24	SGSLID--KIGSRRVYLTGL-LTVAITTGLVALA---Q-----E---------------Y
2.A.1.2.20	WGGLAD--RKGRKLMLLRSA-LGMGIVMVLMGLA---Q-----N---------------I
2.A.1.2.34	WGILAD--KYGRKPMMIRAG-LAMTITMGGLAFV---P-----N---------------I
2.A.1.2.7	YGPMAD--SFGRKPVVLGGT-LVFAAAAVACALA---N-----T---------------I
2.A.1.2.27	HGTLSD--SLGRRRVVLGAL-GFYVWGALLATAA---P-----G---------------F
2.A.1.2.19	LGPLSD--RIGRRPVMLAGV-VWFIVTCLAILLA---Q-----N---------------I
2.A.1.2.14	IALFSS--RYSLKHILLFLV-ALCVIGNAMFTLS---S-----S---------------Y
2.A.1.2.15	FMLMTS--QVERRKLLICLF-VVFIASHVLSFLS---W-----S---------------F
2.A.1.2.18	CMLLTS--GIERRKLLIGLF-SLFILSHLLSAVA---W-----N---------------F

2.A.1.2.25	LLALTA--KIERKRLYLIAL-FVFFLSNLVAYFS---P-----N---------------F
2.A.1.2.26	ITQTIQ--ATDRRYVVILFA-VLLTLSCLLVSFA---N-----S---------------F
2.A.1.2.11	VGPLTN--RIGYHIPMFVGF-MIMFLSTLMFAFS---G-----T---------------Y
2.A.1.2.12	VGPLTN--RIGYHIPMFAGF-VIMFLSTVMFAFS---G-----T---------------Y
2.A.1.2.29	IGLLTN--RIGYPIPIFAGF-CIMFVSTIMFAFS---S-----S---------------Y
2.A.1.2.28	SGPFID--RMSYDVPLLIGL-GVMFASTVLFAFA---E-----D---------------Y
2.A.1.2.30	IGALSD--VWGRKPFLLGTV-FFTCFPIPLMRIS---P----------------------
2.A.1.2.9	YGPISD--RVGRRPVILVGM-SIFMLATLVAVTT---S-----S---------------L
2.A.1.2.22	AGKVAD--RSGRKPVAIPGA-ALFIIASVFCSLA---E-----T---------------S
2.A.1.2.32	VAALAR--TWPRRSSLLGFI-LAFAAAHAVGAGT---T-----S---------------F
2.A.1.2.21	GGAIAD--RFGAKPMIVTGM-LMRAAGFATMGIA---H-----E---------------P
2.A.1.2.37	WGLAAD--RYGYKPMLMRAS-WALVLTQASMIVV---N-----S---------------V
2.A.1.2.5	AGFFAD--RNGRKPVMVFGT-IIQLLGAALAIAS---NLPGHVN---------------P
2.A.1.1.1	SGWLSF--KLGRKKSLMIGA-ILFVAGSLFSAAA---P-----N---------------V
2.A.1.1.2	NGWLSF--RLGRKYSLMAGA-ILFVLGSIGSAFA---T-----S---------------V
2.A.1.1.26	GGRMSD--FNGRRKNILFLA-VIFFISTIGCTFA---P-----N---------------V
2.A.1.1.53	FGRISD--NWGRRKTIISLA-VAFFVGTMICVFA---P-----S---------------F
2.A.1.1.55	SGFLSD--RFGRRKILMTAA-LLFAISAIVSALS---Q-----D---------------V
2.A.1.1.25	GGALNG--VFGRRAAILLAS-ALFTAGSAVLAAA---N-----N---------------K
2.A.1.1.63	GGWAND--KLGRRSAILMAD-FLFLLGAIIMAAA---P-----N---------------P
2.A.1.1.66	GGWIND--YYGRKKATLFAD-VVFAAGAIVMAAA---P-----D---------------P
2.A.1.1.32	AGPIAD--RHGRIKTMILAA-VLFTLSSIGSGLP---F-----T---------------I
2.A.1.1.35	AGRIAD--RIGRIRCMQIAA-VLFTVSAVGSALP---F-----A---------------L
2.A.1.1.65	AGRIAD--RIGRIAVMKIAA-VLFFISAFGTGFA---P-----E---------------T
2.A.1.1.6	LSKGGD--MYGRKKGLSIVV-SVYIVGIIIQIASI--N-----K---------------W
2.A.1.1.67	LCKVAD--VWGRRIGLMFSM-AVYVVGIIIQISSS--T-----K---------------W

2.A.1.1.36 AAPIAD--RIGRKLSMSFWS-IIHIVGIIIQIATD--S-----N---------------W

2.A.1.1.58 AAPIAD--RMGRKLSICLWS-VIHIVGIIIQIATD--S-----N---------------W

2.A.1.1.19 APYISD--SYGRKPTIMFST-AVIFSIGNSLQVAS--G-----G---------------L

2.A.1.1.8 AGTAAD--IFGRKRCLMGSN-LMFVIGAILQVSAH--T-----F---------------W

2.A.1.1.21 SSPIAD--RFGKRLSIIGFC-AVYIIGIIVQVTAV--P-----S---------------W

2.A.1.1.23 SSPFTE--RIGKKYSICFFS-GVYIIAELLLVTAV--P-----S---------------W

2.A.1.1.42 IGALSD--RFGRKKLLIVSA-LLFLLGSGLCASS---T-----G---------------F

2.A.1.1.33 LTPLSE--YFGRKVALVISC-IFYTIGGILCAAA---Q-----D---------------V

2.A.1.1.18 TGPSVD--LVGNRYTLIIAL-FFLAAFTFILYFC---N-----S---------------L

2.A.1.1.20 SGFISA--AFGRRPCIAVAD-ALFVIGSVLMGAA---P-----N---------------V

2.A.1.1.14 ASWITR--NWGRKVTMGIGG-AFFVAGGLVNAFA---Q-----D---------------M

2.A.1.1.50 ASYTTR--TLGRRLTMLIAG-VFFIIGVALNAGA---Q-----D---------------L

2.A.1.1.60 ASTVTR--KFGRRLSMLFGG-ILFCAGALINGFA---K-----H---------------V

2.A.1.1.61 ASTITR--LFGRKVSMVIGS-LAFLSGALLNGLA---I-----N---------------L

2.A.1.1.56 ASATCS--KLGRRPTMQFAS-IFFLIGVGLTAGA---V-----N---------------L

2.A.1.1.37 VGLLVD--SCGRKGTLLINN-IFAIIPAILMGVSKVAK-----A---------------F

2.A.1.1.47 VKMIGK--FLGRKSTLLVNN-GFAISAALLMACSLRAG-----T---------------F

2.A.1.1.52 CGWVAD--GLGRRNGLILNN-VIGIIGGVIVGPCVLVK-----Q---------------P

2.A.1.1.51 APFLND--TLGRRWCLILSALIVFNIGAILQVIS---T-----A---------------I

2.A.1.1.57 AGDLAD--WFGRRTTIVSGC-VVFIVGVILQTAS---T-----S---------------L

2.A.1.1.28 VGLFVN--RFGRRNSMLMMN-LLAFVSAVLMGFSKLGK-----S---------------F

2.A.1.1.29 GGWLGD--TLGRIKAMLVAN-ILSLVGALLMGFSKLGP-----S---------------H

2.A.1.1.41 IGPSSD--RFGRRKLLLLSA-IIFFVGALGSAFSPEFW-----T----------------

2.A.1.1.54 AGRISD--EIGRRKAIITLS-VLFFLGSILVVFSPAGE-----LGQ--------FYGPGF

2.A.1.1.24 SGYLVQ---FGRRLSLLIIY-NFFFLVSILTSIT---H-----H---------------F

2.A.1.1.43 AGYLQT--RFGRIRTLVGNN-FSFILGALILGSA---V-----N---------------P

```
2.A.1.1.3     GGYCSN--RFGRRDSLKIAA-VLFFISGVGSAWPELGF-----TSINPDNTVPVYLAGYV
2.A.1.1.4     SGWIGI--RFGRRGGLLMSS-ICFVAAGFGAALTEKLF-----GTG----------GSAL
2.A.1.1.46    GGWLLD--RSGRKLSLLLCT-VPFVTGFAVITAARD-----------------------V
2.A.1.1.59    GGFLID--CYGRKQAILGSN-LVLLAGSLTLGLA---G-----S---------------L
2.A.1.1.17    AGKCIT--MFGLKKSFIIVG-VMSVVASALNHISVATN-----E---------------F
```

```
2.A.1.2.1     VQMIISHFIIGVAGSTALTNVAGGIPDLFPE-DTAGVPMSLFVWACAGGAIGAPMATGVD
2.A.1.2.2     AGLSVLRVIAGFFAAPALSTGGASYGDFIAM-HYYSIALGVWSIFAVAGPSIGPLIGAAV
2.A.1.2.6     AGLCILRFLGGFFASPCLATGGASVADVVKF-WNLPVGLAAWSLGAVCGPSFGPFFGSIL
2.A.1.2.17    GGLIVMRFISGILCSPSLATGGGTVADIISP-EMVPLVLGMWSAGAVAAPVLAPLLGAAM
2.A.1.2.35    GVLLPMRFLAGFFGSPALATGGATLADIWQP-WLLPYFMCFWAIGAIGGPVLGPLLGGAM
2.A.1.2.16    QTIFICRFFGGFIGAAPMAVVPAAFADMFDT-NVRGKAIALFSLGVFVGPILSPVMGSYI
2.A.1.2.40    AALFVLRIFQAFASSSVISLGAGTVTDVVPP-KHRGKAIAYFMMGPNMGPIIAPIVAGLI
2.A.1.2.31    AQIIVLRCLQAAGISPVIAINSGIMGDVTTR-AERGGYVGYVAGFQVLGSAFGALIGAGL
2.A.1.2.3     EVFLGLRILQACGASACLVSTFATVRDIYAGREESNVIYGILGSMLAMVPAVGPLLGALV
2.A.1.2.4     WVLYIGRIVAGITGATG-AVAGAYIADITDG-DERARHFGFMSACFGFGMVAGPVLGGLM
2.A.1.2.38    LWLYIGRIFAGITGANM-AVATAYVSDITPA-HERAKRFGLLGAVFGIGFIAGPVIGGVL
2.A.1.2.39    AWLYLGRLLAGITGANM-AVATAYVTDITPV-GQRARRFGLVGAVFGVGFIVGPLLGGSL
2.A.1.2.41    WVLVLARVIQGLTAGDL-PALFAYLADITPP-EQRAKRFGLLGALSGIGTMIGPAIGGLL
2.A.1.2.8     SIFYFSRILGGVSAAFIMPAVTAYVADITTL-KERSKAMGYVSAAISTGFIIGPGAGGFI
2.A.1.2.10    SVLMLSRVIGGMSAGMVMPGVTGLIADISPS-HQKAKNFGYMSAIINSGFILGPGIGGFM
2.A.1.2.24    WQILLLRGIAGIGSTMFTVSAMGLIVKMAPV-EIRGRCSSVYASSFLFGNIIGPVVGAAM
2.A.1.2.20    WQFLILRALLGLLGGFV-PNANALIATQVPR-NKSGWALGTLSTGGVSGALLGPMAGGLL
2.A.1.2.34    YWLIFLRLLNGVFAGFV-PNATALIASQVPK-EKSGSALGTLSTGVVAGTLTGPFIGGFI
2.A.1.2.7     DQLIVMRFFHGLAAAAASVVINALMRDIYPK-EEFSRMMSFVMLVTTIAPLMAPIVGGWV
```

2.A.1.2.27	GWLLAARALQGLSAGAGVVVGQAIIRDCYQG-AAAQRSMSYLILVFNLSPALAPVIGGQL
2.A.1.2.19	EQFTLLRFLQGISLCFIGAVGYAAIQESFEE-AVCIKITALMANVALIAPLLGPLVGAAW
2.A.1.2.14	LMLAIGRLVSGFPHGAFFGVGAIVLSKIIKP-GKVTAAVAGMVSGMTVANLLGIPLGTYL
2.A.1.2.15	TVLVISRIGVAFAHAIFWSITASLAIRMAPA-GKRAQALSLIATGTALAMVLGLPLGRIV
2.A.1.2.18	TVLVISRAGVALAHSVFWSITASLAIRMAPP-GKRAQALGLIATGSSLAMVLGLPLGRVI
2.A.1.2.25	ATLMVSRVLAAMSTGLIVVLSLTIAPKIVAP-EYRARAIGIIFMGFSSAIALGVPLGILI
2.A.1.2.26	SLLLIGRACLGLALGGFWAMSASLTMRLVPP-RTVPKALSVIFGAVSIALVIAAPLGSFL
2.A.1.2.11	ALLFVARTLQGIGSSFSSVAGLGMLASVYTDNYERGRAMGIALGGLALGLLVGAPFGSVM
2.A.1.2.12	TLLFVARTLQGIGSSFSSVAGLGMLASVYTDDHERGRAMGTALGGLALGLLVGAPFGSVM
2.A.1.2.29	AFLLIARSLQGIGSSCSSVAGMGMLASVYTDDEERGNVMGIALGGLAMGVLVGPPFGSVL
2.A.1.2.28	ATLFAARSLQGLGSAFADTSGIAMIADKYPEEPERSRALGVALAFISFGSLVAPPFGGIL
2.A.1.2.30	WWYFAMISVSGVFSVTF-SVIFAYVADVTQE-HERSTAYGWVSATFAASLVSSPAIGAYL
2.A.1.2.9	TVLIAASAMQGMGTGVGGVMARTLPRDLYER-TQLRHANSLLNMGILVSPLLAPLIGGLL
2.A.1.2.22	TLFLAGRFLQGLGAGCCYVVAFAILRDTLDD-RRRAKVLSLLNGITCIIPVLAPVLGHLI
2.A.1.2.32	PVLVACRVVAALANAGFLAVALTTAAALVPA-DKQGRALAVLLSGTTVATVAGVPGGSLL
2.A.1.2.21	WLLWFSCLLSGLGGTLFDPPRSALVVKLIRP-QQRGRFFSLLMMQDSAGAVIGALLGSWL
2.A.1.2.37	FWILIIRLLQGAFAGFLVAMQTYALS-ITEW-QNKSTQLSRLQSSKAIATSTAGFLGGLA
2.A.1.2.5	WSTFIAFLLISFGYNFVITAGNAMIIDASNA-ENRKVVFMLDYWAQNLSVILGAALGAWL
2.A.1.1.1	EVLILSRVLLGLAVGVASYTAPLYLSEIAPE-KIRGSMISMYQLMITIGILGAYLSDTAF
2.A.1.1.2	EMLIAARVVLGIAVGIASYTAPLYLSEMASE-NVRGKMISMYQLMVTLGIVLAFLSDTAF
2.A.1.1.26	TVMIISRFVLGIAVGGASVTVPAYLAEMSPV-ESRGRMVTQNELMIVSGQLLAFVFNAIL
2.A.1.1.53	AVMVVGRVLLGLAVGGASTVVPVYLAELAPF-EIRGSLAGRNELMIVVGQLAAFVINAII
2.A.1.1.55	STLIIARIIGGLGIGMGSSLSVTYITEAAPP-AIRGSLSSLYQLFTILGISATYFINLAV
2.A.1.1.25	ETLLAGRLVVGLGIGIASMTVPVYIAEVSPP-NLRGRLVTINTLFITGGQFFASVVDGAF
2.A.1.1.63	SLLVVGRVFVGLGVGMASMTAPLYISEASPA-KIRGALVSTNGFLITGGQFLSYLINLAF
2.A.1.1.66	YVLISGRLLVGLGVGVASVTAPVYIAEASPS-EVRGGLVSTNVLMITGGQFLSYLVNSAF

2.A.1.1.32	WDFIFWRVLGGIGVGAASVIAPAYIAEVSPA-HLRGRLGSLQQLAIVSGIFIALLSNWFI
2.A.1.1.35	WDLAMWRIIGGFAIGMASVIGPAYIAEVSPP-AYRGRLGSFQQAAIVIGIAVSQLVNWGL
2.A.1.1.65	VTLVVFRIVGGIGVGVASVIAPAYIAETSPP-GIRGRLGSLQQLAIVLGIFTSFVVNWLL
2.A.1.1.6	YQYFIGRIISGLGVGGIAVLCPMLISEIAPK-HLRGTLVSCYQLMITAGIFLGYCTNYGT
2.A.1.1.67	YQFFIGRLIAGLAVGTVSVVSPLFISEVSPK-QIRGTLVCCFQLCITLGIFLGYCTTYGT
2.A.1.1.36	VQIAMGRWVAGLGVGALSSVVPMYQSEAAPR-QVRGAMISAFQLFVAFGIFISYIINYGT
2.A.1.1.58	VQVAMGRWVAGLGVGALSSIVPMYQSESAPR-QVRGAMVSAFQLFVAFGIFISYIINFGT
2.A.1.1.19	VLLIVGRVISGIGIGIISAVVPLYQAEAAQK-NLRGAIISSYQWAITIGLLVSSAVSQG-
2.A.1.1.8	-QMAVGRLIMGFGVGIGSLIAPLFISEIAPK-MIRGRLTVINSLWLTGGQLVAYGCGAG-
2.A.1.1.21	VQIMVAKIWTGIGIGALSVLAPGYQSETAPP-SIRGTVVVTYQLFVTGGIFIAACINMGT
2.A.1.1.23	IQVLVGKILAGVGIGALSVLSPGYQSEVAPP-QIRGAVVATYQIFSTGAALVAACINMGT
2.A.1.1.42	AMMVCARIILGLAVGAASALTPAYLAELAPK-ERRGSLSTLFQLMVTFGILLAYASNLGF
2.A.1.1.33	HTMYAGRFLIGVGVGIEGGGVGVYIAESVPS-TVRGSLVSLYQFNIALGELVGYVIGVI-
2.A.1.1.18	GMIAVGQALCGMPWGCFQCLTVSYASEICPL-ALRYYLTTYSNLCWLFGQLFAAGIMKNS
2.A.1.1.20	EVVLVSRVIVGLAIGISSATIPVYLAEVTSP-KHRGATIVLNNLFLTGGQFVAAGFTAIM
2.A.1.1.14	AMLIVGRVLLGFGVGLGSQVVPQYLSEVAPF-SHRGMLNIGYQLFVTIGILIAGLVNYAV
2.A.1.1.50	AMLIAGRILLGCGVGFANQAVPLFLSEIAPT-RIRGGLNILFQLNVTIGILFANLVNYGT
2.A.1.1.60	WMLIVGRILLGFGIGFANQAVPLYLSEMAPY-KYRGALNIGFQLSITIGILVAEVLNYFF
2.A.1.1.61	EMLIIGRLFLGVGVGFANQSVPLYLSEMAPA-KIRGALNIGFQLAITIGILAANIVNYVT
2.A.1.1.56	VMLIIGRLFLGFGVGFGNQAVPLFLSEIAPA-QLRGGLNIVFQLMVTIGILIANIVNYFT
2.A.1.1.37	ELIVFSRVVLGVCAGISYSALPMYLGELAPK-NLRGMVGTMTEVFVIVGVFLAQIFSLQA
2.A.1.1.47	EMLIVGRFIMGVDGGIALSALPMYLNEISPK-EIRGSLGQVTAIFICIGVFSGQLLGLPE
2.A.1.1.52	ALLYVGRFVIGINSGITIGIASLYLTEVAPR-DLRGGIGACHQLAVTVGIAFSYFITFTF
2.A.1.1.51	PLLCAGRVIAGFGVGLISATIPLYQSETAPK-WIRGAIVSCYQWAITIGLFLASCVNKGT
2.A.1.1.57	GLLVAGRLVAGFGVGFVSAIIILYMSEIAPR-KVRGAIVSGYQFCITIGLMLASCVDYGT
2.A.1.1.28	EMLILGRFIIGVYCGLTTGFVPMYVGEVSPT-AFRGALGTLHQLGIVVGILIAQVFGLDS

2.A.1.1.29	ILIIAGRSISGLYCGLISGLVPMYIGEIAPT-ALRGALGTFHQLAIVTGILISQIIGLEF
2.A.1.1.41	--LIISRIILGMAVGAASALIPTYLAELAPS-DKRGTVSSLFQLMVMTGILLAYITNYSF
2.A.1.1.54	ATLVTGRIMLGLAVGGASTVVPVYLAELAPL-EIRGSLTGRNELAIVTGQLLAFVINALI
2.A.1.1.24	HTILFARLLSGFGIGLVTVSVPMYISEMTHK-DKKGAYGVMHQLFITFGIFVAVMLGLAM
2.A.1.1.43	GMFIIGRILTGVGSGISTVTVPTYLGEIATV-KARGALGTIYQLFLVIGILFTQIIGLLL
2.A.1.1.3	PEFVIYRIIGGIGVGLASMLSPMYIAELAPA-HIRGKLVSFNQFAIIFGQLLVYCVNYFI
2.A.1.1.4	QIFCFFRFLAGLGIGVVSTLTPTYIAEIRPP-DKRGQMVSGQQMAIVTGALTGYIFTWLL
2.A.1.1.46	WMLLGGRLLTGLACGVASLVAPVYISEIAYP-AVRGLLGSCVQLMVVTGILLAYVAGWVL
2.A.1.1.59	AWLVLGRAVVGFAISLSSMACCIYVSELVGP-RQRGVLVSLYEAGITVGILLSYALNYAL
2.A.1.1.17	WVLCAGRVLMGIGLGVVCVICPMYVNENAHP-KLSKVDGVLFQVFITFGIMLAAMLGLIL

2.A.1.2.1	IN---AKY--------------GWRWLYYINIIVGGFFLIVILIIP-ETLPIKVITRYEN
2.A.1.2.2	INRSHDAD--------------GWRWSFWFMAILSGVCFIVLSFSL-PETYGKTLLRRKA
2.A.1.2.6	T----VKA--------------SWRWTFWFMCIISGFSFVMLCFTL-PETFGKTLLYRKA
2.A.1.2.17	V----DAK--------------NWRFIFWLLMWLSAATFILLAFFF-PETQHHNILYRRA
2.A.1.2.35	V----VAE--------------SWRWQFWLLMMISGFALIVIFFFM-PETSEWHILYKRA
2.A.1.2.16	A----QRT--------------TWRWLEYVVGCFASAVFVAIVLFF-EETHHPTILVNKA
2.A.1.2.40	LM---KGN--------------YWRWLFGFTSIMTGIALILVTALL-PETLRCIVGNGDP
2.A.1.2.31	S----SRW--------------GWRAIFWFLAIGSGICFLASFLIL-PETKRNISGNGSV
2.A.1.2.3	D----MWL--------------GWRAIFAFLGLGMIAASAAAWRFW-PET-----R---V
2.A.1.2.4	G----G-F--------------SPHAPFFAAAALNGLNFLTGCFLL-PESHKGERR--PL
2.A.1.2.38	G-----EW--------------NLHAPFFAAAFMNGINLIMTAVLL-KESKHSNKMTEKV
2.A.1.2.39	G-----EW--------------HLHAPFLAAAMMNALNLVMAFFLL-PESRKSRPRAAEK
2.A.1.2.41	A----A-I--------------SIQLPVFLTAAVALTIAILSIFLL-PESLKPGNRITAI
2.A.1.2.8	A-----GF--------------GIRMPFFFASAIALIAAVTSVFIL-KESLSIEERHQLS

2.A.1.2.10 A-----EV--------------SHRMPFYFAGALGILAFIMSIVLI-HDPKKSTTSGFQK

2.A.1.2.24 S----G-L--------------GMRWPFAIYGASVGLAALVVWWRM-PKTNDSLRKADSN

2.A.1.2.20 A----DSY--------------GLRPVFFITASVLILCFFVTLFCI-REKFQPVSKKEML

2.A.1.2.34 A----ELF--------------GIRTVFLLVGSFLFLAAILTICFI-KEDFQPVAKEKAI

2.A.1.2.7 L----VWL--------------SWHYIFWILALAAILASAMIFFLI-KETLPPERRQPFH

2.A.1.2.27 A----VHH--------------GWRSIFFMLGLLAATALTLCAWRL-PETLAPAKRQTLS

2.A.1.2.19 I----HVL--------------PWEGMFVLFAALAAISFFGLQRAM-PET-ATRIGEKLS

2.A.1.2.14 S----QEF--------------SWRYTFLLIAVFNIAVMASVYFWV-PDI-------RDE

2.A.1.2.15 G----QYF--------------GWRMTFFAIGIGALITLLCLIKLL-PLL-------PSE

2.A.1.2.18 G----QYL--------------GWRVTFLTIAAGATVAMILLARLL-PLL-------PSE

2.A.1.2.25 S----DSF--------------GWRILFLGIGLLALISMLIISIFF-ERI-------PAE

2.A.1.2.26 G----ELI--------------GWRNVFNAAAVMGVLCIFWIIKSL-PSL-------PGE

2.A.1.2.11 Y----EFV--------------GKSSPFLILAFLALLDGALQLCIL-WP----SKVSPES

2.A.1.2.12 Y----EFV--------------GKSAPFLILAFLALLDGALQLCIL-QP----SKVSPES

2.A.1.2.29 Y----EFV--------------GKTAPFLVLAALVLLDGAIQLFVL-QP----SRVQPES

2.A.1.2.28 Y----EFA--------------GKRVPFLVLAAVSLFDALLLLAVA-KPFSAAARARANL

2.A.1.2.30 S----ASY--------------GDSLVVLVATVVALLDICFILVAV-PESLPEKMRPVSW

2.A.1.2.9 D----TMW--------------NWRACYLFLLVLCAGVTFSMARWM-PETRPVD-APRTR

2.A.1.2.22 M----LKF--------------PWQSLFWAMAMMGIAVLMLSLFIL-KETRPAAPAASDK

2.A.1.2.32 G----TWL--------------GWRATFWAVAVCCLPAAFGVLKAI-PAGRATAAATGGP

2.A.1.2.21 L----Q-Y--------------DFRLVCATGAVLFVLCAAFNAWLL-PAWKLSTVRTPVR

2.A.1.2.37 L----SFT--------------NYQGLFGLATLICLGTTVAMHYKL-PSPPKHQ------

2.A.1.2.5 F-----RP--------------AFEALLVILLLTVLVSFFLTTFVM-TETFKPTVKVDEK

2.A.1.1.1 S----YT--------------GAWRWMLGVIIIPAILLLIGVFFL--PDSPRW-FAAKRR

2.A.1.1.2 S----YS--------------GNWRAMLGVLALPAVLLIILVVFL--PNSPRW-LAEKGR

2.A.1.1.26 G-------TT------MGDNSHVWRFMLVIASLPALFLFFGMIRM--PESPRW-LVSKGR

2.A.1.1.53	G-------NV------FGHHDGVWRYMLAIAAIPAIALFFGMLRV--PESPRW-LVERGR
2.A.1.1.55	Q----RSGTY------EWGVHTGWRWMLAYGMVPSVIFFLVLLVV--PESPRW-LAKAGK
2.A.1.1.25	S----YL------------QKDGWRYMLGLAXVPAVIQFFGFLFL--PESPRW-LIQKGQ
2.A.1.1.63	T----DV-------------TGTWRWMLGIAGIPALLQFVLMFTL--PESPRW-LYRKGR
2.A.1.1.66	T----QV-------------PGTWRWMLGVSGVPAVIQFILMLFM--PESPRW-LFMKNR
2.A.1.1.32	A----LMAGGS-AQNPWLFGAAAWRWMFWTELIPALLYGVCAFLI--PESPRY-LVAQGQ
2.A.1.1.35	L----NAAGGD--QRGELMGLEAWQVMLGVMVIPAVLYGLLSFAI--PESPRF-LISVGK
2.A.1.1.65	Q----WAAGGP--NEVLAMGLDAWRWMFLAMAVPAVLYGALAFTI--PESPRY-LVATHK
2.A.1.1.6	K----SYS-----------NSVQWRVPLGLCFAWSLFMIGALTLV--PESPRY-LCEVNK
2.A.1.1.67	K----TYT-----------DSRQWRIPLGLCFAWAILLVVGMLNM--PESPRY-LVEKHR
2.A.1.1.36	E----SIQ-----------STASWRITMGIGFAWPLILGLGALFL--PESPRY-AYRLGR
2.A.1.1.58	E----RIQ-----------STASWRITMGIGFAWPLILAVGSLFL--PESPRF-AYRQGR
2.A.1.1.19	-----THS--------KN-GPSSYRIPIGLQYVWSSILAVGMIF-L-PESPRY-YVLKDE
2.A.1.1.8	-----LNY--------VN-N--GWRILVGLSLIPTAVQFTCLCF-L-PDTPRY-YVMKGD
2.A.1.1.21	H----KLH-----------KTAQWRVSIGINLLWGIITMIGILFL--PESPRY-LIQVGK
2.A.1.1.23	H----KLR-----------KTASWRTSFGINMLWGILLMVGVLFL--PESPRY-LIYKGR
2.A.1.1.42	L----NHN--------LF-GIRDWRWMLGSALVPAALLLLGGLL-L-PESPRY-LVNKGD
2.A.1.1.33	-------F--------FD-VKGGWRYMLGSSLVFSTILFVGLFF-L-PESPRW-LIHKGY
2.A.1.1.18	Q----KKY--------AD-SELGYKLPFALQWILPVPLALGIFF-A-PESPWW-LVKKGR
2.A.1.1.20	V----VF---------TS-KNIGWRVAIGIGALPAVVQAFCLLFFL-PESPRW-LLSKGH
2.A.1.1.14	R----DWE-----------N--GWRLSLGPAAAPGAILFLGSLVL--PESPNF-LVEKGK
2.A.1.1.50	A----KIK-----------GGWGWRLSLGLAGIPALLLTVGALLV--TETPNS-LVERGR
2.A.1.1.60	A----KIK-----------GGWGWRLSLGGAVVPALIITIGSLVL--PDTPNS-MIERGQ
2.A.1.1.61	P----KLQ-----------NGIGWRLSLGLAGVPAVMMLVGCFFL--PDTPNS-ILERGN
2.A.1.1.56	A----TVH-----------PY-GWRIALGGAGIPAVILLFGSLLI--IETPTS-LIERNK
2.A.1.1.37	I----LGN------------PAGWPVLLALTGVPALLQLLTLPFF--PESPRYSLIQKGD

```
2.A.1.1.47    L----LGR------------ESTWPYLFGVIIVPALVQLASLPFL--PESPRYLLFEKHD
2.A.1.1.52    L----LNT-----------LN-LWPLAVALGAVPAAISLVTLPFC--PESPRFLYMKKHK
2.A.1.1.51    E----HMT-----------NSGSYRIPLAIQCLWGLILGIGMIFL--PETPRF-WISKGN
2.A.1.1.57    E----NRL-----------DSGSYRIPIGLQLAWALILGGGLLCL--PESPRY-FVKKGD
2.A.1.1.28    I----MGN------------KDLWPLLLSIIFIPALLQCIVLPFC--PESPRFLLINRNE
2.A.1.1.29    I----LGN------------YDLWHILLGLSGVRAILQSLLLFFC--PESPRYLYIKLDE
2.A.1.1.41    S----GFY--------TG-----WRWMLGFAAIPAALLFLGGLIL--PESPRF-LVKSGH
2.A.1.1.54    A----VTL--------HGVIDGIWRIMFAVCALPAVALFLGMLRM--PESPRW-LVNQGR
2.A.1.1.24    G----EGPKADSTEPLTSFAKLWWRLMFLFPSVISLIGILALVVFFKEETPYF-LFEKGR
2.A.1.1.43    S----SVP--------------GWRILLALTAIPALIQLILLRFC--VETPRY-LISQNK
2.A.1.1.3     A----RSGDA------SWLNTDGWRYMFASECIPALLFLMLLYTV--PESPRW-LMSRGK
2.A.1.1.4     A----HFGSI------DWVNASGWCWSPASEGLIGIAFLLLLLTA--PDTPHW-LVMKGR
2.A.1.1.46    ----------------------EWRWLAVLGCVPPTLMLLLMCYM--PETPRF-LLTQHQ
2.A.1.1.59    A----GTP-------------WGWRHMFGWATAPAVLQSLSLLFL--PAGT--------D
2.A.1.1.17    D----KTVNYD----NDPDMAGRFHGFCAVSSVLSVAMFLVGMFL--RESTAT--FSQDD
```

```
2.A.1.2.1     AKGRIVEG--------------IPKNNLKEVLKKCKF-----------------------
2.A.1.2.2     ERLRKLTG--------------NNRIISEGELEDGHKTTSQV------------------
2.A.1.2.6     KRLRAITG--------------NDRITSEGEIENSKMTSHEL------------------
2.A.1.2.17    LKLRKETG--------------DDRYYTEQDKLDREVDARTF------------------
2.A.1.2.35    KRFRKITG--------------NENYRTEAELASSHLSVAQL------------------
2.A.1.2.16    KQMRKQSN--------------NWGIHAA--HEDVELSIKDI------------------
2.A.1.2.40    KWGDKKDE--------------RENNESPFFEGNKISHRRLFPDIGIRKPVNNDAFFQEN
2.A.1.2.31    TPKSYLNR--------------APILVLPTVRKSLHLDNPDY----------------ET
2.A.1.2.3     QRVAGLQW--------------SQ------------------------------------
```

2.A.1.2.4	RREALNPL--------------A-------------------------------------
2.A.1.2.38	QEQSILKK--------------L-------------------------------------
2.A.1.2.39	IRLNPFSS--------------L-------------------------------------
2.A.1.2.41	KLRDVQPF--------------A-------------------------------------
2.A.1.2.8	SHTKESNF--------------I-------------------------------------
2.A.1.2.10	LEPQLLTK--------------I-------------------------------------
2.A.1.2.24	SVPALRFA--------------E-------------------------------------
2.A.1.2.20	HMREVVTS--------------L-------------------------------------
2.A.1.2.34	PTKELFTS--------------V-------------------------------------
2.A.1.2.7	IRTTIGNF--------------A-------------------------------------
2.A.1.2.27	LRGLAAGY--------------R-------------------------------------
2.A.1.2.19	LKELGRDY--------------K-------------------------------------
2.A.1.2.14	AKGNLREQ--------------F-------------------------------------
2.A.1.2.15	HSGSLKSL--------------P-------------------------------------
2.A.1.2.18	HSGSLGSV--------------P-------------------------------------
2.A.1.2.25	KMIPFREQ--------------L-------------------------------------
2.A.1.2.26	PSHQKQNT--------------F-------------------------------------
2.A.1.2.11	AMGTSLLT--------------L-------------------------------------
2.A.1.2.12	AKGTPLFM--------------L-------------------------------------
2.A.1.2.29	QKGTPLTT--------------L-------------------------------------
2.A.1.2.28	PVGTPIHR--------------L-------------------------------------
2.A.1.2.30	GAQISWKQ--------------AD------------------------------------
2.A.1.2.9	LLTSYKTL--
2.A.1.2.22	PRENSESL--
2.A.1.2.32	PLRVELAA--
2.A.1.2.21	EGMTRVMR--

2.A.1.2.37 IKKKSQTL--------------N-------------------------------------

2.A.1.2.5 AENIFQAY--------------KTV-----------------------------------

2.A.1.1.1 FVDAERVLLRLRDTSAE-----AKRELDEIRESLQVKQSGWALFK---------------

2.A.1.1.2 HIEAEEVLRMLRDTSEK-----AREELNEIRESLKLKQGGWALFK---------------

2.A.1.1.26 KEDALRVLKKIRDEKR------AAAELQEIEFAFKKEDQLEKATF---------------

2.A.1.1.53 IDEARAVLETIRPLER------AHAEVADVEHLAREEHAVSEKSM---------------

2.A.1.1.55 TNEALKILTRINGETV------AKEELKNIENSLKIEQMGSLS-----------------

2.A.1.1.25 TQKARRILSQMRGNQT------IDEEYDSIKNNIEEE-EKEVGSAGP-------------

2.A.1.1.63 EEEAKAILRRIYSAED------VEQEIRALKDSVETEILEEGSSEKI-------------

2.A.1.1.66 KAEAIQVLARTYDISR------LEDEIDHLSAAEEE---EKQRKRTV-------------

2.A.1.1.32 GEKAAAILWKVEGGD-------VPSRIEEIQATVSLDHKPRFSDL---------------

2.A.1.1.35 RERAKKILEEVEGKDVD-----FDARVTEIEHAMHREEKSSFKDL---------------

2.A.1.1.65 IPEARRVLSMLLGQK-N-----LEITITRIRDTLEREDKPSWRDL---------------

2.A.1.1.6 VEDAKRSIAKSNKVSPEDPA--VQAELDLIMAGIEAEKLAGNASW---------------

2.A.1.1.67 IDEAKRSIARSNKIPEEDPF--VYTEVQLIQAGIEREALAGQASW---------------

2.A.1.1.36 IDEARKVMTKLYGVEVNHRV--VVQEMKDMKDKLEEERAAGVAPW---------------

2.A.1.1.58 IDEAREVMCKLYGVSPNHRV--IAQEMKDMKDKLDEEKAAGQAAW---------------

2.A.1.1.19 LNKAAKSLSFLRGLPIEDPR--LLEELVEIKATYDYEA--SFGPSTLL------------

2.A.1.1.8 LARATEVLKRSYTDTSEEII--ERKVEELVTLNQSIPG--KNVPEKVW------------

2.A.1.1.21 DEEAVRVLSESAELFPDSEE--VQNEYHRLKSSIDEEFAGGPCSW---------------

2.A.1.1.23 DEEALRIMCNMAELSPESEI--IQTNFNTIKSDIEIEMAGGKARW---------------

2.A.1.1.42 TRNAFKVLTLIRK-DVDQTQ--VQIELDEIKAVAAQDT--KGGVRELF------------

2.A.1.1.33 DVEAYKVWRRLRDTSDLGNK--REFLEMKHAAEQDRQL--KEQESRFK------------

2.A.1.1.18 FDEARRSLRRTLSGKGPEKEILVTLEVDKIKVTIDKEKRLTSKEGSYS------------

2.A.1.1.20 ADRAKAVADKF------------EVDLCEFQEGDELPSVRIDYRPLM-------------

2.A.1.1.14 TEKGREVLQKLCGTSE------VDAEFADIVAAVEIARPITMRQS---------------

2.A.1.1.50 LDEGKAVLRRIRGTDN------VEPEFADLLEASRLAKEV--KHP---------------

2.A.1.1.60 HEEAKTKLRRIRGVDD------VSQEFDDLVAASKESQSI--EHP---------------

2.A.1.1.61 KEKAKEMLQKIRGTME------VEHEFNELCNACEAAKKV--KHP---------------

2.A.1.1.56 NEEGKEALRKIRGVDD------INDEYESIVHACDIASQV--KDP---------------

2.A.1.1.37 EATARQALRRLRGHTD------MEAELEDMRAEARAERAEGHLSV---------------

2.A.1.1.47 EAGAMKAFQTFLGKAD------VSQELEEALAESRVQRNLRLVSV---------------

2.A.1.1.52 EAEARKAFLQLNVKEN------VDTFIGELREEIEVAKNQPVFKF---------------

2.A.1.1.51 QEKAAESLARLRKLPIDHPD--SLEELRDITAAYEFETVYGKSS----------------

2.A.1.1.57 LAKAAEVLARVRGQPQDSDY--IKDELAEIVANHEYEMQVIPEGGYFV-------SWMN-

2.A.1.1.28 ENRAKSVLKKLRGTAD------VTHDLQEMKEESRQMMREKKVTI---------------

2.A.1.1.29 EVKAKQSLKRLRGYDD------VTKDINEMRKEREEASSEQKVSI---------------

2.A.1.1.41 LDEARHVLDTMNKH---------DQVAVNKEINDIQESAKIVS-----------------

2.A.1.1.54 YDDARRVMETVRTP---------ERAKAEMDEIIAVHSENNAALPGVKQSSGQASGQVSS

2.A.1.1.24 IEESKNILKKIYETDN------VDEPLNAIKEAVEQNESAKKNS----------------

2.A.1.1.43 LDEAQQSLQLLRPGFD------VTNEYKEIYDGQQEAETVESRDPEKNPKTKDPKAISSG

2.A.1.1.3 QEQAEGILRKIMGNTL------ATQAVQEIKHSLDHG-RKTGGRL---------------

2.A.1.1.4 HSEASKILARLEPQAD------PNLTIQKIKAGFDKAMDKSSAGL---------------

2.A.1.1.46 YQEAMAALRFLWGSEE------GW---EEPPVGAEHQGFQLALLR---------------

2.A.1.1.59 ETATHKDLIPLQG-----------GEAPKLGPGRPRYSFLDLFRA---------------

2.A.1.1.17 DGKADGGMDPNEY---

2.A.1.2.1 ------VTTMGFRMMLTEPIILSMGLYNFYAYGISYFFLTAIWPVFYDTYKM-SEMGASC

2.A.1.2.2 ---VSSLLWRPLEITMLEPVVFLIDIYIALVYSIMYLIFESVPIVYAGIHHF-TLVEMGA

2.A.1.2.6 ---IIDTLWRPLEITVMEPVVLLINIYIAMVYSILYLFFEVFPIYFVGVKHF-TLVELGT

2.A.1.2.17 ---LINTLYRPLKMIIKEPAILAFDLYIAVAYGCFYLFFEAFPIVFVGIYHF-SLVEVGL

2.A.1.2.35 ---AKETIIRPIILSISEPIVLSLNIYIGLIYSILYLWFEAFPILFTSVYHF-TIIENGL

2.A.1.2.16 ---VQKTVTRPIIMLFVEPLLLFVTIYNSFVYGILYLLLEAYPLVFVEGYGF-T-ENGEL

2.A.1.2.40 FPKPPKAGLTLYWKMIKCPPIIITSVSTALLFSSYYAFSVTFSYYLEHDYRF-TMLEIGA

2.A.1.2.31 LELPTQLNLLAPFKILKAYEICILMLVAGLQFAMYTTHLTALSTALSKQYHL-TVAKVGL

2.A.1.2.3 -------LLLP----VKCLNFWLYTLCYAAGMGSFFVFFSIAPGLMMGRQGV-SQLGFSL

2.A.1.2.4 ----------SFRWARGMTVVAALMAVFFIMQLVGQVPAALWVIFGEDRFHW-DATTIGI

2.A.1.2.38 ------------SYLITQPNMAPLLGIFLIITLVSQVPATLWVIYGQDRYGW-SIFIAGV

2.A.1.2.39 ------------RRLHGKPGLLPLAGIYLVMALVSQAPATLWILYGQDRFGW-SMMVAGL

2.A.1.2.41 ----------VFKEAFGRKELRGLMIGFGLLALPFGFFVNNFSVLALDSIQW-GPTQIGL

2.A.1.2.8 ----------KDLKRSIHPVYFIAFIIVFVMAFGLSAYETVFSLFSDHKFGF-TPKDIAA

2.A.1.2.10 ----------NW------KVFITPVILTLVLSFGLSAFETLYSLYTADKVNY-SPKDISI

2.A.1.2.24 ---------AI----KDSAYRSALFSAFANGWSNFGVRVAVLPLFAAAAFSN-GGAIAGF

2.A.1.2.20 ---------KN---PKLVLSLFVTTLIIQVATGSIAPILTLYVRELAGNVSN-VAFISGM

2.A.1.2.34 ---------KY---PYLLLNLFLTSFVIQFSAQSIGPILALYVR-DLGQTEN-LLFVSGL

2.A.1.2.7 -------------ALFRHKRVLSYMLASGFSFAGMFSFLSAGPFVYIEINHI-APENFGY

2.A.1.2.27 -------------EVLRNGNFTAPGLAFSLVFAAQGLLIGAAPDFIANVLDL-PETNFAY

2.A.1.2.19 -------------LVLKNGRFVAGALALGFVSLPLLAWIAQSPIIIITGEQL-SSYEYGL

2.A.1.2.14 -------------HFLRSPAPWLIFAATMFGNAGVFAWFSYVKPYMMFISGF-SETAMTF

2.A.1.2.15 -------------LLFRRPALMSIYLLTVVVVTAHYTAYSYIEPFVQNIAGF-SANFATA

2.A.1.2.18 -------------KLFRRPALVGIYLLTVVVVTAHFTAYSYIEPFIQTVAGL-PENFTTL

2.A.1.2.25 -------------KTIGNLKIASSHLVTMFTLAGHYTLYAYFAPFLEETLHL-SSFWVSI

2.A.1.2.26 -------------RLLQRPGVMAGMIAIFMSFAGQFAFFTYIRPVYMNLAGF-GVDGLTL

2.A.1.2.11 ---------------LKDPYILVAAGSICLANMGVAILEPTLPIWMMQTMCS-PEWQLGL

2.A.1.2.12 ---------------LKDPYILVAAGSICFANMGVAILEPTLPIWMMQTMCS-PKWQLGL

2.A.1.2.29 ---------------LKDPYILIAAGSICFANMGIAMLEPALPIWMMETMCS-RKWQLGV

2.A.1.2.28 ---------------MLDPYIAVVAGALTTCNIPLAFLEPTIATWMKHTMAA-SEWEMGM

2.A.1.2.30	-------PFASLKKVGKDSTVLLICITVFLSYLPEAGQYSSFFLYLRQVIGF-GSVKIAA
2.A.1.2.9	---------------FGNSGFNCYLLMLIGGLAGIAAFEACSGVLMGAVLGL-SSMTVSI
2.A.1.2.22	---------------LNRFFLSRVVITTLSVSVILTFVNTSPVLLMEIMGFE-RGEYATI
2.A.1.2.32	---------------LKTPRLLLAMLLGALVNAATFASFTFLAPVVTDTAGL-GDLWISV
2.A.1.2.21	---------------DKRFVTYVLTLAGYYMLAVQVMLMLPIMVNDVAGAPS-AVKWMYA
2.A.1.2.37	----------HSYTSKSVFFFLCVLIMLTQIAKFLPDPGFTLYLNKYCSNNL-VLIGFLY
2.A.1.2.5	------LQDKTYMIFMGANIATTFIIMQFDNFLPVHLSNSFKTITFWGFEIY-GQRMLTI
2.A.1.1.1	-----------ENSNFRRAVFLGVLLQVMQQFTGMNVIMYYAPKIF-ELAGYTNTTEQMW
2.A.1.1.2	-----------INRNVRRAVFLGMLLQAMQQFTGMNIIMYYAPRIF-KMAGFTTTEQQMI
2.A.1.1.26	-----K---DLSVPWVRRIVFIGLGIAIVQQITGVNSIMYYGTEIL-RNSGF-QTEAALI
2.A.1.1.53	-----GLREILSSKWLVRILLVGIGLGVAQQLTGINSIMYYGQVVL-IEAGF-SENAALI
2.A.1.1.55	---------QLFKPGLRKALVIGILLALFNQVIGMNAITYYGPEIF-KMMGF-GQNAGFV
2.A.1.1.25	-----VICRMLSYPPTRRALIVGCGLQMFQQLSGINTIMYYSATIL-QMSGVEDDRLAIW
2.A.1.1.63	-----NMIKLCKAKTVRRGLIAGVGLQVFQQFVGINTVMYYSPTIV-QLAGFASNRTALL
2.A.1.1.66	-----GYLDVFRSKELRLAFLAGAGLQAFQQFTGINTVMYYSPTIV-QMAGFHSNQLALF
2.A.1.1.32	---------LSRRGGLLPIVWIGMGLSALQQFVGINVIFYYSSVLW-RSVGF-TEEKSLL
2.A.1.1.35	---------LGGSFFFKPIVWIGIGLSVFQQFVGINVAFYYSSTLW-QSVGV-DPADSFF
2.A.1.1.65	---------KKPTGGIYGIVWVGLGLSIFQQFVGINVIFYYSNVLW-QAVGF-SADQSAI
2.A.1.1.6	------GELFSTKTKVFQRLLMGVFVQMFQQLTGNNYFFYYGTVIF-KSVGL---DDSFE
2.A.1.1.67	------KELITGKPKIFRRVIMGIMLQSLQQLTGDNYFFYYGTTIF-QAVGL---KDSFQ
2.A.1.1.36	-------HEVVTGPRMLYRTLLGIALQSLQQLSGANFIFYYGNSIF-TSTGL---NNSYV
2.A.1.1.58	-------HELFTGPRMLYRTLLGIALQSLQQLTGANFIFYYGNSIF-TSTGL---SNSYV
2.A.1.1.19	----DCFKTSENRPKQILRIFTGIAIQAFQQASGINFIFYYGVNFF-NNTGV---DNSYL
2.A.1.1.8	----NTIKELHTVPSNLRALIIGCGLQAIQQFTGWNSLMYFSGTIF-ETVGF---KNSSA
2.A.1.1.21	--------ASIFGKDIRYRTFLGMFVMSLQQLTGNNYFFYYGFSVM-QGAGI---NSPYL
2.A.1.1.23	--------IEIFGKDIRYRTCLGFLVMLFRELIGNNYYFYYATQVF-KGTGM---TDIFL

2.A.1.1.42 ----RIA---------RPALVAAIGIMLFQQLVGINSVIYFLPQVFIKGFGF-PEGDAIW

2.A.1.1.33 ----SMFDL-ILIPRNRRALLYSIMMVSLGQLTGINAIMYYMSTLM-GQIGF-SPKQAVA

2.A.1.1.18 ----DCFED-----KINRRRTRITCLCWAGQATCGSILIGYSTYFY-EKAGV-STEMSFT

2.A.1.1.20 ------------ARDMRFRVVLSSGLQIIQQFSGINTIMYYSSVIL-YDAGFRDAIMPVV

2.A.1.1.14 -------WASLFTRRYMPQLLTSFVIQFFQQFTGINAIIFYVPVLF-SSLGS-ANSAALL

2.A.1.1.50 -------FRNLLQRRNRPQLVIAVALQIFQQCTGINAIMFYAPVLF-STLGF-GSDASLY

2.A.1.1.60 -------WRNLLRRKYRPHLTMAVMIPFFQQLTGINVIMFYAPVLF-NTIGF-TTDASLM

2.A.1.1.61 -------WTNIMQARYRPQLTFCTFIPFFQQLTGINVIMFYAPVLF-KTIGF-GNDASLI

2.A.1.1.56 -------YRKLLKPASRPPFIIGMLLQLFQQFTGINAIMFYAPVLF-QTVGF-GSDAALL

2.A.1.1.37 -------LHLCALRSLRWQLLSIIVLMAGQQLSGINAINYYADTIY-TSAGV-EAAHSQY

2.A.1.1.47 -------LELLRAPFVRWQVITVIITMASYQLCGLNAIWFYTNSIF-GKAGI-PQDKIPY

2.A.1.1.52 -------TQLFTQRDLRMPVLIACLIQVLQQLSGINAVITYSSLML-ELAGI-PDVYLQY

2.A.1.1.51 -----WSQVFSHKNHQLKRLFTGVAIQAFQQLTGVNFIFYYGTTFF-KRAGV----NGFT

2.A.1.1.57 ----CFRGSIFSPNSNLRRTVLGTSLQMMQQWTGVNFVFYFGTTFF-QSLGT--IDDPFL

2.A.1.1.28 -------LELFRSPAYRQPILIAVVLQLSQQLSGINAVFYYSTSIF-EKAGV---QQPVY

2.A.1.1.29 -------IQLFTNSSYRQPILVALMLHVAQQFSGINGIFYYSTSIF-QTAGI---SKPVY

2.A.1.1.41 -----GGWSELFGKMVRPSLIIGIGLAIFQQVMGCNTVLYYAPTIF-TDVGF-GVSAALL

2.A.1.1.54 KHTHMSIGEVLSNKWLVRLLIAGIGVAVAQQLTGINAIMYYGTRVL-EESGM-SAEMAVV

2.A.1.1.24 ----LSLLSALKIPSYRYVIILGCLLSGLQQFTGINVLVSNSNELY-KEFLD--SHLITI

2.A.1.1.43 ARKSLSFAQLFRDPMCRKMTIICVTLSAIQQLSGINGVIFYSTSIF-SEVFA--DN-AKY

2.A.1.1.3 ------------LMFGVGVIVIGVMLSIFQQFVGINVVLYYAPEVF-KTLGA-STDIALL

2.A.1.1.4 ------------FAFGITVVFAGVSVAAFQQLVGINAVLYYAPQMF-QNLGF-GADTALL

2.A.1.1.46 ------------RPGIYKPLIIGISLMVFQQLSGVNAIMFYANSIF-EEAKF---KDSSL

2.A.1.1.59 ------------RDNMRGRTTVGLGLVLFQQLTGQPNVLCYASTIF-SSVGFHGGSSAVL

2.A.1.1.17 -----------GWGQMLWPLFMGAVTAGTLQLTGINAVMNYAPKIT-ENLGM----DPSL

2.A.1.2.1 TYLSGFVASTLLFLYQPIQDWIFRRDK--AKNNGVARPE---------------------

2.A.1.2.2 TYVSTIIGIIIGGAIYLPTVYYKFTKKL-LAGQ-NVTPE---------------------

2.A.1.2.6 TYMSIVIGIVIAAFIYIPVIRQKFTKPI-LRQE-QVFPE---------------------

2.A.1.2.17 AYMGFCVGCVLAYGLFGI-LNMRIIVPR-FRNG-TFTPE---------------------

2.A.1.2.35 VYLGILVGALITLACYFVFLYKVMIPAF-MASGGDFAPE---------------------

2.A.1.2.16 PYIALIIGMMVCAAFIWYMDNDYLKRCR-AKGKLVPEAR---------------------

2.A.1.2.40 AYVCPGVAMLLGSQSGGHLSDYLRSRWIKSHPKKKFPAE---------------------

2.A.1.2.31 CYLPSGICTLCSIVIAGRYLNWNYRRRLKYYQNWLGKKRSKLLEEHDNDLNLVQRIIEND

2.A.1.2.3 LFATVAIAMVFTARFMGRVIPKWG------------------------------------

2.A.1.2.4 SLAAFGILHSLAQAMITGPVAARL------------------------------------

2.A.1.2.38 SLASYGICHSIAQAFAIAPMVKRF------------------------------------

2.A.1.2.39 SLAGYGACHALSQAFAIGPLVARL------------------------------------

2.A.1.2.41 LTAAVGIIDILIQGVLLGILLPRI------------------------------------

2.A.1.2.8 IITISSIVAVVIQVLLFGKLVNKL------------------------------------

2.A.1.2.10 AITGGGIFGALFQIYFFDKFMKYF------------------------------------

2.A.1.2.24 AMAAFAAGNALCLQFAGDLSDRIG------------------------------------

2.A.1.2.20 IASVPGVAALLSAPRLGKLGDRIG------------------------------------

2.A.1.2.34 IVSSMGFSSMMSAGVMGKLGDKVG------------------------------------

2.A.1.2.7 YFALNIVFLFVMTIFNSRFVRRIG------------------------------------

2.A.1.2.27 LFVPLVAGAMTGAAIAARHAGRWR------------------------------------

2.A.1.2.19 LQVPIFGALIAGNLLLARLTSRRT------------------------------------

2.A.1.2.14 IMMLVGLGMVLGNMLSGRISGRYS------------------------------------

2.A.1.2.15 LLLLLGGAGIIGSVIFGKLGNQYA------------------------------------

2.A.1.2.18 ILLLFGCAGIAGSMLYSRYSDRFP------------------------------------

2.A.1.2.25 CYFLFGISAVCGGPFGGALSDRLG------------------------------------

2.A.1.2.26 VLLSFGIASFIGTSLSSFILKRSV--------------------------------------

2.A.1.2.11 AFLPASVAYLIGTNLFGVLANKMG--------------------------------------

2.A.1.2.12 AFLPASVSYLIGTNLFGVLANKMG--------------------------------------

2.A.1.2.29 AFLPASISYLIGTNIFGILAHKMG--------------------------------------

2.A.1.2.28 AWLPAFVPHVLGVYLTVRLAARYP--------------------------------------

2.A.1.2.30 FIAMVGILSIVAQTAFLSILMRSL--------------------------------------

2.A.1.2.9 LFILPIPAAFFGAWFAGRPNKRFS--------------------------------------

2.A.1.2.22 MALTAGVSMTVSFSTPFALGIFKP--------------------------------------

2.A.1.2.32 ALVLFGAGSFAGVTVAGRLSDRRP--------------------------------------

2.A.1.2.21 IEACLSLTLLYPIARWSEKHFRLE--------------------------------------

2.A.1.2.37 SLPAMGMLCSSVWCGKQFDYCRSQ--------------------------------------

2.A.1.2.5 YLILACVLVVLLMTTLNRLTKDWS--------------------------------------

2.A.1.1.1 GTVIVGLTNVLATFIAIGLVDRWG--------------------------------------

2.A.1.1.2 ATLVVGLTFMFATFIAVFTVDKAG--------------------------------------

2.A.1.1.26 GNIANGVISVLATFVGIWLLGRVG--------------------------------------

2.A.1.1.53 ANVAPGVIAVVGAFIALWMMDRIN--------------------------------------

2.A.1.1.55 TTCIVGVVEVIFTVIAVLLIDKVG--------------------------------------

2.A.1.1.25 LASVTAFTNFIFTLVGVWLVEKVG--------------------------------------

2.A.1.1.63 LSLVTAGLNAFGSIISIYFIDRIG--------------------------------------

2.A.1.1.66 LSLIVAAMNAAGTVVGIYFIDHCG--------------------------------------

2.A.1.1.32 ITVITGFINILTTLVAIAFVDKFG--------------------------------------

2.A.1.1.35 YSFTTSIINIVGTVIAMIFVDRVG--------------------------------------

2.A.1.1.65 YTVITSVVNVLTTLIAIALIDKIG--------------------------------------

2.A.1.1.6 TSIVIGVVNFASTFFSLWTVENLG--------------------------------------

2.A.1.1.67 TSIILGIVNFASTFVGIYVIERLG--------------------------------------

2.A.1.1.36 TQIILGAVNFGMTLPGLYIVEHFG--------------------------------------

2.A.1.1.58 TQIILGAVNFGMTLPGLYVVEHFG--

2.A.1.1.19 VSFISYAVNVAFSIPGMYLVDRIG--

2.A.1.1.8 VSIIVSGTNFIFTLVAFFSIDKIG--

2.A.1.1.21 SAMILDAVNFGCTFGGMYVLERFG--

2.A.1.1.23 PAVILGAINFGTTFGALYTIDNLG--

2.A.1.1.42 VSVGIGVVNFVSTIVATLIMDRFP--

2.A.1.1.33 MSMVGGAALLIGTIPAILYMDKFG--

2.A.1.1.18 FSIIQYCLGICATFLSWWASKYFG--

2.A.1.1.20 LSIPLAFMNALFTAVAIFTVDRFG--

2.A.1.1.14 NTVVVGAVNVGSTLIAVMFSDKFG--

2.A.1.1.50 SAVVTGAVNVLSTLVSIYSVDKVG--

2.A.1.1.60 SAVVTGSVNVAATLVSIYGVDRWG--

2.A.1.1.61 SAVITGLVNVLSTIVSIYSVDKFG--

2.A.1.1.56 SAVITGSINVLATFVGIYLVDRTG--

2.A.1.1.37 VTVGSGVVNIVMTITSAVLVERLG--

2.A.1.1.47 ITLSTGGIETLAAIFSGLVIERLG--

2.A.1.1.52 CVFAIGVLNVIVTVVSLPLIERAG--

2.A.1.1.51 ISLATNIVNVGSTIPGILLMEVLG--

2.A.1.1.57 ISMITTIVNVCSTPVSFYTIEKFG--

2.A.1.1.28 ATIGSGIVNTAFTVVSLFVVERAG--

2.A.1.1.29 ATIGVGAVNMVFTAVSVFLVEKAG--

2.A.1.1.41 AHIGIGIFNVIVTAIAVAIMDKID--

2.A.1.1.54 ANIAFGAVAVIGGLIALRNMDRLD--

2.A.1.1.24 LSVVMTAVNFLMTFPAIYIVEKLG--

2.A.1.1.43 ATVGVGVINLIFTMVSVILIDRQG--

2.A.1.1.3 QTIIVGVINLTFTVLAIMTVDKFG--

2.A.1.1.4 QTISIGVVNFIFTMIASRVVDRFG------------------------------------

2.A.1.1.46 ASVTVGIIQVLFTAVAALIMDRAG------------------------------------

2.A.1.1.59 ASVGLGAVKVAATLTAMGLVDRAG------------------------------------

2.A.1.1.17 GNFLVMAWNFVTSLVAIPLASRFT------------------------------------

2.A.1.2.1 ---------ARFTSALFITLLFPAGMFLFAFTC---------------------------

2.A.1.2.2 ---------VFLPPAIFGAICMPIGVFIFGWTS---------------------------

2.A.1.2.6 ---------VFIPIAIVGGILLTSGLFIFGWSA---------------------------

2.A.1.2.17 ---------AFLIVAMCVCWCLPLSLFLFGWTA---------------------------

2.A.1.2.35 ---------GVLVISFPATFFIPICLFWFGWSG---------------------------

2.A.1.2.16 ---------LYAMVIAGTVFPIGILWFCWTGYY---------------------------

2.A.1.2.40 ---------FRLLLNLIGILLTICGTIGYGWAI---------------------------

2.A.1.2.31 PKYTFNIFKARLQPAFVTLLLSSSGFCAYGWCI---------------------------

2.A.1.2.3 ----------SPSVLRMGMGCLIAGAVLLAITE---------------------------

2.A.1.2.4 ---------GERRALMLGMIADGTGYILLAFAT---------------------------

2.A.1.2.38 ---------GEKNTLLCGIACDAIGLLLLSIAV---------------------------

2.A.1.2.39 ---------GERKALLIGLAADAVGLALLSVAT---------------------------

2.A.1.2.41 ---------GERGVIVSGIVAQMIGLAALAVVA---------------------------

2.A.1.2.8 ---------GEKRMIQLCLITGAILAFVSTVMS---------------------------

2.A.1.2.10 ---------SELTFIAWSLLYSVVVLILLVFAN---------------------------

2.A.1.2.24 ----------RKPMIISGLIVNAVFTAMIGFGT---------------------------

2.A.1.2.20 ----------PEKILITALIFSVLLLIPMSYVQ---------------------------

2.A.1.2.34 ----------NHRLLVVAQFYSVIIYLLCANAS---------------------------

2.A.1.2.7 ---------ALNMFRSGLWIQFIMAAWMVISAL---------------------------

2.A.1.2.27 ---------DTRIIGLAYLLMNGSCLAYAIYFW---------------------------

2.A.1.2.19 ---------VRSLIIMGGWPIMIGLLVAAAATV---------------------------

2.A.1.2.14 ----------PLRIAAVTDFIIVLALLMLFFCG---------------------------

2.A.1.2.15 ----------SALVSTAIALLLVCLALLLPAAN---------------------------

2.A.1.2.18 ----------IGFLVTAMLLLLACLTLLMPLSG---------------------------

2.A.1.2.25 ----------SFKSILLVTGSFAIIMFLLPLST---------------------------

2.A.1.2.26 ----------KLALAGAPLILAVSALVLTLWGS---------------------------

2.A.1.2.11 -----------RWLCSLVGMVAVGISLLCVPLA---------------------------

2.A.1.2.12 -----------RWLCSLIGMLVVGTSLLCVPLA---------------------------

2.A.1.2.29 -----------RWLCALLGMIIVGVSILCIPFA---------------------------

2.A.1.2.28 ---------HLQWLYGALGLAVIGASSCIVPAC---------------------------

2.A.1.2.30 ---------GNKNTVLLGLGFQMLQLAWYGFGS---------------------------

2.A.1.2.9 -----------TLMWQSVICCLLAGLLMWIPDW---------------------------

2.A.1.2.22 -----------RTLMITSQVLFLAAGITLAVSP---------------------------

2.A.1.2.32 -----------AQVLAVAGPLLLVGWPALAMLA---------------------------

2.A.1.2.21 -----------HRLMAGLLIMSLSMMPVGMVSG---------------------------

2.A.1.2.37 ---------PSLVNQYLIRYSVFGAILMIIQAN---------------------------

2.A.1.2.5 ---------HQK-GFIWGSLFMAIGMIFSFLTT---------------------------

2.A.1.1.1 ----------RKPTLTLGFLVMAAGMGVLGTMMHI---GIH-------------------

2.A.1.1.2 ----------RKPALKIGFSVMALGTLVLGYCLMQFDNGTA-------------------

2.A.1.1.26 ----------RRPMLMTGLIGTTTALLLIGIFSLVLEG----------------------

2.A.1.1.53 ----------RRTTLITGYSLTTISHVLIGIASVAFPV----------------------

2.A.1.1.55 ----------RKKLMSIGSAFMAIFMILIGTSFYFELT----------------------

2.A.1.1.25 ----------RRKLTFGSLAGTTVALIILALGFVLSAQVSPRITFKPIAPSGQNATCTRY

2.A.1.1.63 ----------RKKLLIISLFGVIISLGILTGVFYEAATHAPAISSLETQRFNNISCPDYK

2.A.1.1.66 ----------RKKLALSSLFGVIISLLILSVSFFKQSETSSD------------------

2.A.1.1.32 ----------RKPLLLMGSIGMTITLGILSVVFGGATV-VNGQ-----------------

2.A.1.1.35	----------RKPLALIGSVGMVIGLALEAWAFSFDLV----D-----------------
2.A.1.1.65	----------RKPLLLIGSSGMAVTLATMAVIFANATVKPDGT-----------------
2.A.1.1.6	----------HRKCLLLGAATMMACMVIYASVGVTRLYPHG-------------------
2.A.1.1.67	----------RRLCLLTGSAAMFICFIIYSLIGTQHLYKQGYSN----------------
2.A.1.1.36	----------RRASLMVGGAWMAICFYIWASVGNSVLDLDNPQN----------------
2.A.1.1.58	----------RRNSLMVGAAWMFICFMIWASVGHFALDLADPQA----------------
2.A.1.1.19	----------RRPVLLAGGVIMAIANLVIAIVGVSEG-----------------------
2.A.1.1.8	----------RRTILLIGLPGMTMALVVCSIAFHFLGIKFDGAVA---------------
2.A.1.1.21	----------RRNPLIIGGIWQSICFFIYSAVGSRALYHKNG------------------
2.A.1.1.23	----------RRNPLIFGAAFQSICFFIYAAVGDRKLIYKNG------------------
2.A.1.1.42	----------RKGMLIFGSIVMTVSLAVLAVMNFVG------------------------
2.A.1.1.33	----------RRPWSMTIIGFSVGLVLVGVGYQIDLN-----------------------
2.A.1.1.18	----------RYDLYAFGLAFQTIVFFIIGGLGCS-------------------------
2.A.1.1.20	----------RRRMLLISVFGCLVLLVVIAIIGFFIGTRI--------------------
2.A.1.1.14	----------RRFLLIEGGIQCCLAMLTTGVVLAIEFAK-YGT-----------------
2.A.1.1.50	----------RRVLLLEAGVQMFFSQVVIAIILGVKVT--DTS-----------------
2.A.1.1.60	----------RRFLFLEGGTQMLICQAVVAACIGAKFGVDGTP-----------------
2.A.1.1.61	----------RRALFLQGGFQMIVTQIAVGSMIGWKFGF-NGE-----------------
2.A.1.1.56	----------RRFLLLQSSVHMLICQLIIGIILAKDLG---VT-----------------
2.A.1.1.37	----------RRHLLLAGYGICGSACLVLTVVLLFQNR----------------------
2.A.1.1.47	----------RGPLLIGGFGLMALFFGTLTATLTLQDQ----------------------
2.A.1.1.52	----------RRTLLLWPTVSLALSLLLLTIFVNLADS---GP-----------------
2.A.1.1.51	----------RRNMLMGGATGMSLSQLIVAIVGVATSE----------------------
2.A.1.1.57	----------RRSLLLWGALGMVICQFIVAIVGTVDGS----------------------
2.A.1.1.28	----------RRTLHLIGLAGMAGCAILMTIALALLEQ----------------------
2.A.1.1.29	----------RRSLFLIGMSGMFVCAIFMSVGLVLLNK----------------------

2.A.1.1.41 ----------RKKIVNIGAVGMGISLFVMSIGMKFSGG----------------------

2.A.1.1.54 ----------RRTTFIIGLSLTTTFHLLIAAAGTLLPEG---------------------

2.A.1.1.24 ----------RKTLLLWGCVGVLVAYLPTAIANEINRN----------------------

2.A.1.1.43 ----------RKRLLLASEIGIVVTSILVVLGSIYS------------------------

2.A.1.1.3 ----------RKPLQIIGALGMAIGMFSLGTAFYTQ------------------------

2.A.1.1.4 ----------RKPLLIWGALGMAAMMAVLGCCFWFK------------------------

2.A.1.1.46 ----------RRLLLALSGVIMVFSMSAFGTYFKLTQSLPSNSSHVGLVPIA--------

2.A.1.1.59 ----------RRALLLAGCALMALSVSGIGLVSFAVPMDSGPSCLAVPNATGQTGLPGDS

2.A.1.1.17 ----------MRQMFITCSFVASCMCLFLCGIPVFPGVA---------------------

2.A.1.2.1 --

2.A.1.2.2 --

2.A.1.2.6 --

2.A.1.2.17 --

2.A.1.2.35 --

2.A.1.2.16 --

2.A.1.2.40 --

2.A.1.2.31 --

2.A.1.2.3 --

2.A.1.2.4 --

2.A.1.2.38 --

2.A.1.2.39 --

2.A.1.2.41 --

2.A.1.2.8 --

2.A.1.2.10 --

```
2.A.1.2.24      ------------------------------------------------------------
2.A.1.2.20      ------------------------------------------------------------
2.A.1.2.34      ------------------------------------------------------------
2.A.1.2.7       ------------------------------------------------------------
2.A.1.2.27      ------------------------------------------------------------
2.A.1.2.19      ------------------------------------------------------------
2.A.1.2.14      ------------------------------------------------------------
2.A.1.2.15      ------------------------------------------------------------
2.A.1.2.18      ------------------------------------------------------------
2.A.1.2.25      ------------------------------------------------------------
2.A.1.2.26      ------------------------------------------------------------
2.A.1.2.11      ------------------------------------------------------------
2.A.1.2.12      ------------------------------------------------------------
2.A.1.2.29      ------------------------------------------------------------
2.A.1.2.28      ------------------------------------------------------------
2.A.1.2.30      ------------------------------------------------------------
2.A.1.2.9       ------------------------------------------------------------
2.A.1.2.22      ------------------------------------------------------------
2.A.1.2.32      ------------------------------------------------------------
2.A.1.2.21      ------------------------------------------------------------
2.A.1.2.37      ------------------------------------------------------------
2.A.1.2.5       ------------------------------------------------------------
2.A.1.1.1       ------------------------------------------------------------
2.A.1.1.2       ------------------------------------------------------------
2.A.1.1.26      ----------------------------------------------------------S-
2.A.1.1.53      ----------------------------------------------------------G-
```

```
2.A.1.1.55    ----------------------------------------------------------S-
2.A.1.1.25    SYCNECMLDPDCGFCXKMNKSTVIDSSCVPVNKASTNEAAWGRCENETKFKTEDIFWAYN
2.A.1.1.63    SAMNTNAWDCMTCLKASSPSCGYCSSPIGKEHPGACWISDDS---VKDLCHNENRLWYTR
2.A.1.1.66    ------------------------------------------------------------
2.A.1.1.32    --------------------------------------------------------PTL-
2.A.1.1.35    --------------------------------------------------------GKL-
2.A.1.1.65    --------------------------------------------------------PDL-
2.A.1.1.6     -------------------------------------------------------KSQPS-
2.A.1.1.67    -------------------------------------------------------ETSNT-
2.A.1.1.36    ------------------------------------------------------------
2.A.1.1.58    ------------------------------------------------------------
2.A.1.1.19    ----------------------------------------------------------K-
2.A.1.1.8     ------------------------------------------------------VVVSSG-
2.A.1.1.21    ---------------------------------------------------------TS-
2.A.1.1.23    ---------------------------------------------------------TS-
2.A.1.1.42    ----------------------------------------------------------D-
2.A.1.1.33    ----------------------------------------------------------T-
2.A.1.1.18    ----------------------------------------------------------S-
2.A.1.1.20    ----------------------------------------------------------S-
2.A.1.1.14    --------------------------------------------------------DPL-
2.A.1.1.50    --------------------------------------------------------TNL-
2.A.1.1.60    --------------------------------------------------------GEL-
2.A.1.1.61    --------------------------------------------------------GNL-
2.A.1.1.56    --------------------------------------------------------GTL-
2.A.1.1.37    ------------------------------------------------------------
2.A.1.1.47    ------------------------------------------------------------
```

2.A.1.1.52 --QST-

2.A.1.1.51 --N-

2.A.1.1.57 --N-

2.A.1.1.28 --

2.A.1.1.29 --

2.A.1.1.41 --

2.A.1.1.54 --

2.A.1.1.24 --

2.A.1.1.43 --

2.A.1.1.3 --

2.A.1.1.4 --

2.A.1.1.46 ---AEPVDV-

2.A.1.1.59 GLLQDSSLPPIPRTNEDQREPILSTAKKTKPHPRSGDPSAPPRLALSSALPGPPLPARG-

2.A.1.1.17 --E-

2.A.1.2.1 --HPPFPWMSPIVGNSMVTVANGHNWMCILNYLTDSY-PLLSGSAVAAFTLPSFIGATVF

2.A.1.2.2 --SPDINWFVPLIGMALFAVGAFIIFQTLFNYMAVSFKVEYLASVFSSNAFFRSVSAGAF

2.A.1.2.6 --NRTTHWVGPLFGAATTASGAFLIFQTLFNFMGASFKPHYIASVFASNDLFRSVIASVF

2.A.1.2.17 --R--VHWILPVISEVFFVLAVFNIFQATFAYLATCY-PKYVASVFAGNGFCRASFACAF

2.A.1.2.35 --RESVHWIVPIVSTLFYASGAFLMFQSMFQYLAASY-PKYVASVFAGNDLFRSAMAASF

2.A.1.2.16 --PHKIHWMVPTVGGAFIGFGLMGIFLPCLNYIIESY-LLLAASAVAANTFMRSAFGACF

2.A.1.2.40 --FFHYHFVVLLVFSALTAFGMTWCSNTSMTYLTELF-PKRAAGTVAVSSFFRNVGAAIS

2.A.1.2.31 --TVKAPLAAVLCMSGFASLFSNCILTFSTTLIVDLF-PTKTSTATGCLNLFRCILSAVF

2.A.1.2.3 --IWALQSVLGFIAPMWLVGIGVATAVSVAPNGALRGFDHVAGTVTAVYFCLGGVLLGSI

2.A.1.2.4 --RGW-----MAFPIMVLLASGGIGMPALQAMLSRQVDEERQGQLQGSLAALTSLTSIVG

2.A.1.2.38 --EEW-----VPFALLPLFALGGVAVPALQAMMSRGISDERQGELQGLLSSFNSLGAIIG

2.A.1.2.39 --RGW-----APFALLPFFAAGGMALPALQALMAHKVDDDHQGELQGTLASMGSLIGVAG

2.A.1.2.41 --SVFAQPWVFIVGALMLAAGQGASQAAMDGAMSNAVGDDEQGWLGGATQSLNAAMGTAA

2.A.1.2.8 --GFL-----TVLLVTCFIFLAFDLLRPALTAHLSNMAGNQQGFVAGMNSTYTSLGNIFG

2.A.1.2.10 --GYW-----SIMLISFVVFIGFDMIRPAITNYFSNIAGERQGFAGGLNSTFTSMGNFIG

2.A.1.2.24 --EVWI----LITVSALAGAGAGLLNPSQQAVLADVIDSRPGGKVLANFQMAQDFGAIVG

2.A.1.2.20 --TPLQ----LGILRFLLGAADGALLPAVQTLLVYNSSNQIAGRIFSYNQSFRDIGNVTG

2.A.1.2.34 --SPLQ----LGLYRFLFGLGTGALIPGVNALLSKMTPKAGISRVFAFNQVFFYLGGVVG

2.A.1.2.7 --LGL--GFWSLVVGVAAFVGCVSMVSSNAMAVILDEFPHMAGTASSLAGTFRFGIGAIV

2.A.1.2.27 --TALSPALPWAVLPPALFTCGLAMSVPAMTLHILESVPALSGTGASVLGFMQMLAFSIA

2.A.1.2.19 --ISSH-AYLWMTAGLSIYAFGIGLANAGLVRLTLFASDMSKGTVSAAMGMLQMLIFTVG

2.A.1.2.14 --GMKT---T-SLIFAFICCAGLFALSAPLQILLLQNAKGGELLGAAGGQIAFNLGSAVG

2.A.1.2.15 --SE-I---H-LGVLSIFWGIAMMIIGLGMQVKVLALAPDATDVAMALFSGIFNIGIGAG

2.A.1.2.18 --YP-F---G-LTLLCLVWGLAMMSIGLAMQAKVLSLAPDASDVAMSIFSGLFNLGIGGG

2.A.1.2.25 --SSMI---F-FLPVMVIWGLLSWSLAPAQQSYLIEIAPDSSDIQQSFNTSALQVGIALG

2.A.1.2.26 --DK-I---V-ATGVAIIWGLTFALVPVGWSTWITRSLADQAEKAGSIQVAVIQLANTCG

2.A.1.2.11 --HNIFGLIGPNAGLGFAIGMVDSSLMPIMGYLVDLRHTSVYGSVYAIADVAFCVGFAIG

2.A.1.2.12 --HNIFGLIGPNAGLGLAIGMVDSSMMPIMGHLVDLRHTSVYGSVYAIADVAFCMGFAIG

2.A.1.2.29 --KNIYGLIAPNFGVGFAIGMVDSSMMPIMGYLVDLRHVSVYGSVYAIADVAFCMGYAIG

2.A.1.2.28 --RSFAPLVVSLCGLCFGIALVDTALLPTLAFLVDVRHVSVYGSVYAIADISYSVAYALG

2.A.1.2.30 --QAWM-----MWAAGTVAAMSSITFPAISALVSRNAESDQQGVAQGIITGIRGLCNGLG

2.A.1.2.9 --FGV-----MNVWTLLVPAALFFFGAGMLFPLATSGAMEPFPFLAGTAGALVGGLQNIG

2.A.1.2.22 --SHA-----VSLFGITLICAGFSVGFGVAMSQALGPFSLRAGVASSTLGIAQVCGSSLW

2.A.1.2.32 --DRP-----VALLTLVFVQGALSFALGSTLITRVLYEAAGAPTMAGSYATAALNVGAAA

2.A.1.2.21 --LQQ-----LFTLICLFYIGSIIAEPARETLSASLADARARGSYMGFSRLGLAIGGAIG

2.A.1.2.37 --VD-------NFYLFALIRILWGIVLAALLPALFTLCSDRNLLPGYALGLANSFAKLGN

2.A.1.2.5	--TFTP----IFIAGIVYTLGEIVYTPSVQTLGADLMNPEKIGSYNGVAAIKMPIASILA
2.A.1.1.1	--SPSAQYFAIAMLLMFIVGFAMSAGPLIWVLCSEIQPLKGRDFGITCSTATNWIANMIV
2.A.1.1.2	--SSGLSWLSVGMTMMCIAGYAMSAAPVVWILCSEIQPLKCRDFGITCSTTTNWVSNMII
2.A.1.1.26	---PALPYVVLSLTVTFLAFQQGAISPVTWLMLSEIFPLRLRGLGMGVTVFCLWMVNFAV
2.A.1.1.53	--DPLRPYVILTLVVVFVGSMQTFLNVATWVMLSELFPLAMRGFAIGISVFFLWIANAFL
2.A.1.1.55	------GIMMIVLILGFVAAFCVSVGPITWIMISEIFPNHLRARAAGIATIFLWGANWAI
2.A.1.1.25	FCPTPYSWTALLGLILYLVFFAPGMGPMPWTVNSEIYPLWARSTGNACSSGINWIFNVLV
2.A.1.1.63	GCPSNFGWFALLGLGLYIIFFSPGMGTVPWIVNSEIYPLRFRGICGGIAATANWISNLIV
2.A.1.1.66	--GGLYGWLAVLGLALYIVFFAPGMGPVPWTVNSEIYPQQYRGICGGMSATVNWISNLIV
2.A.1.1.32	--TGAAGIIALVTANLYVFSFGFSWGPIVWVLLGEMFNNKIRAAALSVAAGVQWIANFII
2.A.1.1.35	--PATQGWVALIAAHVFVLFFALSWGVVVWVFLGEMFPNRIRAAALGVAASAQWIANWAI
2.A.1.1.65	--PGASGLIALIAANLFVVAFGMSWGPVVWVLLGEMFPNRFRAAALGLAAAGQWAANWLI
2.A.1.1.6	--SKGAGNCMIVFTCFYIFCYATTWAPVAWVITAESFPLRVKSKCMALASASNWVWGFLI
2.A.1.1.67	--YKASGNAMIFITCLYIFFFASTWAGGVYCIISESYPLRIRSKAMSIATAANWLWGFLI
2.A.1.1.36	--TPKAGAAMIVFTCFFIAGFATTWGPIVWSICSEMYPNRSRATSIGIATCANWTWNFLI
2.A.1.1.58	--TPAAGKAMIIFTCFFIVGFATTWGPIVWAICGEMYPARYRALCIGIATAANWTWNFLI
2.A.1.1.19	--TVVASKIMIAFICLFIAAFSATWGGVVWVVSAELYPLGVRSKCTAICAAANWLVNFTC
2.A.1.1.8	--FSSWGIVIIVFIIVFAAFYALGIGTVPWQQ-SELFPQNVRGIGTSYATATNWAGSLVI
2.A.1.1.21	--NTRAGAVMIVMACLFIFGFAQTWAPAAYVIVGESYPVRYRSKCAAVATASNWLWNFLI
2.A.1.1.23	--DHRAGSVMIVFSCLFLFSYCCSWGPMGWVIVGETFPIRYRSKCASVATSGNWLGNFMI
2.A.1.1.42	--VAVLAVPTMILIAFYILGFAVSWGPIAWVLIGEIFPLSVRGIGSSFGSAANWLGNFIV
2.A.1.1.33	--NLAAAEGVYLTGQILYNIFFGTYATLTWVVPSESFSLATRSIGMTICSAFLYLWAFTV
2.A.1.1.18	--THGSKMGSGSLLMAVAFFYNLGIAPVVFCLVSEMPSSRLRTKTIILARNTYNVVSIIC
2.A.1.1.20	--YSVGGGLFLALLAVFLALYAPGIGCIPWVIMGEIFPTHLRTSAASVATMANWGANVLV
2.A.1.1.14	--PKAVASGILAVICIFISGFAWSWGPMGWLIPSEIFTLETRPAGTAVAVVGNFLFSFVI
2.A.1.1.50	--SKGFAILVVVMICTYVAAFAWSWGPLGWLIPSETFPLETRSAGQSVTVCVNLLFTFII

2.A.1.1.60	--PKWYAIVVVTFICIYVAGFAWSWGPLGWLVPSEIFPLEIRSAAQSITVSVNMIFTFII
2.A.1.1.61	--SGVDADIILALICLYVAGFAWSWGPLGWLVPSEICPLEIRSAGQSLNVSVNMFFTFFI
2.A.1.1.56	--GRPQALVVVIFVCVYVMGFAWSWGPLGWLIPSETFPLETRSAGFAVAVSCNMFFTFVI
2.A.1.1.37	--VPELSYLGIICVFAYIAGHSIGPSPVPSVVRTEIFLQSSRRAAFMVDGAVHWLTNFII
2.A.1.1.47	--APWVPYLSIVCILAIIASFCSGPGGIPFILTGEFFQQSERPAAFMIAGTVNWLSNFAV
2.A.1.1.52	--KNAMGIISIILILIYICSFALGLGPVPALIVSEIFRQGPRAAAYSLSQSIQWLSNLIV
2.A.1.1.51	--NKSSQSVLVAFSCIFIAFFAATWGPCAWVVVGELFPLRTRAKSVSLCTASNWLWNWGI
2.A.1.1.57	---KHAVSAEISFICIYIFFFASTWGPGAWVVIGEIFPLPIRSRGVALSTASNWLWNCII
2.A.1.1.28	--LPWMSYLSIVAIFGFVAFFEVGPGPIPWFIVAELFSQGPRPAAIAVAGFSNWTSNFIV
2.A.1.1.29	--FSWMSYVSMIAIFLFVSFFEIGPGPIPWFMVAEFFSQGPRPAALAIAAFSNWTCNFIV
2.A.1.1.41	--SQTAAIISVIALTVYIAFFSATWGPVMWVMIGEVFPLNIRGLGNSFASVINWTANMIV
2.A.1.1.54	--NSIRPFAIMILVVGFVLSMQTFLNVAVWVWLAEIFPVRMKGIGTGISVFCGWGINGVL
2.A.1.1.24	--SNFVKILSIVATFVMIISFAVSYGPVLWIYLHEMFPSEIKDSAASLASLVNWVCAIIV
2.A.1.1.43	-----INLLVVVAVLLFVSSFAIGLGPIPFLIIPELLPTYGVSAAASLAMGLNWLSNFLV
2.A.1.1.3	----APGIVALLSMLFYVAAFAMSWGPVCWVLLSEIFPNAIRGKALAIAVAAQWLANYFV
2.A.1.1.4	----VGGVLPLASVLLYIAVFGMSWGPVCWVVLSEMFPSSIKGAAMPIAVTGQWLANILV
2.A.1.1.46	--QVGLAWLAVGSMCLFIAGFAVGWGPIPWLLMSEIFPLHVKGVATGICVLTNWFMAFLV
2.A.1.1.59	--HALLRWTALLCLMVFVSAFSFGFGPVTWLVLSEIYPVEIRGRAFAFCNSFNWAANLFI
2.A.1.1.17	--EKVKNGVATTGIALFIAAFEFGVGSCFFVLAQDLFPPSFRPKGSSFVVMMQFIFNILI

2.A.1.2.1	AHVSQIMFNN-----------MS--------------------VKWAVATMAFISISIPF
2.A.1.2.2	PLFGRALYNN-----------LSIDK---------------FPVGWGSSILGFISLGMIA
2.A.1.2.6	PLFGAPLFDN-----------LATPE---------------YPVAWGSSVLGFITLVMIA
2.A.1.2.17	PLFGRAMYDN-----------LATKN---------------YPVAWGSSLVGFLTLGLAI
2.A.1.2.35	PLFARAMFNN-----------TGPSY---------------APVAWGSTILGIVACLMIP

2.A.1.2.16 PLFAGYMFRG-----------MG--------------------IGWAGLLLGLFAAAMIP

2.A.1.2.40 SAIILQLCNA-----------MG--------------------IGWCFTGLGLCSSISLI

2.A.1.2.31 IAALSKMVEK-----------MK--------------------FGGVFTFLGALTSSSSI

2.A.1.2.3 GTLIISLLPR-----------NTAW-----------------PVVVYCLTLATVVLGLSC

2.A.1.2.4 PLLFTAIYAA-----------SITT-----------------WNGWAWIAGAALYLLCLP

2.A.1.2.38 PVLVTSLYFM-----------TQAS-----------------APGMVWALAAILYVITLP

2.A.1.2.39 PLVATALYAA-----------TRDV-----------------WPGLVWALAAALYLVVPP

2.A.1.2.41 PLIAGALYAL-----------VSHA-----------------APYWLGVALMIVAVTVVS

2.A.1.2.8 PALGGILFDL-----------NIH------------------YPFLFAGFVMIVGLGLTM

2.A.1.2.10 PLIAGALFDV-----------HIE------------------APIYMAIGVSLAGVVIVL

2.A.1.2.24 PILVGMIAEQ-----------AGF------------------QIGFMLCGVISLLAAVAW

2.A.1.2.20 PLMGAAISAN-----------YGF------------------RAVFLVTAGVVLFNAVYS

2.A.1.2.34 PMAGSAVAGQ-----------FGY------------------HAVFYATSLCVAFSCLFN

2.A.1.2.7 GALLSLATFN-----------SAW------------------PMIWSIAFCATSSILFCL

2.A.1.2.27 SGWGVPLTYG-----------QPS------------------VLALAMLACVTASALAWA

2.A.1.2.19 IEISKHAWLN-----------GGN------------------GLFNLFNLVNGILWLSLM

2.A.1.2.14 AYCGGMMLTL-----------GLA------------------YNYVALPAALLSFAAMSS

2.A.1.2.15 ALVGNQVSLH-----------WSM------------------SMIGYVGAVPAFAALIWS

2.A.1.2.18 ALLGSQVSLH-----------LGM------------------DKIGYVGAPLVLVALFAT

2.A.1.2.25 SAIGGVVLDQ-----------TGT------------------VVSTAWCGGSIVIIAVLF

2.A.1.2.26 AAIGGYALDN-----------IGL------------------TSPLMLSGTLMLLTALLV

2.A.1.2.11 PSTGGVIVQV-----------IGFP-----------------WLMVIIGTINIIYAPLCC

2.A.1.2.12 PSTGGAIVKA-----------IGFP-----------------WLMVITGVINIVYAPLCY

2.A.1.2.29 PSAGGAIAKA-----------IGFP-----------------WLMTIIGIIDILFAPLCF

2.A.1.2.28 PIVAGHIVHS-----------LGFE-----------------QLSLGMGLANLLYAPVLL

2.A.1.2.30 PALYGFIFYM-----------FHVELTELGPKLNSNNVPLQGAVIPGPPFLFGACIVLMS

```
2.A.1.2.9     SGVLASLSAM-----------LPQ------------------TGQGSLGLLMTLMGLLIV
2.A.1.2.22    IWLAAVVGIG-----------AWN------------------MLIGILIACSIVSLLLIM
2.A.1.2.32    GPLVAATTLG-----------HTT------------------GNLGPLWASGLLVAVALL
2.A.1.2.21    YIGGGWLFDL-----------GKS------------------AHQPELPWMMLGIIGIFT
2.A.1.2.37    LIGLLLGGLF-----------AFYL-----------------PYPAIFMIIAGIYSLFAI
2.A.1.2.5     GLLVSISPMI-----------KAI------------------GVSLVLALTEVLAIILVL
2.A.1.1.1     GATFLTMLNTL----------GNA------------------NTFWVYAALNVLFILLTL
2.A.1.1.2     GATFLTLLDSI----------GAA------------------GTFWLYTALNIAFVGITF
2.A.1.1.26    SFTFPILLAAI----------GLS------------------TTFFIFVGLGICSVLFVK
2.A.1.1.53    GLFFPTIMEAV----------GLT------------------GTFFMFAGIGVVALIFIY
2.A.1.1.55    GQFVPMMIDSF----------GLA------------------YTFWIFAVINILCFLFVV
2.A.1.1.25    SLTFLHTAEYL----------TYY------------------GAFFLYAGFAAVGLLFIY
2.A.1.1.63    AQSFLSLTEAI----------GTS------------------WTFLIFGVISVIALLFVM
2.A.1.1.66    AQTFLTIAEAA----------GTG------------------MTFLILAGIAVLAVIFVI
2.A.1.1.32    STTFPPLLDTV----------GLG------------------PAYGLYATSAAISIFFIW
2.A.1.1.35    TASFPSLAD-W----------NLS------------------GTYVIYTIFAALSIPFVL
2.A.1.1.65    TVSFPELRN------------HLG------------------LAYGFYALCAVLSFLFVS
2.A.1.1.6     AFFTPFITSA-----------INF------------------YYGYVFMGCLVAMFFYVF
2.A.1.1.67    SFFTPFITSA-----------IHF------------------YYGFVFTGCLAFSFFYVY
2.A.1.1.36    SFFTPFISGS-----------IHF------------------AYGYVFASCCVVGVLIVF
2.A.1.1.58    SFFTPFISSS-----------IDF------------------AYGYVFAGCCFAAIFVVF
2.A.1.1.19    ALITPYIV-DVGSHTSS----MGP------------------KIFFIWGGLNVVAVIVVY
2.A.1.1.8     ASTFLTML-Q-----NI----TPA------------------GTFAFFAGLSCLSTIFCY
2.A.1.1.21    SFFTPFIQAS-----------IGF------------------KYGYVFASCNLTGAIVIF
2.A.1.1.23    SFFTPFINNA-----------IGF------------------KLGYIYACINLFSSFMIF
2.A.1.1.42    SQFFLVLL-D---AFGN----NVG------------------GPFAIFGVFSALSIPFVL
```

2.A.1.1.33 TYNFNKMK-D---AFTY----TGL------------------TLGFYGGIAIVIGIPYQL

2.A.1.1.18 SVLILYQL-N--SKKWN----WGA------------------KSGFFWGVLCFCTLIWAV

2.A.1.1.20 SQVFPILM-G-----AI----GVG------------------GTFTIISGLMALGCIFVY

2.A.1.1.14 GQAFVSMLCA-----------MEY------------------GVFLFFAGWLVIMVLCAI

2.A.1.1.50 AQAFLSMLCH-----------FKF------------------GIFIFFSAWVLIMSVFVM

2.A.1.1.60 AQIFLTMLCH-----------LKF------------------GLFLVFAFFVVVMSIFVY

2.A.1.1.61 GQFFLTMLCH-----------MKF------------------GLFYFFAGMVLIMTIFIY

2.A.1.1.56 AQAFLSMLCG-----------MRS------------------GIFFFFSGWIIVMGLFAF

2.A.1.1.37 GFLFPSIQEA-----------IGA------------------YSFIIFAGICLLTAIYIY

2.A.1.1.47 GLLFPFIQKS-----------LDS------------------YCFLVFATICIAGATYFY

2.A.1.1.52 LCSYPVIQKN-----------IGG------------------YSFLPFLVVVVICWIFFF

2.A.1.1.51 AYATPYMVDEDKGN-------LGS------------------NVFFIWGGFNLACVFFAW

2.A.1.1.57 AVITPYMVDKDKGD-------LKA------------------KVFFIWGSLCACAFVYTY

2.A.1.1.28 GMCFQYVEQL-----------CGP------------------YVFIIFTVLLVLFFIFTY

2.A.1.1.29 ALCFQYIADF-----------CGP------------------YVFFLFAGVLLAFTLFTF

2.A.1.1.41 SLTFPSLLDFF----------GTG------------------SLFIGYGILCFASIWFVQ

2.A.1.1.54 ALFFPALVSGV----------GIT------------------FSFLIFAVVGVIALAFVT

2.A.1.1.24 VFPSDIIIKK-----------SPS------------------ILFIVFSVMSILTFFFIF

2.A.1.1.43 GLIFPVLKDA-----------LKN------------------YTFLVFAIITSFGAIFTL

2.A.1.1.3 SWTFPMMDKNSWLVAHF----HNG------------------FSYWIYGCMGVLAALFMW

2.A.1.1.4 NFLFKVADGSPALNQTF----NHG------------------FSYLVFAALSILGGLIVA

2.A.1.1.46 TKEFSS------VMEML----RPY------------------GAFWLTAAFCALSVLFTL

2.A.1.1.59 SLSFLDLI------GTI----GLS------------------WTFLLYGLTAVLGLGFIY

2.A.1.1.17 NLLYPITTEAISGGATGDQDKGQA------------------VVFILFGLIGLICFVLQF

2.A.1.2.1	IIYTFYFFGQRI-------------------------RALSSLTGNKALKYLPLENN---
2.A.1.2.2	IPVFFYLNGPKL-------------------------RARSKYAY---------------
2.A.1.2.6	IPVLFYLNGPKL-------------------------RARSKYAN---------------
2.A.1.2.17	IPFILYKYGPSL-------------------------RTRSSYTEE--------------
2.A.1.2.35	IPFVLHKWGLKL-------------------------RSRSKYAS---------------
2.A.1.2.16	VPLLFLKYGESI-------------------------RKKSKYAYAA-------------
2.A.1.2.40	GILYLLIFQRKY-------------------------TAKEF------------------
2.A.1.2.31	LLFILLRKGKEL-------------------------AFKRKKQELGVN-----------
2.A.1.2.3	VSRVKGSRGQGEHD----------------VVALQSAGSTSNPNR---------------
2.A.1.2.4	ALRRGLWSGAGQ-------------------------RADR-------------------
2.A.1.2.38	LLLKYRLNKYSGVP--
2.A.1.2.39	LLARSRARDAAP--
2.A.1.2.41	RAHIANTAKRPA-------------------------GETTGDAPAALVETAG-------
2.A.1.2.8	VWKEKKNDAAALN---
2.A.1.2.10	IEKQHRAKLKEQNM--
2.A.1.2.24	IFGRETLPTAKVEQ----------------------V-----------------------
2.A.1.2.20	WNSLRRRRIPQVSN--
2.A.1.2.34	LIQFRTLLKVKEI---
2.A.1.2.7	YASRPKKR--
2.A.1.2.27	WLQHLSRISAKVQA----------------------R-----------------------
2.A.1.2.19	VIFLKDKQMGNSHE----------------------G-----------------------
2.A.1.2.14	LLLYGRYKRQQ--------------------------AADTPVLAKPLG-----------
2.A.1.2.15	IIIFRRWPVTL--------------------------EEQTQ------------------
2.A.1.2.18	LLSVYRSVRLV--------------------------HSRV-------------------
2.A.1.2.25	AFISLTRPVQT--------------------------AKKSSL-----------------
2.A.1.2.26	TAKVKMKKS---

2.A.1.2.11	FLQNPPAKEEKRAIL-----------SQECPTETQMYTFQKPTKAFPLGENSD-DPSSGE
2.A.1.2.12	YLRSPPAKEEKLAIL-----------SQDCPMETRMYATQKPTKEFPLGEDSDEEPDHEE
2.A.1.2.29	FLRSPPAKEEKMAIL-----------MDHNCPIKTKMYTQNNIQSYPIGEDEESESD---
2.A.1.2.28	LLRNVGLLTRSRSERDVLLDEPPQGLYDAVRLRERPVSGQDGEPRSPPGPFDECEDDYNY
2.A.1.2.30	FLVALFIPEYSKASGVQKHSNSSSGSLTNTPERGSDEDIEPLLQDSSIWELSSFE-EPGN
2.A.1.2.9	LCWLPLATRMS--------------------------HQGQPV-----------------
2.A.1.2.22	FVAPGRPVAAH--------------------------EEIHHHA----------------
2.A.1.2.32	VAFPFRTVITT--------------------------AAPADATR---------------
2.A.1.2.21	FLALGWQFSQK--------------------------RAARRLLERDA------------
2.A.1.2.37	FVYGYDRLNVRQMT----------------DFSNSYFNMKS-------------------
2.A.1.2.5	VAVNRHQKTKLN--
2.A.1.1.1	WLVPETKHVSLE-------------------------HIERNLMKGRKLREIGAHD----
2.A.1.1.2	WLIPETKNVTLE-------------------------HIERKLMAGEKLRNIGV------
2.A.1.1.26	RFLPETKGLSLE-------------------------QLEENFRAYDHSGAKKDSGAEVI
2.A.1.1.53	TQVPETRGRTLE-------------------------EIDEDVTSGVIFNKDIRKGKVH-
2.A.1.1.55	TICPETKNKSLE-------------------------EIEKLWIK---------------
2.A.1.1.25	GCLPETKGKKLE-------------------------EIESLFDNRLCTCGTSDSD----
2.A.1.1.63	VCVPETKGMPME-------------------------EIEKMLERRSMEFKFWKKK----
2.A.1.1.66	VFVPETQGLTFS-------------------------EVEQIWKERAYGNISGWGS----
2.A.1.1.32	FFVKETKGKTLE-------------------------QM---------------------
2.A.1.1.35	KFVKETKGKALE-------------------------EMG--------------------
2.A.1.1.65	KWVEETRGKNLE-------------------------DMHAEALGH--------------
2.A.1.1.6	FFVPETKGLSLE-------------------------EIQELWEEGVLPWKSEGWI----
2.A.1.1.67	FFVYETKGLSLE-------------------------EVDEMYASGVLPLKSASWV----
2.A.1.1.36	FFVNETQGRTLE-------------------------EVDTMYVLHVVPWKSASWVPDES
2.A.1.1.58	FFVNETQGRTLE-------------------------EVDTMYVLHVKPWQSASWVPPEG

2.A.1.1.19	FAVYETRGLTLE-------------------------EIDELFRKAPNSVISSKWNKKIR
2.A.1.1.8	FCYPELSGLELE-------------------------EVQTILKDGFNIKASKAL-----
2.A.1.1.21	LFAKETKGLTLE-------------------------EINELYMSVIKPWESGNFK----
2.A.1.1.23	FLAKETKGLTLE-------------------------EVNDLYMSNIKPWESYKYVREIE
2.A.1.1.42	RLVPETKGKSLE-------------------------EIEKEMTKR--------------
2.A.1.1.33	LFMPETKDKTLE-------------------------EIDDIFEMPTRQLVKQNLKNLKD
2.A.1.1.18	VDLPETAGKTFV-------------------------EINELFKLGVSARKFKSTKV---
2.A.1.1.20	FFAVETKGLTLE-------------------------QIDNMFRKRAGLPPRFHEE----
2.A.1.1.14	FLLPETKGVPIE-------------------------RV-QALYARHWFWNRVMGPAAAE
2.A.1.1.50	FLLPETKNIPIE-------------------------EMTERVWKKHWFWARFMDDHNDH
2.A.1.1.60	IFLPETKGIPIE-------------------------EM-GQVWRSHWYWSRFVEDGEYG
2.A.1.1.61	FLLPETKGVPIE-------------------------EM-GKVWKEHRYWGKYSNNDDGD
2.A.1.1.56	FFIPETKGIAID-------------------------DMRESVWKPHWFWKRYMLPEDDH
2.A.1.1.37	VVIPETKGKTFV-------------------------EINRIFAKRNRVKLPEEKEE---
2.A.1.1.47	FVLPETKNRTHA-------------------------EISQAFAKRNKAQPPEVKAD---
2.A.1.1.52	LFMPETKNRTFD-------------------------EVARDLAFGNIVVGKRTTALEDR
2.A.1.1.51	YFIYETKGLSLE-------------------------QVDELYEHVSKAWKSKGFV----
2.A.1.1.57	FLIPETKGLTLE-------------------------QVDKMMEETTPRTSAKWTP----
2.A.1.1.28	FKVPETKGRTFD-------------------------EIASGFRQGGASQSDKTPEELFH
2.A.1.1.29	FKVPETKGKSFE-------------------------EIAAEFQKKSGSAHRPKAAVEMK
2.A.1.1.41	KKVFETRNRSLE-------------------------DIEATLRAKTGEDAAELSTTK--
2.A.1.1.54	KFVPETRGRSLE-------------------------ELDHAAFTGQIFKKA--------
2.A.1.1.24	FFIKETKGGEIG-------------------------TSPYITMEERQKHMTKSVV----
2.A.1.1.43	LFVPETKGRTLE-------------------------EIHNENSKDTKNLDSQMKS----
2.A.1.1.3	KFVPETKGKTLE-------------------------ELEALWEPETKKTQQTATL----
2.A.1.1.4	RFVPETKGRSLD-------------------------EIEEMWRSQK-------------

2.A.1.1.46 TVVPETKGRTLE-------------------------QVTAHFEGR--------------

2.A.1.1.59 LFVPETKGQSLA-------------------------EIDQQFQKRRFTLSFGHR-----

2.A.1.1.17 FYLYPYDANQDH-------------------------ENDHGTEPVERILSPVDVP----

2.A.1.2.1 --

2.A.1.2.2 --

2.A.1.2.6 --

2.A.1.2.17 --

2.A.1.2.35 --

2.A.1.2.16 --

2.A.1.2.40 --

2.A.1.2.31 --

2.A.1.2.3 --

2.A.1.2.4 --

2.A.1.2.38 --

2.A.1.2.39 --

2.A.1.2.41 --

2.A.1.2.8 --

2.A.1.2.10 --

2.A.1.2.24 --

2.A.1.2.20 --

2.A.1.2.34 --

2.A.1.2.7 --

2.A.1.2.27 --

2.A.1.2.19 --

```
2.A.1.2.14      ------------------------------------------------------------
2.A.1.2.15      ------------------------------------------------------------
2.A.1.2.18      ------------------------------------------------------------
2.A.1.2.25      ------------------------------------------------------------
2.A.1.2.26      ------------------------------------------------------------
2.A.1.2.11      ------------------------------------------------------------
2.A.1.2.12      ------------------------------------------------------------
2.A.1.2.29      ------------------------------------------------------------
2.A.1.2.28      YYTRS-------------------------------------------------------
2.A.1.2.30      QCTEL-------------------------------------------------------
2.A.1.2.9       ------------------------------------------------------------
2.A.1.2.22      ------------------------------------------------------------
2.A.1.2.32      ------------------------------------------------------------
2.A.1.2.21      ------------------------------------------------------------
2.A.1.2.37      ------------------------------------------------------------
2.A.1.2.5       ------------------------------------------------------------
2.A.1.1.1       ------------------------------------------------------------
2.A.1.1.2       ------------------------------------------------------------
2.A.1.1.26      G-----------------------------------------------------------
2.A.1.1.53      ------------------------------------------------------------
2.A.1.1.55      ------------------------------------------------------------
2.A.1.1.25      ------------------------------------------EGRYIEYIRVKGSNYHLS
2.A.1.1.63      ------------------------------------------SKLVEKQNQSA-------
2.A.1.1.66      ------------------------------------------SSDSNNMEGLLEQGSQS-
2.A.1.1.32      ------------------------------------------------------------
2.A.1.1.35      ------------------------------------------------------------
```

```
2.A.1.1.65    ------------------------------------------------------------

2.A.1.1.6     -----------------------------------------PSSRRGNNYDLEDLQHDDK

2.A.1.1.67    -----------------------------------------PPNLEHMAHSAGYAGADKA

2.A.1.1.36    IVRDL-----------------------------------HPGSDANKTEGLGQAEHGEE

2.A.1.1.58    IVQDMPAPL----------------PLPSRRVRLRWLSTPSPLSSASKPLALARTHFASC

2.A.1.1.19    KRCLAFPISQQIEMKTNIKNAGKLDNNNSPIVQDDSHNIIDVDGFLENQIQSNDHMIAAD

2.A.1.1.8     --------------------------------------------AKKRKQQVARVHELKY

2.A.1.1.21    -----------------------LNYSEQKKVEKEKSRKGGARGESVEYVERASNTDSSP

2.A.1.1.23    SHRIHFSKEEEKREREKSKGIRGQEEEFIENADEDNNDSSSSSGSVVSAVKPRRSAVSND

2.A.1.1.42    ------------------------------------------------------------

2.A.1.1.33    YVSGNRKSI---------------------------------------------------

2.A.1.1.18    -----------------------------------------DPFVVKTPPKDVSHNDPKG

2.A.1.1.20    ------------------------------------------GESGESGAGYREDGDLGR

2.A.1.1.14    VIA--------------------------------------EDEKRVAAASAIIKEEELS

2.A.1.1.50    EFV--------------------------------------NGEKSNGKSNGFDPSTRL-

2.A.1.1.60    NAL--------------------------------------EMGKNSNQAGTKHV-----

2.A.1.1.61    DVD--------------------------------------DDAYF--------------

2.A.1.1.56    HDI--------------------------------------EKRNA--------------

2.A.1.1.37    -----------------------------------------TIDAGPPTASPAKETSF--

2.A.1.1.47    -----------------------------------------SAMTEEKANSQTEPDSSST

2.A.1.1.52    NLT--------------------------------------VFTKQGNNEGPASESLLYP

2.A.1.1.51    ------------------------------------------PSKHSFREQVDQQMDSKT

2.A.1.1.57    ------------------------------------------HGTFTAEMGLTANAVAEK

2.A.1.1.28    PLGADSQV----------------------------------------------------

2.A.1.1.29    FLGATETV----------------------------------------------------

2.A.1.1.41    ------------------------------------------------------------
```

2.A.1.1.54 --

2.A.1.1.24 --

2.A.1.1.43 --

2.A.1.1.3 --

2.A.1.1.4 --

2.A.1.1.46 --

2.A.1.1.59 --QNSTGIPYSRIEIS

2.A.1.1.17 --TPRN--------------

2.A.1.2.1 --

2.A.1.2.2 --

2.A.1.2.6 --

2.A.1.2.17 --

2.A.1.2.35 --

2.A.1.2.16 --

2.A.1.2.40 --

2.A.1.2.31 --

2.A.1.2.3 --

2.A.1.2.4 --

2.A.1.2.38 --

2.A.1.2.39 --

2.A.1.2.41 --

2.A.1.2.8 --

2.A.1.2.10 --

2.A.1.2.24 --

2.A.1.2.20 --
2.A.1.2.34 --
2.A.1.2.7 --
2.A.1.2.27 --
2.A.1.2.19 --
2.A.1.2.14 --
2.A.1.2.15 --
2.A.1.2.18 --
2.A.1.2.25 --
2.A.1.2.26 --
2.A.1.2.11 --
2.A.1.2.12 --
2.A.1.2.29 --
2.A.1.2.28 --
2.A.1.2.30 --
2.A.1.2.9 --
2.A.1.2.22 --
2.A.1.2.32 --
2.A.1.2.21 --
2.A.1.2.37 --
2.A.1.2.5 --
2.A.1.1.1 --
2.A.1.1.2 --
2.A.1.1.26 --
2.A.1.1.53 --
2.A.1.1.55 --

2.A.1.1.25	DNDASDVE--
2.A.1.1.63	--
2.A.1.1.66	--
2.A.1.1.32	--
2.A.1.1.35	--
2.A.1.1.65	--
2.A.1.1.6	PWYKAMLE--
2.A.1.1.67	TDEQV---
2.A.1.1.36	SRPEPVEIRE--
2.A.1.1.58	SCTLNLHLKL--
2.A.1.1.19	KGSGSLVNIIDTAPLTSTEFKPVEHPPVNYVDLGNGLGLNTYNRGPPSIISDSTDEFYEE
2.A.1.1.8	EPTQEIIEDI--
2.A.1.1.21	QYSSHEEDYA--
2.A.1.1.23	RFSEDSHPTYI---
2.A.1.1.42	--
2.A.1.1.33	--
2.A.1.1.18	DIEASIAEE---
2.A.1.1.20	LATEDVCD--
2.A.1.1.14	KAMK--
2.A.1.1.50	--
2.A.1.1.60	--
2.A.1.1.61	--
2.A.1.1.56	--
2.A.1.1.37	--
2.A.1.1.47	LDSYGQNKIV--
2.A.1.1.52	RSDNDKGMYA--

2.A.1.1.51 EAIMSEEASV--

2.A.1.1.57 ATAVHQEV--

2.A.1.1.28 --

2.A.1.1.29 --

2.A.1.1.41 --

2.A.1.1.54 --

2.A.1.1.24 --

2.A.1.1.43 --

2.A.1.1.3 --

2.A.1.1.4 --

2.A.1.1.46 --

2.A.1.1.59 AAS---

2.A.1.1.17 --

2.A.1.2.1 --

2.A.1.2.2 --

2.A.1.2.6 --

2.A.1.2.17 --

2.A.1.2.35 --

2.A.1.2.16 --

2.A.1.2.40 --

2.A.1.2.31 --

2.A.1.2.3 --

2.A.1.2.4 --

2.A.1.2.38 --

2.A.1.2.39 --

2.A.1.2.41 --

2.A.1.2.8 --

2.A.1.2.10 --

2.A.1.2.24 --

2.A.1.2.20 --

2.A.1.2.34 --

2.A.1.2.7 --

2.A.1.2.27 --

2.A.1.2.19 --

2.A.1.2.14 --

2.A.1.2.15 --

2.A.1.2.18 --

2.A.1.2.25 --

2.A.1.2.26 --

2.A.1.2.11 --

2.A.1.2.12 --

2.A.1.2.29 --

2.A.1.2.28 --

2.A.1.2.30 --

2.A.1.2.9 --

2.A.1.2.22 --

2.A.1.2.32 --

2.A.1.2.21 --

2.A.1.2.37 --

2.A.1.2.5 --

2.A.1.1.1	--
2.A.1.1.2	--
2.A.1.1.26	--
2.A.1.1.53	--
2.A.1.1.55	--
2.A.1.1.25	--
2.A.1.1.63	--
2.A.1.1.66	--
2.A.1.1.32	--
2.A.1.1.35	--
2.A.1.1.65	--
2.A.1.1.6	--
2.A.1.1.67	--
2.A.1.1.36	--
2.A.1.1.58	--
2.A.1.1.19	NDSSYYNNNTERNGANSVNTYMAQLINSSSTTSNDTSFSPSHNSNARTSSNWTSDLASKH
2.A.1.1.8	--
2.A.1.1.21	--
2.A.1.1.23	--
2.A.1.1.42	--
2.A.1.1.33	--
2.A.1.1.18	--
2.A.1.1.20	LSSLGNRVVSFAKAEDAFTEVAMPDRHAVSNKFEERATSSSSDPQSLENQDEVRQAAIKA
2.A.1.1.14	--
2.A.1.1.50	--
2.A.1.1.60	--

2.A.1.1.61 ---

2.A.1.1.56 ---

2.A.1.1.37 ---

2.A.1.1.47 ---

2.A.1.1.52 ---

2.A.1.1.51 ---

2.A.1.1.57 ---

2.A.1.1.28 ---

2.A.1.1.29 ---

2.A.1.1.41 ---

2.A.1.1.54 ---

2.A.1.1.24 ---

2.A.1.1.43 ---

2.A.1.1.3 ---

2.A.1.1.4 ---

2.A.1.1.46 ---

2.A.1.1.59 ---

2.A.1.1.17 ---

2.A.1.2.1 -------

2.A.1.2.2 -------

2.A.1.2.6 -------

2.A.1.2.17 -------

2.A.1.2.35 -------

2.A.1.2.16 -------

2.A.1.2.40	-------
2.A.1.2.31	-------
2.A.1.2.3	-------
2.A.1.2.4	-------
2.A.1.2.38	-------
2.A.1.2.39	-------
2.A.1.2.41	-------
2.A.1.2.8	-------
2.A.1.2.10	-------
2.A.1.2.24	-------
2.A.1.2.20	-------
2.A.1.2.34	-------
2.A.1.2.7	-------
2.A.1.2.27	-------
2.A.1.2.19	-------
2.A.1.2.14	-------
2.A.1.2.15	-------
2.A.1.2.18	-------
2.A.1.2.25	-------
2.A.1.2.26	-------
2.A.1.2.11	-------
2.A.1.2.12	-------
2.A.1.2.29	-------
2.A.1.2.28	-------
2.A.1.2.30	-------
2.A.1.2.9	-------

2.A.1.2.22	-------
2.A.1.2.32	-------
2.A.1.2.21	-------
2.A.1.2.37	-------
2.A.1.2.5	-------
2.A.1.1.1	-------
2.A.1.1.2	-------
2.A.1.1.26	-------
2.A.1.1.53	-------
2.A.1.1.55	-------
2.A.1.1.25	-------
2.A.1.1.63	-------
2.A.1.1.66	-------
2.A.1.1.32	-------
2.A.1.1.35	-------
2.A.1.1.65	-------
2.A.1.1.6	-------
2.A.1.1.67	-------
2.A.1.1.36	-------
2.A.1.1.58	-------
2.A.1.1.19	SQYTSPQ
2.A.1.1.8	-------
2.A.1.1.21	-------
2.A.1.1.23	-------
2.A.1.1.42	-------
2.A.1.1.33	-------

2.A.1.1.18	-------
2.A.1.1.20	APHEPK-
2.A.1.1.14	-------
2.A.1.1.50	-------
2.A.1.1.60	-------
2.A.1.1.61	-------
2.A.1.1.56	-------
2.A.1.1.37	-------
2.A.1.1.47	-------
2.A.1.1.52	-------
2.A.1.1.51	-------
2.A.1.1.57	-------
2.A.1.1.28	-------
2.A.1.1.29	-------
2.A.1.1.41	-------
2.A.1.1.54	-------
2.A.1.1.24	-------
2.A.1.1.43	-------
2.A.1.1.3	-------
2.A.1.1.4	-------
2.A.1.1.46	-------
2.A.1.1.59	-------
2.A.1.1.17	-------
