# Supplementary material for: Employing Information Theoretic Measures and Mutagenesis to Identify Residues Critical for Drug-Proton Antiport Function in Mdr1p of Candida albicans
Source: PLoS One. 2010 Jun 10;5(6):e11041. doi: 10.1371/journal.pone.0011041 (PMC2883579; doi:10.1371/journal.pone.0011041)
Supplement: Supplementary Data S2 — RE across DHA1 and SP families, CRE and CRES scores for the entire MSA. (1.32 MB DOC) [file pone.0011041.s002.doc]

| **Alignment Position** | **RE of DHA1** | **MFA in DHA1** | **RE of SP** | **MFA in SP** | **CRE** | **CaMdr1p Residue** | **CaMdr1p Position** | **RE*CRE** |
| --- | --- | --- | --- | --- | --- | --- | --- | --- |
| 1 | 0.151171993 | gap | 0 | gap | 0.071545813 | - | - | 0.010815723 |
| 2 | 0.054850402 | gap | 0 | gap | 0.017997093 | M | 1 | 0.000987148 |
| 3 | 0.040142221 | gap | 0 | gap | 0.021956077 | H | 2 | 0.000881366 |
| 4 | 0.049608915 | gap | 0 | gap | 0.025748127 | Y | 3 | 0.001277337 |
| 5 | 0.10418779 | gap | 0 | gap | 0.038432306 | R | 4 | 0.004004177 |
| 6 | 0.183814457 | gap | 0 | gap | 0.081804417 | F | 5 | 0.015036835 |
| 7 | 0.124657564 | gap | 0 | gap | 0.049803371 | L | 6 | 0.006208367 |
| 8 | 0.19898703 | gap | 0 | gap | 0.086293366 | R | 7 | 0.017171261 |
| 9 | 0.103082307 | gap | 0 | gap | 0.051159308 | D | 8 | 0.005273619 |
| 10 | 0.102478097 | gap | 0 | gap | 0.042132555 | S | 9 | 0.004317664 |
| 11 | 0.124694504 | gap | 0 | gap | 0.054709733 | F | 10 | 0.006822003 |
| 12 | 0.081375862 | gap | 0 | gap | 0.03184942 | V | 11 | 0.002591774 |
| 13 | 0.101220186 | gap | 0 | gap | 0.030354149 | G | 12 | 0.003072453 |
| 14 | 0.095601701 | gap | 0 | gap | 0.039624611 | R | 13 | 0.00378818 |
| 15 | 0.300848446 | gap | 0 | gap | 0.131358611 | V | 14 | 0.039519034 |
| 16 | 0.084242509 | gap | 0 | gap | 0.028101865 | T | 15 | 0.002367372 |
| 17 | 0.189401439 | gap | 0 | gap | 0.080003222 | Y | 16 | 0.015152725 |
| 18 | 0.069892111 | gap | 0 | gap | 0.03525053 | H | 17 | 0.002463734 |
| 19 | 0.116427125 | gap | 0 | gap | 0.036327268 | L | 18 | 0.004229479 |
| 20 | 0.379357125 | gap | 0 | gap | 0.151922238 | S | 19 | 0.057632783 |
| 21 | 0.064275406 | gap | 0 | gap | 0.025299269 | - | - | 0.001626121 |
| 22 | 0.053464815 | gap | 0 | gap | 0.018957677 | - | - | 0.001013569 |
| 23 | 0.071870148 | gap | 0 | gap | 0.030439766 | K | 20 | 0.00218771 |
| 24 | 0.079615032 | gap | 0 | gap | 0.034648848 | H | 21 | 0.002758569 |
| 25 | 0.235020459 | gap | 0.013868589 | gap | 0.190643724 | K | 22 | 0.044805175 |
| 26 | 0.093396415 | gap | 0.025968622 | gap | 0.143322368 | Y | 23 | 0.013385795 |
| 27 | 0.091996888 | gap | 0.292862584 | gap | 0.456495189 | F | 24 | 0.041996137 |
| 28 | 0.098204438 | gap | 0.129959424 | gap | 0.210631828 | A | 25 | 0.02068498 |
| 29 | 0.073714194 | gap | 0.118108033 | gap | 0.147348749 | H | 26 | 0.010861694 |
| 30 | 0.130830806 | gap | 0.165797686 | gap | 0.30861335 | P | 27 | 0.040376133 |
| 31 | 0.215075171 | gap | 0.149675203 | gap | 0.216053861 | E | 28 | 0.046467821 |
| 32 | 0.102855297 | gap | 0.111362603 | gap | 0.127678035 | E | 29 | 0.013132362 |
| 33 | 0.170516367 | gap | 0.145595763 | gap | 0.333813749 | A | 30 | 0.056920708 |
| 34 | 0.086686034 | gap | 0.124034131 | gap | 0.221796957 | K | 31 | 0.019226699 |
| 35 | 0.162392826 | gap | 0.175712898 | gap | 0.393825444 | N | 32 | 0.063954427 |
| 36 | 0.133024795 | gap | 0.116673453 | gap | 0.27216023 | Y | 33 | 0.036204059 |
| 37 | 0.10592276 | gap | 0.204374036 | gap | 0.259602833 | I | 34 | 0.027497849 |
| 38 | 0.172082919 | gap | 0.128406298 | gap | 0.407372183 | I | 35 | 0.070101794 |
| 39 | 0.075964471 | gap | 0.184581894 | gap | 0.194053659 | P | 36 | 0.014741184 |
| 40 | 0.105071404 | gap | 0.166066677 | gap | 0.192813018 | E | 37 | 0.020259134 |
| 41 | 0.077375127 | gap | 0.209041916 | gap | 0.160813996 | K | 38 | 0.012443003 |
| 42 | 0.066289433 | gap | 0.265911122 | gap | 0.393613615 | Y | 39 | 0.026092423 |
| 43 | 0.066947071 | gap | 0.1877441 | gap | 0.291220946 | L | 40 | 0.019496389 |
| 44 | 0.054417111 | gap | 0.33816856 | gap | 0.407679733 | A | 41 | 0.022184753 |
| 45 | 0.108663458 | gap | 0.050311301 | gap | 0.163932185 | D | 42 | 0.017813438 |
| 46 | 0.035466573 | gap | 0.038684181 | gap | 0.039179088 | Y | 43 | 0.001389548 |
| 47 | 0.049246895 | gap | 0.083642003 | gap | 0.114275089 | K | 44 | 0.005627693 |
| 48 | 0.047784867 | gap | 0.031444088 | gap | 0.06104584 | P | 45 | 0.002917067 |
| 49 | 0.049864974 | gap | 0.030038884 | gap | 0.075973931 | T | 46 | 0.003788438 |
| 50 | 0.085855029 | gap | 0.038886973 | gap | 0.106314832 | L | 47 | 0.009127663 |
| 51 | 0.033581409 | gap | 0.035590772 | gap | 0.042279728 | A | 48 | 0.001419813 |
| 52 | 0.035622439 | gap | 0.027865861 | gap | 0.055293866 | D | 49 | 0.001969702 |
| 53 | 0.036141515 | gap | 0.035590772 | gap | 0.041873506 | D | 50 | 0.001513372 |
| 54 | 0.075981788 | gap | 0.029906811 | gap | 0.028979527 | T | 51 | 0.002201916 |
| 55 | 0.053349743 | gap | 0.047068289 | gap | 0.079881634 | S | 52 | 0.004261665 |
| 56 | 0.039486421 | gap | 0.036885985 | gap | 0.037177623 | I | 53 | 0.001468011 |
| 57 | 0.042031059 | gap | 0.034536071 | gap | 0.051706007 | N | 54 | 0.002173258 |
| 58 | 0.042797977 | gap | 0.028820323 | gap | 0.066488039 | F | 55 | 0.002845554 |
| 59 | 0.041737285 | gap | 0.031396364 | gap | 0.041943229 | E | 56 | 0.001750597 |
| 60 | 0.069896319 | gap | 0.060672779 | gap | 0.073470235 | K | 57 | 0.005135299 |
| 61 | 0.060724762 | gap | 0.072568514 | gap | 0.118325145 | E | 58 | 0.007185266 |
| 62 | 0.102426892 | gap | 0.082394261 | gap | 0.086009759 | E | 59 | 0.008809712 |
| 63 | 0.051211464 | gap | 0.109721659 | gap | 0.171078462 | I | 60 | 0.008761179 |
| 64 | 0.121449883 | gap | 0.217760148 | gap | 0.32731493 | D | 61 | 0.03975236 |
| 65 | 0.092364562 | gap | 0.372484492 | gap | 0.236755298 | N | 62 | 0.021867799 |
| 66 | 0.072619828 | gap | 0.27882101 | gap | 0.328473102 | Q | 63 | 0.02385366 |
| 67 | 0.057914653 | gap | 0.312474123 | gap | 0.588852306 | G | 64 | 0.034103177 |
| 68 | 0.098379384 | gap | 0.228368577 | gap | 0.182225794 | E | 65 | 0.017927261 |
| 69 | 0.081830169 | gap | 0.335356845 | gap | 0.394803794 | P | 66 | 0.032306861 |
| 70 | 0.094225878 | gap | 0.378431847 | gap | 0.187034828 | N | 67 | 0.017623521 |
| 71 | 0.11151485 | gap | 0.216867112 | gap | 0.3451132 | S | 68 | 0.038485247 |
| 72 | 0.142507942 | gap | 0.436432773 | gap | 0.275270617 | S | 69 | 0.039228249 |
| 73 | 0.093616666 | gap | 0.3594254 | gap | 0.418339988 | Q | 70 | 0.039163595 |
| 74 | 0.205869946 | gap | 0.306843491 | S | 0.294729292 | S | 71 | 0.060675904 |
| 75 | 0.197505597 | gap | 0.337042553 | gap | 0.449265569 | S | 72 | 0.088732465 |
| 76 | 0.111614926 | gap | 0.480959328 | G | 0.179058732 | S | 73 | 0.019985627 |
| 77 | 0.080143545 | gap | 0.188865762 | gap | 0.338495581 | S | 74 | 0.027128236 |
| 78 | 0.139976362 | gap | 0.212025704 | gap | 0.193831076 | N | 75 | 0.027131769 |
| 79 | 0.173968956 | gap | 0.404993039 | T | 0.457451625 | N | 76 | 0.079582382 |
| 80 | 0.144370751 | gap | 0.332598188 | gap | 0.29325721 | T | 77 | 0.042337764 |
| 81 | 0.123447645 | gap | 0.161483308 | gap | 0.220061685 | I | 78 | 0.027166097 |
| 82 | 0.120062325 | gap | 0.118883658 | gap | 0.125812574 | V | 79 | 0.01510535 |
| 83 | 0.213591484 | gap | 0.041303852 | gap | 0.16252847 | D | 80 | 0.034714697 |
| 84 | 0.113606637 | gap | 0.06864097 | gap | 0.170506458 | N | 81 | 0.019370665 |
| 85 | 0.224911492 | gap | 0.058237844 | gap | 0.10177143 | N | 82 | 0.022889564 |
| 86 | 0.13864759 | gap | 0.067551541 | gap | 0.154989093 | N | 83 | 0.021488864 |
| 87 | 0.168847268 | gap | 0.079487798 | gap | 0.194244584 | N | 84 | 0.032797667 |
| 88 | 0.13146622 | gap | 0.096782616 | gap | 0.218231379 | N | 85 | 0.028690054 |
| 89 | 0.028062569 | gap | 0.06277878 | gap | 0.121983651 | - | - | 0.003423175 |
| 90 | 0.031465247 | gap | 0.045683379 | gap | 0.102532021 | - | - | 0.003226195 |
| 91 | 0.02311695 | gap | 0.0888717 | gap | 0.056734246 | - | - | 0.001311523 |
| 92 | 0.040345839 | gap | 0.067280942 | gap | 0.166532976 | - | - | 0.006718913 |
| 93 | 0.024468577 | gap | 0.071568284 | gap | 0.0469454 | - | - | 0.001148687 |
| 94 | 0.027536193 | gap | 0.075366717 | gap | 0.044897988 | - | - | 0.00123632 |
| 95 | 0.024262116 | gap | 0.105129935 | gap | 0.090745061 | - | - | 0.002201667 |
| 96 | 0.02679252 | gap | 0.061765671 | gap | 0.049678183 | - | - | 0.001331004 |
| 97 | 0.037898557 | gap | 0.089512889 | gap | 0.159214655 | - | - | 0.006034006 |
| 98 | 0.03132795 | gap | 0.044125754 | gap | 0.035569767 | - | - | 0.001114328 |
| 99 | 0.029318564 | gap | 0.081350365 | gap | 0.098973134 | - | - | 0.00290175 |
| 100 | 0.027536193 | gap | 0.043462043 | gap | 0.085813246 | - | - | 0.00236297 |
| 101 | 0.02679252 | gap | 0.041664833 | gap | 0.06804405 | - | - | 0.001823072 |
| 102 | 0.027536193 | gap | 0.034983736 | gap | 0.057447539 | - | - | 0.001581887 |
| 103 | 0.03132795 | gap | 0.046499719 | gap | 0.066770869 | - | - | 0.002091794 |
| 104 | 0.030068916 | gap | 0.035617717 | gap | 0.06748493 | - | - | 0.002029199 |
| 105 | 0.02311695 | gap | 0.023530601 | gap | 0.031648261 | - | - | 0.000731611 |
| 106 | 0.024262116 | gap | 0.018805466 | gap | 0.047603907 | - | - | 0.001154971 |
| 107 | 0.034244767 | gap | 0.029460485 | gap | 4.43E-06 | - | - | 1.52E-07 |
| 108 | 0.026434124 | gap | 0.026864093 | gap | 0.040849445 | - | - | 0.001079819 |
| 109 | 0.037898557 | gap | 0.026864093 | gap | 0.053718298 | - | - | 0.002035846 |
| 110 | 0.021871039 | gap | 0.047889969 | gap | 0.089043041 | - | - | 0.001947464 |
| 111 | 0.040345839 | gap | 0.0546561 | gap | 0.111844511 | - | - | 0.004512461 |
| 112 | 0.040345839 | gap | 0.047237423 | gap | 0.109311766 | - | - | 0.004410275 |
| 113 | 0.03132795 | gap | 0.065865734 | gap | 0.130075071 | - | - | 0.004074985 |
| 114 | 0.029318564 | gap | 0.051239005 | gap | 0.086104395 | - | - | 0.002524457 |
| 115 | 0.037898557 | gap | 0.083258006 | gap | 0.154838193 | - | - | 0.005868144 |
| 116 | 0.030068916 | gap | 0.074394949 | gap | 0.145119071 | - | - | 0.004363573 |
| 117 | 0.079937676 | gap | 0.049462207 | gap | 0.110172959 | N | 86 | 0.00880697 |
| 118 | 0.099674178 | gap | 0.055057383 | gap | 0.122597014 | D | 87 | 0.012219757 |
| 119 | 0.126293558 | gap | 0.072344589 | gap | 0.07313314 | N | 88 | 0.009236244 |
| 120 | 0.164066257 | gap | 0.097832329 | gap | 0.213846778 | D | 89 | 0.035085041 |
| 121 | 0.129566894 | gap | 0.054352411 | gap | 0.076218488 | V | 90 | 0.009875393 |
| 122 | 0.140859196 | gap | 0.05888253 | gap | 0.16650777 | D | 91 | 0.023454151 |
| 123 | 0.144691972 | gap | 0.079679541 | gap | 0.077620739 | G | 92 | 0.011231098 |
| 124 | 0.1156011 | gap | 0.071263472 | gap | 0.103620736 | D | 93 | 0.011978671 |
| 125 | 0.092909485 | gap | 0.082051872 | gap | 0.14338218 | K | 94 | 0.013321565 |
| 126 | 0.111646119 | gap | 0.070077114 | gap | 0.227899944 | I | 95 | 0.025444144 |
| 127 | 0.132535618 | gap | 0.089766643 | gap | 0.082338519 | V | 96 | 0.010912787 |
| 128 | 0.306535051 | gap | 0.069447859 | gap | 0.298070743 | V | 97 | 0.09136913 |
| 129 | 0.135473919 | gap | 0.179022538 | gap | 0.331984296 | T | 98 | 0.044975214 |
| 130 | 0.115503849 | gap | 0.07030915 | gap | 0.218494371 | W | 99 | 0.025236941 |
| 131 | 0.189982717 | gap | 0.063986923 | gap | 0.092469506 | D | 100 | 0.017567608 |
| 132 | 0.175060789 | gap | 0.103781518 | gap | 0.075559967 | G | 101 | 0.013227587 |
| 133 | 0.182050055 | gap | 0.086440222 | gap | 0.20410805 | D | 102 | 0.037157882 |
| 134 | 0.18063066 | gap | 0.083534108 | gap | 0.170793156 | D | 103 | 0.03085048 |
| 135 | 0.32919806 | gap | 0.116707347 | gap | 0.454821333 | D | 104 | 0.149726301 |
| 136 | 0.249658829 | gap | 0.084958121 | gap | 0.274534815 | P | 105 | 0.06854004 |
| 137 | 0.218971434 | gap | 0.103095181 | gap | 0.1473454 | E | 106 | 0.032264434 |
| 138 | 0.30425851 | gap | 0.103319471 | gap | 0.300722954 | N | 107 | 0.091497518 |
| 139 | 0.277772786 | gap | 0.161148863 | gap | 0.3852175 | P | 108 | 0.107002938 |
| 140 | 0.236331354 | gap | 0.110603633 | gap | 0.258424439 | Q | 109 | 0.061073798 |
| 141 | 0.371276493 | gap | 0.151964042 | gap | 0.230313302 | N | 110 | 0.085509915 |
| 142 | 0.336510851 | W | 0.1647585 | gap | 0.556228177 | W | 111 | 0.187176817 |
| 143 | 0.416138163 | S | 0.155312705 | gap | 0.264712827 | P | 112 | 0.11015711 |
| 144 | 0.346605913 | R | 0.23935668 | gap | 0.491030154 | T | 113 | 0.170193955 |
| 145 | 0.229015757 | gap | 0.209900898 | gap | 0.367607287 | L | 114 | 0.084187861 |
| 146 | 0.336210341 | gap | 0.581271183 | F | 1.283681624 | Q | 115 | 0.431587036 |
| 147 | 0.43092701 | R | 0.950798948 | V | 1.919351193 | K | 116 | 0.827100271 |
| 148 | 0.441690722 | K | 0.57485841 | L | 1.619783499 | A | 117 | 0.715443343 |
| 149 | 0.540984704 | L | 0.592158738 | I | 0.488885463 | F | 118 | 0.264479558 |
| 150 | 0.369688926 | V | 0.757530775 | V | 0.664554325 | F | 119 | 0.245678374 |
| 151 | 0.793500713 | L | 0.693637204 | A | 0.772903461 | I | 120 | 0.613299448 |
| 152 | 0.668891349 | F | 0.874011159 | L | 0.296209229 | F | 121 | 0.19813179 |
| 153 | 0.491542718 | V | 0.782162897 | V | 0.540401376 | Q | 122 | 0.265630361 |
| 154 | 1.014578407 | L | 1.717366435 | A | 1.973346445 | I | 123 | 2.002114694 |
| 155 | 0.610089655 | A | 1.109200641 | A | 0.534896026 | S | 124 | 0.326334532 |
| 156 | 1.065562172 | L | 0.935111904 | L | 0.385452365 | F | 125 | 0.410723459 |
| 157 | 0.639760404 | L | 1.938580604 | G | 2.207610836 | L | 126 | 1.412342001 |
| 158 | 0.6308591 | V | 2.08132097 | G | 2.462729291 | T | 127 | 1.553635184 |
| 159 | 1.054023714 | F | 1.691344685 | L | 0.670057293 | T | 128 | 0.706256277 |
| 160 | 1.016487324 | L | 1.238808384 | L | 0.621939159 | S | 129 | 0.632193272 |
| 161 | 0.483996157 | D | 1.645008645 | F | 1.952819027 | V | 130 | 0.945156905 |
| 162 | 0.505900287 | A | 2.970943333 | G | 3.698233021 | Y | 131 | 1.870937145 |
| 163 | 0.826022394 | M | 1.919188357 | Y | 3.281431389 | M | 132 | 2.710535813 |
| 164 | 1.132436009 | G | 1.858784392 | D | 4.318787416 | G | 133 | 4.890750386 |
| 165 | 0.590459487 | I | 1.245586867 | T | 1.710550075 | S | 134 | 1.01001052 |
| 166 | 0.589762734 | G | 1.92895393 | G | 1.618124573 | A | 135 | 0.954309573 |
| 167 | 0.925456754 | L | 1.38488219 | V | 0.957923638 | V | 136 | 0.8865169 |
| 168 | 1.133384415 | V | 1.725671744 | I | 1.485797893 | Y | 137 | 1.683980176 |
| 169 | 0.599486046 | V | 1.612883206 | S | 3.280533481 | T | 138 | 1.966634045 |
| 170 | 0 | gap | 0.020190489 | gap | 0.007870191 | - | - | 0 |
| 171 | 0 | gap | 0.032898178 | gap | 0.015619413 | - | - | 0 |
| 172 | 0 | gap | 0.022928158 | gap | 0.012999558 | - | - | 0 |
| 173 | 0 | gap | 0.022213265 | gap | 0.009445378 | - | - | 0 |
| 174 | 0 | gap | 0.023730865 | gap | 0.008751869 | - | - | 0 |
| 175 | 0 | gap | 0.034854202 | gap | 0.013291359 | - | - | 0 |
| 176 | 0 | gap | 0.020190489 | gap | 0.007870191 | - | - | 0 |
| 177 | 0 | gap | 0.021285174 | gap | 0.007878278 | - | - | 0 |
| 178 | 0 | gap | 0.029817484 | gap | 0.016871991 | - | - | 0 |
| 179 | 0 | gap | 0.034854202 | gap | 0.013291359 | - | - | 0 |
| 180 | 0 | gap | 0.031634706 | gap | 0.021096624 | - | - | 0 |
| 181 | 0 | gap | 0.034854202 | gap | 0.013291359 | - | - | 0 |
| 182 | 1.516365488 | P | 1.63982561 | G | 1.538688201 | P | 139 | 2.333213685 |
| 183 | 0.806292597 | I | 1.144269993 | A | 0.776927919 | G | 140 | 0.62643123 |
| 184 | 1.29262545 | L | 0.613241159 | L | 0.561155162 | I | 141 | 0.725363443 |
| 185 | 1.001687172 | P | 0.31253791 | P | 0.509413852 | E | 142 | 0.510273321 |
| 186 | 0.32045004 | D | 0.571877055 | M | 1.216086647 | E | 143 | 0.389695015 |
| 187 | 0.186426536 | gap | 0 | gap | 0.090323035 | - | - | 0.016838611 |
| 188 | 0.931489747 | I | 0.660620166 | I | 0.5914793 | L | 144 | 0.550956903 |
| 189 | 0.710630371 | A | 0.450456378 | D | 1.353344867 | M | 145 | 0.961727965 |
| 190 | 0 | gap | 0.031177572 | gap | 0.013422792 | - | - | 0 |
| 191 | 0 | gap | 0.04450221 | gap | 0.02817679 | - | - | 0 |
| 192 | 0 | gap | 0.077300376 | gap | 0.041828285 | - | - | 0 |
| 193 | 0 | gap | 0.030743573 | gap | 0.015511606 | - | - | 0 |
| 194 | 0 | gap | 0.025025631 | gap | 0.015631867 | - | - | 0 |
| 195 | 0.554097241 | E | 0.537836905 | F | 1.20418104 | H | 146 | 0.667233392 |
| 196 | 0.589736662 | D | 0.318779625 | L | 0.521990331 | D | 147 | 0.307836836 |
| 197 | 0.763858158 | F | 0.418313955 | F | 0.370016319 | F | 148 | 0.282639984 |
| 198 | 0.712248451 | G | 0.388903606 | R | 0.860233814 | G | 149 | 0.612700202 |
| 199 | 0.797423684 | V | 0.387105741 | gap | 1.007741439 | I | 150 | 0.803596891 |
| 200 | 0.116904806 | gap | 0.24523286 | gap | 0.345205503 | - | - | 0.040356182 |
| 201 | 0.135782997 | gap | 0.261431276 | gap | 0.472098198 | - | - | 0.064102908 |
| 202 | 0.102371262 | gap | 0.173680235 | gap | 0.147112279 | - | - | 0.01506007 |
| 203 | 0.137338822 | gap | 0.213025799 | gap | 0.329398269 | - | - | 0.04523917 |
| 204 | 0.061236886 | gap | 0.23584835 | gap | 0.32518968 | - | - | 0.019913603 |
| 205 | 0.076692946 | gap | 0.300294265 | gap | 0.389358573 | - | - | 0.029861056 |
| 206 | 0.047062059 | gap | 0.331699593 | gap | 0.318222682 | - | - | 0.014976215 |
| 207 | 0.032993583 | gap | 0.284519724 | gap | 0.180043276 | - | - | 0.005940273 |
| 208 | 0.071070346 | gap | 0.170543233 | gap | 0.248496304 | - | - | 0.017660718 |
| 209 | 0.031209397 | gap | 0.172343358 | gap | 0.186828476 | - | - | 0.005830804 |
| 210 | 0.039326988 | gap | 0.194247419 | gap | 0.279045445 | - | - | 0.010974017 |
| 211 | 0.056345989 | gap | 0.16305724 | gap | 0.1200983 | - | - | 0.006767057 |
| 212 | 0.054099462 | gap | 0.097566833 | gap | 0.157377924 | - | - | 0.008514061 |
| 213 | 0.126685224 | gap | 0.14493109 | gap | 0.148719762 | - | - | 0.018840596 |
| 214 | 0.034730375 | gap | 0.066348356 | gap | 0.0946104 | - | - | 0.003285855 |
| 215 | 0.036446517 | gap | 0.124489365 | gap | 0.082944458 | - | - | 0.003023037 |
| 216 | 0.093326129 | gap | 0.107954762 | gap | 0.291301824 | - | - | 0.027186072 |
| 217 | 0.057566266 | gap | 0.164465046 | gap | 0.145586282 | - | - | 0.008380859 |
| 218 | 0.071250299 | gap | 0.260915638 | gap | 0.331083434 | - | - | 0.023589794 |
| 219 | 0.12592717 | gap | 0.108300424 | gap | 0.247425637 | - | - | 0.03115761 |
| 220 | 0.059412972 | gap | 0.04275002 | gap | 0.109168644 | - | - | 0.006486034 |
| 221 | 0.058515951 | gap | 0.075243745 | gap | 0.112282455 | - | - | 0.006570315 |
| 222 | 0.069119836 | gap | 0.059104454 | gap | 0.1597942 | - | - | 0.011044949 |
| 223 | 0.101433752 | gap | 0.034206066 | gap | 0.061846975 | - | - | 0.006273371 |
| 224 | 0.0629435 | gap | 0.030154897 | gap | 0.086193119 | - | - | 0.005425297 |
| 225 | 0.053154405 | gap | 0.041861824 | gap | 0.123877415 | - | - | 0.00658463 |
| 226 | 0.087125305 | gap | 0.052318597 | gap | 0.181599875 | - | - | 0.015821944 |
| 227 | 0.061505081 | gap | 0.039247578 | gap | 0.093214811 | - | - | 0.005733184 |
| 228 | 0.059990003 | gap | 0.045407569 | gap | 0.110725024 | - | - | 0.006642394 |
| 229 | 0.04738204 | gap | 0.038821324 | gap | 0.06705782 | - | - | 0.003177336 |
| 230 | 0.086375409 | gap | 0.045407569 | gap | 0.04859197 | - | - | 0.004197151 |
| 231 | 0.075744854 | gap | 0.045311073 | gap | 0.134691785 | - | - | 0.01020221 |
| 232 | 0.036524575 | gap | 0.05373456 | gap | 0.089846125 | - | - | 0.003281592 |
| 233 | 0.066678059 | gap | 0.031651523 | gap | 0.05367419 | - | - | 0.003578891 |
| 234 | 0.116884696 | gap | 0.041640122 | gap | 0.147554384 | - | - | 0.017246849 |
| 235 | 0.113436171 | gap | 0.04079693 | gap | 0.127595295 | - | - | 0.014473922 |
| 236 | 0.065716567 | gap | 0.043953814 | gap | 0.099161171 | - | - | 0.006516532 |
| 237 | 0.043119868 | gap | 0.030435543 | gap | 0.040938955 | - | - | 0.001765282 |
| 238 | 0.057124524 | gap | 0.05252975 | gap | 0.090716127 | - | - | 0.005182116 |
| 239 | 0.041213291 | gap | 0.040641419 | gap | 0.068220266 | - | - | 0.002811582 |
| 240 | 0.061696527 | gap | 0.043336622 | gap | 0.093542406 | - | - | 0.005771242 |
| 241 | 0.058341717 | gap | 0.039458282 | gap | 0.09648205 | - | - | 0.005628928 |
| 242 | 0.051498383 | gap | 0.843376695 | L | 0.565868717 | - | - | 0.029141324 |
| 243 | 0.060916592 | gap | 0 | gap | 0.01457677 | - | - | 0.000887967 |
| 244 | 0.034485356 | gap | 0 | gap | 0.012924861 | - | - | 0.000445718 |
| 245 | 0.03174887 | gap | 0 | gap | 0.011155032 | - | - | 0.00035416 |
| 246 | 0.074731024 | gap | 0 | gap | 0.041935744 | - | - | 0.003133901 |
| 247 | 0.013149924 | gap | 0 | gap | 0.005333176 | - | - | 7.01E-05 |
| 248 | 0.012944359 | gap | 0 | gap | 0.004897865 | - | - | 6.34E-05 |
| 249 | 0.01891063 | gap | 0 | gap | 0.00687473 | - | - | 0.000130005 |
| 250 | 0.047667577 | gap | 0 | gap | 0.015608484 | - | - | 0.000744019 |
| 251 | 0.024961752 | gap | 0 | gap | 0.007912115 | - | - | 0.0001975 |
| 252 | 0.034091495 | gap | 0 | gap | 0.010015875 | - | - | 0.000341456 |
| 253 | 0.113722682 | gap | 0 | gap | 0.04007548 | - | - | 0.004557491 |
| 254 | 0.066694568 | gap | 0 | gap | 0.020818528 | - | - | 0.001388483 |
| 255 | 0.060738176 | gap | 0 | gap | 0.024049165 | - | - | 0.001460702 |
| 256 | 0.048633184 | gap | 0 | gap | 0.022254433 | - | - | 0.001082304 |
| 257 | 0.05125092 | gap | 0 | gap | 0.025595823 | - | - | 0.001311809 |
| 258 | 0.043096428 | gap | 0 | gap | 0.015444622 | - | - | 0.000665608 |
| 259 | 0.060617671 | gap | 0 | gap | 0.019360624 | - | - | 0.001173596 |
| 260 | 0.048548999 | gap | 0 | gap | 0.01776154 | - | - | 0.000862305 |
| 261 | 0.058865709 | gap | 0 | gap | 0.019515941 | - | - | 0.00114882 |
| 262 | 0.038425844 | gap | 0 | gap | 0.009663932 | - | - | 0.000371345 |
| 263 | 0.114390611 | gap | 0 | gap | 0.038245527 | - | - | 0.004374929 |
| 264 | 0.067713351 | gap | 0 | gap | 0.018708052 | - | - | 0.001266785 |
| 265 | 0.020024724 | gap | 0 | gap | 0.00742122 | - | - | 0.000148608 |
| 266 | 0.054417572 | gap | 0 | gap | 0.022012538 | - | - | 0.001197869 |
| 267 | 0.056614446 | gap | 0 | gap | 0.021797341 | - | - | 0.001234044 |
| 268 | 0.07057706 | gap | 0 | gap | 0.030795605 | - | - | 0.002173463 |
| 269 | 0.155171395 | gap | 0 | gap | 0.075471477 | - | - | 0.011711014 |
| 270 | 0.059919536 | gap | 0 | gap | 0.018623515 | - | - | 0.001115912 |
| 271 | 0.063667618 | gap | 0 | gap | 0.027269702 | - | - | 0.001736197 |
| 272 | 0.071210254 | gap | 0 | gap | 0.028423482 | - | - | 0.002024043 |
| 273 | 0.033901526 | gap | 0 | gap | 0.011921734 | - | - | 0.000404165 |
| 274 | 0.039898639 | gap | 0 | gap | 0.015998054 | - | - | 0.000638301 |
| 275 | 0.050794828 | gap | 0 | gap | 0.02281518 | - | - | 0.001158893 |
| 276 | 0.110502974 | gap | 0 | gap | 0.034826037 | - | - | 0.003848381 |
| 277 | 0.095145503 | gap | 0 | gap | 0.030707643 | - | - | 0.002921694 |
| 278 | 0.190524751 | gap | 0 | gap | 0.079459588 | - | - | 0.015139018 |
| 279 | 0.5236412 | S | 0.675857726 | S | 0.333734068 | G | 151 | 0.174756908 |
| 280 | 0.473460787 | A | 0.816391587 | S | 0.830388132 | R | 152 | 0.393156219 |
| 281 | 0.550943328 | V | 0.341962977 | L | 0.687899973 | V | 153 | 0.3789939 |
| 282 | 0.295149468 | V | 0.561073561 | L | 0.709864505 | V | 154 | 0.209516131 |
| 283 | 0.940402274 | A | 0.567828857 | Q | 1.643906142 | A | 155 | 1.545933074 |
| 284 | 1.039355597 | G | 1.081392805 | G | 0.590669588 | T | 156 | 0.613915742 |
| 285 | 0.904514471 | L | 0.944832896 | L | 0.608955966 | L | 157 | 0.550809483 |
| 286 | 0.73015626 | L | 0.929656923 | V | 0.680798753 | P | 158 | 0.497089471 |
| 287 | 1.0625196 | L | 1.424502101 | V | 1.216022408 | L | 159 | 1.292047642 |
| 288 | 1.377795823 | A | 1.722005159 | S | 0.38948981 | T | 160 | 0.536637433 |
| 289 | 0.921851499 | V | 1.040726773 | S | 0.55415031 | L | 161 | 0.510844295 |
| 290 | 0.932068928 | Y | 0.925234643 | L | 1.81412174 | F | 162 | 1.690886505 |
| 291 | 1.034547483 | A | 0.584939446 | L | 0.902039301 | V | 163 | 0.933202488 |
| 292 | 1.053700971 | L | 1.286456292 | L | 0.238884041 | I | 164 | 0.251712346 |
| 293 | 0.754439062 | G | 2.450208023 | G | 1.701046764 | G | 165 | 1.283336126 |
| 294 | 0.583080189 | Q | 1.436343693 | A | 2.738507161 | Y | 166 | 1.596769273 |
| 295 | 0.829705454 | A | 1.105981368 | A | 0.595864626 | G | 167 | 0.494392131 |
| 296 | 1.25680231 | L | 1.064093193 | I | 0.228102168 | V | 168 | 0.286679331 |
| 297 | 0.748998093 | G | 2.207346758 | G | 1.308328834 | G | 169 | 0.979935802 |
| 298 | 0.714395047 | P | 1.443581544 | A | 1.347548767 | P | 170 | 0.962682165 |
| 299 | 1.395324955 | P | 1.035020132 | L | 1.748163545 | L | 171 | 2.439256221 |
| 300 | 1.514243703 | F | 0.852085568 | F | 0.632433996 | V | 172 | 0.957659196 |
| 301 | 0.549283796 | L | 0.943606805 | A | 0.882559211 | F | 173 | 0.484775473 |
| 302 | 1.845228643 | G | 1.834013502 | G | 0.345796986 | S | 174 | 0.638074504 |
| 303 | 0.735274754 | P | 0.553904253 | W | 0.989418832 | P | 175 | 0.727494689 |
| 304 | 1.40913772 | L | 1.024073293 | I | 0.501272198 | M | 176 | 0.706361562 |
| 305 | 1.578895952 | S | 0.79055575 | S | 0.562039957 | S | 177 | 0.887402613 |
| 306 | 1.606713441 | D | 1.088983681 | D | 0.312241882 | E | 178 | 0.501683228 |
| 307 | 0.060003541 | gap | 0 | gap | 0.02235167 | N | 179 | 0.001341179 |
| 308 | 0.117104779 | gap | 0 | gap | 0.043313213 | A | 180 | 0.005072184 |
| 309 | 0.960126067 | R | 0.518284148 | R | 0.34140688 | I | 181 | 0.327793645 |
| 310 | 0.610404326 | I | 0.948136996 | F | 0.298850048 | F | 182 | 0.182419362 |
| 311 | 2.064424798 | G | 2.970943333 | G | 0.657589252 | G | 183 | 1.357543558 |
| 312 | 1.922272025 | R | 2.377346625 | R | 0.406432786 | R | 184 | 0.781274375 |
| 313 | 1.392086818 | K | 1.898302615 | R | 0.505717142 | T | 185 | 0.704002167 |
| 314 | 0.819793225 | P | 0.469308297 | K | 0.660428831 | S | 186 | 0.541415081 |
| 315 | 1.198138816 | V | 0.946547354 | S | 2.177174544 | I | 187 | 2.608557329 |
| 316 | 1.241768083 | L | 1.703658547 | L | 0.954954306 | Y | 188 | 1.185831778 |
| 317 | 1.444874355 | L | 0.929121678 | L | 0.459640525 | I | 189 | 0.664122807 |
| 318 | 0.662647766 | I | 0.888729911 | I | 0.472713683 | I | 190 | 0.313242666 |
| 319 | 1.282427043 | G | 0.781163076 | A | 0.7482778 | T | 191 | 0.959611687 |
| 320 | 0.854750364 | L | 0.68972964 | A | 1.329364836 | L | 192 | 1.136275077 |
| 321 | 0 | gap | 0.026077811 | gap | 0.009325928 | - | - | 0 |
| 322 | 0.801366951 | L | 1.022233296 | V | 0.386797318 | F | 193 | 0.309966587 |
| 323 | 0.801076182 | I | 0.993481446 | L | 0.507259645 | L | 194 | 0.40635362 |
| 324 | 0.887913222 | F | 1.37484072 | F | 0.561237307 | F | 195 | 0.498330025 |
| 325 | 0.731868548 | A | 0.977881245 | I | 0.30125967 | V | 196 | 0.220482477 |
| 326 | 0.982920839 | L | 1.294357328 | I | 0.358433655 | I | 197 | 0.352311908 |
| 327 | 0.726903043 | S | 1.673983219 | G | 0.968572953 | L | 198 | 0.704058627 |
| 328 | 0.401628433 | Q | 1.010226668 | A | 1.471092129 | Q | 199 | 0.590832427 |
| 329 | 0.946549563 | L | 1.109524866 | I | 0.182535563 | I | 200 | 0.172778958 |
| 330 | 0.797761761 | L | 1.23735672 | L | 0.494039685 | P | 201 | 0.394125969 |
| 331 | 0.685594901 | F | 0.684312182 | Q | 0.900730397 | T | 202 | 0.617536167 |
| 332 | 1.115968031 | A | 0.982298023 | A | 0.259681781 | A | 203 | 0.289796565 |
| 333 | 0.984489183 | L | 0.777391291 | A | 0.333934993 | L | 204 | 0.328755388 |
| 334 | 1.392385219 | A | 1.663516339 | A | 0.623074445 | V | 205 | 0.867559647 |
| 335 | 0 | gap | 0.205359591 | gap | 0.098354454 | - | - | 0 |
| 336 | 0 | gap | 0.148019866 | gap | 0.051787955 | - | - | 0 |
| 337 | 0 | gap | 0.166915417 | gap | 0.06476997 | - | - | 0 |
| 338 | 0.58427129 | P | 0.475786228 | P | 0.417306582 | N | 206 | 0.243820255 |
| 339 | 0.065775586 | gap | 0 | gap | 0.023217117 | - | - | 0.001527119 |
| 340 | 0.042142381 | gap | 0 | gap | 0.016955052 | - | - | 0.000714526 |
| 341 | 0.044158243 | gap | 0 | gap | 0.014378792 | - | - | 0.000634942 |
| 342 | 0.03015311 | gap | 0 | gap | 0.014300605 | - | - | 0.000431208 |
| 343 | 0.029902997 | gap | 0 | gap | 0.009394336 | - | - | 0.000280919 |
| 344 | 0.900430824 | N | 0.596187303 | N | 0.14763276 | N | 207 | 0.132933088 |
| 345 | 0 | gap | 0.066384954 | gap | 0.028648575 | - | - | 0 |
| 346 | 0 | gap | 0.029745971 | gap | 0.011285743 | - | - | 0 |
| 347 | 0 | gap | 0.020318903 | gap | 0.009436415 | - | - | 0 |
| 348 | 0 | gap | 0.031724692 | gap | 0.015007657 | - | - | 0 |
| 349 | 0 | gap | 0.026233751 | gap | 0.011717529 | - | - | 0 |
| 350 | 0 | gap | 0.020318903 | gap | 0.009436415 | - | - | 0 |
| 351 | 0 | gap | 0.019360117 | gap | 0.007496931 | - | - | 0 |
| 352 | 0 | gap | 0.023062545 | gap | 0.008456286 | - | - | 0 |
| 353 | 0 | gap | 0.031724692 | gap | 0.015007657 | - | - | 0 |
| 354 | 0 | gap | 0.023062545 | gap | 0.008456286 | - | - | 0 |
| 355 | 0 | gap | 0.062739765 | gap | 0.03792484 | - | - | 0 |
| 356 | 0 | gap | 0.04221167 | gap | 0.020225496 | - | - | 0 |
| 357 | 0 | gap | 0.094094702 | gap | 0.038190247 | - | - | 0 |
| 358 | 0 | gap | 0.062600573 | gap | 0.028590519 | - | - | 0 |
| 359 | 0 | gap | 0.042112023 | gap | 0.016539384 | - | - | 0 |
| 360 | 0.872299913 | I | 0.809735056 | L | 0.864209306 | I | 208 | 0.753849702 |
| 361 | 0.498461965 | A | 0.469496331 | E | 0.387426087 | A | 209 | 0.193117169 |
| 362 | 0.708820329 | V | 0.841091863 | M | 0.583589506 | G | 210 | 0.413660105 |
| 363 | 1.733951435 | L | 1.576372146 | L | 0.190061024 | L | 211 | 0.329556585 |
| 364 | 0.762469596 | L | 1.047077472 | I | 0.385645957 | C | 212 | 0.294043317 |
| 365 | 1.089397081 | I | 0.921968301 | V | 0.19694042 | I | 213 | 0.214546318 |
| 366 | 0.931260562 | L | 1.738097012 | G | 1.564082129 | L | 214 | 1.456568002 |
| 367 | 1.982705808 | R | 2.508492584 | R | 0.415276549 | R | 215 | 0.823371226 |
| 368 | 0.815359034 | F | 1.123441417 | I | 0.275287845 | F | 216 | 0.224458431 |
| 369 | 1.320969724 | L | 1.544097412 | L | 0.321498212 | L | 217 | 0.424689404 |
| 370 | 0.832570011 | Q | 0.792192417 | L | 1.198318056 | G | 218 | 0.997683677 |
| 371 | 2.224244334 | G | 2.970943333 | G | 0.832892849 | G | 219 | 1.852557199 |
| 372 | 1.086501371 | L | 1.390992361 | L | 0.152803474 | F | 220 | 0.166021184 |
| 373 | 0.906270779 | G | 1.684429405 | G | 1.265295773 | F | 221 | 1.146700585 |
| 374 | 0.785461261 | A | 1.313664405 | V | 1.775495786 | A | 222 | 1.394583159 |
| 375 | 1.363492961 | G | 2.770873545 | G | 1.440023934 | S | 223 | 1.963462498 |
| 376 | 0.521266032 | F | 0.836824919 | I | 0.688035944 | P | 224 | 0.358649766 |
| 377 | 0.680687688 | F | 0.963594896 | A | 0.559903397 | C | 225 | 0.381119349 |
| 378 | 0.457055466 | L | 1.325738551 | S | 1.304785339 | L | 226 | 0.596359271 |
| 379 | 1.321712459 | A | 0.714632227 | V | 1.210701103 | A | 227 | 1.600198733 |
| 380 | 0.742890405 | V | 0.964372432 | L | 0.504672814 | T | 228 | 0.374916592 |
| 381 | 0.805643036 | G | 1.032464458 | V | 0.744000234 | G | 229 | 0.599398607 |
| 382 | 0.461058699 | A | 2.176317006 | P | 2.978113662 | G | 230 | 1.373085209 |
| 383 | 1.578901403 | A | 0.935448967 | L | 2.103182401 | A | 231 | 3.320717644 |
| 384 | 0.814312916 | L | 2.499111397 | Y | 2.545411978 | S | 232 | 2.072761852 |
| 385 | 0.965869218 | I | 1.508677844 | L | 0.589470099 | V | 233 | 0.569351024 |
| 386 | 0.79113535 | A | 1.67171974 | S | 1.382792594 | A | 234 | 1.093976103 |
| 387 | 1.318656325 | D | 2.965587204 | E | 4.641138592 | D | 235 | 6.120066761 |
| 388 | 0.859835003 | I | 0.948605435 | I | 0.226879995 | V | 236 | 0.195079361 |
| 389 | 0.801079496 | Y | 1.767965466 | A | 2.089800582 | V | 237 | 1.674096396 |
| 390 | 0.809664293 | P | 1.922330982 | P | 0.936573649 | K | 238 | 0.758310241 |
| 391 | 0.462625797 | P | 0.666513257 | P | 0.409695922 | F | 239 | 0.189535903 |
| 392 | 0.090865052 | gap | 0 | gap | 0.039975359 | - | - | 0.003632363 |
| 393 | 0.501236148 | E | 0.533886254 | K | 0.242860265 | W | 240 | 0.121730344 |
| 394 | 0.580004707 | E | 0.908641409 | I | 1.501929625 | N | 241 | 0.871126253 |
| 395 | 0.989227828 | R | 2.53967511 | R | 0.91147917 | L | 242 | 0.901660559 |
| 396 | 0.807467323 | G | 2.630421644 | G | 1.964681974 | P | 243 | 1.586416493 |
| 397 | 0.641895101 | R | 0.699415811 | A | 0.687624813 | V | 244 | 0.441382999 |
| 398 | 0.752774224 | A | 1.495672507 | L | 2.334648801 | G | 245 | 1.75746344 |
| 399 | 0.901264314 | L | 1.209691269 | V | 2.37815644 | L | 246 | 2.143347533 |
| 400 | 1.67239411 | G | 1.173970456 | S | 1.54369753 | A | 247 | 2.581670657 |
| 401 | 0.790359479 | L | 0.588958769 | L | 0.46045375 | A | 248 | 0.363923985 |
| 402 | 0.815890875 | L | 0.957173353 | Y | 2.255994625 | W | 249 | 1.840645429 |
| 403 | 0.648439782 | S | 1.568274555 | Q | 2.065766553 | S | 250 | 1.339525214 |
| 404 | 0.709070416 | A | 1.729994359 | L | 1.729155995 | L | 251 | 1.226093361 |
| 405 | 0.842361139 | G | 0.950979662 | F | 0.995048521 | G | 252 | 0.838190206 |
| 406 | 0.451947764 | F | 1.712596806 | I | 1.535377502 | A | 253 | 0.693910429 |
| 407 | 0.69366653 | A | 1.521815164 | T | 1.314653182 | V | 254 | 0.911930911 |
| 408 | 0.717682314 | I | 0.902360018 | I | 0.349410068 | C | 255 | 0.250765426 |
| 409 | 1.723418421 | G | 2.809052484 | G | 0.851078402 | G | 256 | 1.466764195 |
| 410 | 0.74166291 | P | 1.589538021 | I | 2.673874957 | P | 257 | 1.983113883 |
| 411 | 1.251245987 | L | 1.671139609 | L | 1.539125234 | S | 258 | 1.925824272 |
| 412 | 1.001697081 | L | 1.08647241 | L | 0.071021742 | F | 259 | 0.071142272 |
| 413 | 2.058465627 | G | 1.795371126 | A | 0.699892322 | G | 260 | 1.440704288 |
| 414 | 1.671462772 | P | 0.855371938 | Y | 3.370416737 | P | 261 | 5.633526104 |
| 415 | 0.871510697 | P | 0.870540652 | I | 0.853695945 | F | 262 | 0.744005148 |
| 416 | 1.179798572 | L | 1.015243378 | I | 0.448834571 | F | 263 | 0.529534385 |
| 417 | 2.558735402 | G | 1.488017401 | N | 2.306141242 | G | 264 | 5.90080524 |
| 418 | 1.315112434 | G | 0.72958097 | Y | 2.468516455 | S | 265 | 3.246376683 |
| 419 | 0.756399373 | L | 0.668770731 | G | 0.834968188 | I | 266 | 0.631569414 |
| 420 | 1.226058003 | L | 0.841871195 | T | 1.045885016 | L | 267 | 1.282315694 |
| 421 | 0.640832427 | G | 0.393965192 | A | 0.691174474 | T | 268 | 0.442927016 |
| 422 | 0.063078618 | gap | 0 | gap | 0.029020622 | - | - | 0.001830581 |
| 423 | 0.023326554 | gap | 0 | gap | 0.008530943 | - | - | 0.000198997 |
| 424 | 0.019375955 | gap | 0 | gap | 0.005760796 | - | - | 0.000111621 |
| 425 | 0.018933303 | gap | 0 | gap | 0.009276308 | - | - | 0.000175631 |
| 426 | 0.416137516 | E | 0.32939882 | K | 0.571953661 | V | 269 | 0.238011376 |
| 427 | 0.294573208 | F | 0.304721568 | G | 0.560176218 | K | 270 | 0.165012906 |
| 428 | 0.473806407 | F | 0.216376352 | gap | 0.847526066 | A | 271 | 0.40156328 |
| 429 | 0 | gap | 0.145806646 | gap | 0.073885601 | - | - | 0 |
| 430 | 0 | gap | 0.153387012 | gap | 0.068189932 | - | - | 0 |
| 431 | 0 | gap | 0.145668126 | gap | 0.075985677 | - | - | 0 |
| 432 | 0 | gap | 0.024343454 | gap | 0.00921238 | - | - | 0 |
| 433 | 0 | gap | 0.045777668 | gap | 0.018209232 | - | - | 0 |
| 434 | 0 | gap | 0.078238601 | gap | 0.043938313 | - | - | 0 |
| 435 | 0 | gap | 0.046126803 | gap | 0.025768797 | - | - | 0 |
| 436 | 0 | gap | 0.047405689 | gap | 0.016162174 | - | - | 0 |
| 437 | 0 | gap | 0.142121552 | gap | 0.080037166 | - | - | 0 |
| 438 | 0 | gap | 0.179146895 | gap | 0.104806737 | - | - | 0 |
| 439 | 0 | gap | 0.132528386 | gap | 0.072801724 | - | - | 0 |
| 440 | 0 | gap | 0.375258937 | gap | 0.182412613 | - | - | 0 |
| 441 | 0 | gap | 0.337779248 | gap | 0.173036239 | - | - | 0 |
| 442 | 0 | gap | 0.469638361 | G | 0.208720465 | - | - | 0 |
| 443 | 1.045030352 | G | 0.686369946 | G | 0.480601217 | S | 272 | 0.502242859 |
| 444 | 1.055702396 | W | 2.409860416 | W | 0.956471073 | W | 273 | 1.009748804 |
| 445 | 1.615274487 | R | 1.996705723 | R | 0.550735726 | R | 274 | 0.889589367 |
| 446 | 0.584808695 | A | 0.867252061 | I | 0.715293688 | W | 275 | 0.418309969 |
| 447 | 0.870282992 | P | 1.115003941 | M | 1.05630575 | T | 276 | 0.919284928 |
| 448 | 1.917916358 | F | 1.342070049 | L | 1.307745658 | F | 277 | 2.508146789 |
| 449 | 0.853157577 | L | 1.730598888 | G | 1.679984256 | W | 278 | 1.433291297 |
| 450 | 0.91416407 | F | 0.91255751 | L | 0.48392972 | F | 279 | 0.442391163 |
| 451 | 0.842258988 | L | 0.559761519 | A | 1.17786671 | M | 280 | 0.992068823 |
| 452 | 1.156449946 | A | 0.571603078 | A | 0.369593709 | C | 281 | 0.427416624 |
| 453 | 0.848085439 | A | 1.618709747 | V | 0.627548975 | I | 282 | 0.532215148 |
| 454 | 0.796180575 | L | 1.702796121 | P | 3.20502115 | I | 283 | 2.551775583 |
| 455 | 0.741152527 | A | 1.778063672 | A | 1.182370255 | S | 284 | 0.876316702 |
| 456 | 1.016687796 | L | 1.350042492 | L | 0.741240477 | G | 285 | 0.753610147 |
| 457 | 0.931556803 | L | 1.385253622 | L | 0.296590312 | F | 286 | 0.276290723 |
| 458 | 0.763963534 | A | 1.085710069 | L | 2.47253762 | S | 287 | 1.888928577 |
| 459 | 0.883554078 | F | 0.879990901 | F | 0.106580541 | F | 288 | 0.094169671 |
| 460 | 0.957865664 | I | 1.105596892 | L | 0.270100493 | V | 289 | 0.258719988 |
| 461 | 0.702073098 | L | 1.3381027 | G | 0.973906268 | M | 290 | 0.683753391 |
| 462 | 0.666851241 | I | 1.001489565 | L | 0.521671696 | L | 291 | 0.347877418 |
| 463 | 0.597791775 | L | 1.014274623 | L | 0.736138876 | C | 292 | 0.440057765 |
| 464 | 0.746459771 | F | 1.148936982 | F | 0.48164455 | F | 293 | 0.35952828 |
| 465 | 0.694401038 | L | 1.061760719 | L | 0.655598983 | T | 294 | 0.455248615 |
| 466 | 1.168579968 | L | 0.25865774 | gap | 0.222276477 | L | 295 | 0.259747838 |
| 467 | 0 | gap | 0.025264049 | gap | 0.010837819 | - | - | 0 |
| 468 | 1.39191 | P | 2.188762416 | P | 0.550019165 | P | 296 | 0.765577176 |
| 469 | 1.154287747 | E | 2.289468098 | E | 0.655171551 | E | 297 | 0.756256494 |
| 470 | 0.832045633 | T | 2.223755624 | S | 1.363272353 | T | 298 | 1.134304808 |
| 471 | 0.40145701 | gap | 2.70366069 | P | 3.244545703 | F | 299 | 1.302545616 |
| 472 | 0.381278056 | gap | 1.845918398 | R | 1.791322592 | G | 300 | 0.682991996 |
| 473 | 0.344673572 | gap | 1.523362607 | Y | 3.069389047 | K | 301 | 1.057937288 |
| 474 | 0.352647438 | gap | 0.175818492 | gap | 0.71990342 | T | 302 | 0.253872096 |
| 475 | 0.361786787 | gap | 1.808713947 | L | 2.230097147 | L | 303 | 0.806819682 |
| 476 | 0.617369266 | R | 1.055804585 | V | 1.824760826 | L | 304 | 1.126551252 |
| 477 | 0.253194183 | gap | 0.623661695 | E | 0.56681116 | Y | 305 | 0.143513289 |
| 478 | 0.414354262 | P | 1.263131008 | K | 1.227677226 | R | 306 | 0.508693291 |
| 479 | 0.365771305 | E | 1.746207089 | G | 2.177424561 | K | 307 | 0.796439424 |
| 480 | 0.430427951 | S | 0.956472285 | R | 1.224636647 | A | 308 | 0.527117843 |
| 481 | 0.49656773 | K | 0.402251997 | E | 0.657523594 | K | 309 | 0.326504999 |
| 482 | 0.48897372 | R | 0.761102281 | E | 1.175183039 | R | 310 | 0.574633622 |
| 483 | 0.410272164 | G | 1.152772222 | E | 0.997221559 | L | 311 | 0.409132246 |
| 484 | 0.437527001 | R | 2.415010527 | A | 2.634820023 | R | 312 | 1.152804902 |
| 485 | 0.469072615 | L | 0.598449502 | K | 0.698816666 | A | 313 | 0.327795761 |
| 486 | 0.471972833 | L | 0.823530366 | R | 0.891123416 | I | 314 | 0.420586043 |
| 487 | 0.4976698 | T | 1.140238037 | V | 2.158773395 | T | 315 | 1.074356324 |
| 488 | 0.318452341 | G | 1.977734107 | L | 2.388966098 | G | 316 | 0.760771848 |
| 489 | 0 | gap | 0.560242508 | R | 0.333460237 | - | - | 0 |
| 490 | 0 | gap | 0.844751096 | R | 0.508263655 | - | - | 0 |
| 491 | 0 | gap | 1.064617465 | L | 0.567508614 | - | - | 0 |
| 492 | 0 | gap | 0.842993361 | R | 0.516784488 | - | - | 0 |
| 493 | 0 | gap | 0.930977002 | G | 0.462382116 | - | - | 0 |
| 494 | 0 | gap | 0.347941408 | T | 0.153762171 | - | - | 0 |
| 495 | 0 | gap | 0.573715779 | D | 0.357203707 | - | - | 0 |
| 496 | 0 | gap | 0.454072201 | D | 0.225219906 | - | - | 0 |
| 497 | 0 | gap | 0.403501368 | gap | 0.228435745 | - | - | 0 |
| 498 | 0 | gap | 0.246231223 | gap | 0.153902282 | - | - | 0 |
| 499 | 0 | gap | 0.15434429 | gap | 0.090323473 | - | - | 0 |
| 500 | 0 | gap | 0.109692689 | gap | 0.053765364 | - | - | 0 |
| 501 | 0 | gap | 0.02737131 | gap | 0.011444376 | - | - | 0 |
| 502 | 0 | gap | 0.03124703 | gap | 0.011630784 | - | - | 0 |
| 503 | 0.441233453 | L | 0.883376794 | V | 0.934365763 | N | 317 | 0.412273432 |
| 504 | 0.157583041 | gap | 0.45892624 | E | 0.144109172 | D | 318 | 0.022709162 |
| 505 | 0.126712182 | gap | 0.356367169 | Q | 0.387246862 | R | 319 | 0.049068895 |
| 506 | 0.186049068 | gap | 1.130833755 | E | 1.559855343 | I | 320 | 0.290209633 |
| 507 | 0.065294945 | gap | 0.862304389 | L | 0.788275777 | T | 321 | 0.051470424 |
| 508 | 0.22845539 | gap | 0.450363991 | D | 1.178418574 | S | 322 | 0.269216075 |
| 509 | 0.184387321 | gap | 0.904026824 | E | 0.372941507 | E | 323 | 0.068765685 |
| 510 | 0.077305255 | gap | 1.179899577 | I | 1.365862713 | G | 324 | 0.105588365 |
| 511 | 0.134739198 | gap | 0.557358691 | K | 0.554441133 | E | 325 | 0.074704953 |
| 512 | 0.116705038 | gap | 0.691619385 | A | 0.544582406 | I | 326 | 0.06355551 |
| 513 | 0.137426215 | gap | 0.679470759 | A | 0.262824229 | E | 327 | 0.036118939 |
| 514 | 0.213064788 | gap | 0.454615709 | I | 1.17683904 | N | 328 | 0.250742961 |
| 515 | 0.080704884 | gap | 0.885356158 | E | 0.821437207 | S | 329 | 0.066293994 |
| 516 | 0.166121562 | gap | 0.4869337 | E | 0.34612112 | K | 330 | 0.057498181 |
| 517 | 0.142836008 | gap | 0.85162345 | E | 1.32534331 | M | 331 | 0.189306748 |
| 518 | 0.14340895 | gap | 0.372948816 | K | 0.359148182 | T | 332 | 0.051505064 |
| 519 | 0.069187681 | gap | 0.315374882 | A | 0.216052728 | S | 333 | 0.014948187 |
| 520 | 0.091700744 | gap | 0.407265479 | V | 0.378267179 | H | 334 | 0.034687382 |
| 521 | 0.112422247 | gap | 0.458624966 | G | 0.442453408 | E | 335 | 0.049741606 |
| 522 | 0.164956531 | gap | 0.281799258 | K | 0.79278265 | L | 336 | 0.130774676 |
| 523 | 0.040392463 | gap | 0.449397646 | A | 1.27353036 | - | - | 0.051441028 |
| 524 | 0.033195355 | gap | 0.46316466 | S | 0.446141303 | - | - | 0.014809819 |
| 525 | 0.029308677 | gap | 0.368601327 | P | 0.566280606 | - | - | 0.016596935 |
| 526 | 0.042585343 | gap | 0.135772623 | gap | 0.176115462 | - | - | 0.007499937 |
| 527 | 0.029308677 | gap | 0.164875923 | gap | 0.107648198 | - | - | 0.003155026 |
| 528 | 0.031267719 | gap | 0.077878717 | gap | 0.169816807 | - | - | 0.005309784 |
| 529 | 0.027261783 | gap | 0.031264365 | gap | 0.042179852 | - | - | 0.001149898 |
| 530 | 0.040392463 | gap | 0.031261386 | gap | 0.071684373 | - | - | 0.002895508 |
| 531 | 0.028898294 | gap | 0.042907499 | gap | 0.05929382 | - | - | 0.00171349 |
| 532 | 0.026151886 | gap | 0.036742541 | gap | 0.045648597 | - | - | 0.001193797 |
| 533 | 0.026151886 | gap | 0.038134341 | gap | 0.039594163 | - | - | 0.001035462 |
| 534 | 0.033195355 | gap | 0.043737673 | gap | 0.069538522 | - | - | 0.002308356 |
| 535 | 0.028180336 | gap | 0.031261386 | gap | 0.041485412 | - | - | 0.001169073 |
| 536 | 0.031124623 | gap | 0.075552946 | gap | 0.12627821 | - | - | 0.003930362 |
| 537 | 0.031124623 | gap | 0.030559748 | gap | 0.065024036 | - | - | 0.002023849 |
| 538 | 0.023907089 | gap | 0.040509931 | gap | 0.073906966 | - | - | 0.0017669 |
| 539 | 0.095343308 | gap | 0.075227326 | gap | 0.124934135 | - | - | 0.011911634 |
| 540 | 0.040566829 | gap | 0.047432167 | gap | 0.060711001 | - | - | 0.002462853 |
| 541 | 0.065195535 | gap | 0.031055145 | gap | 0.107042358 | - | - | 0.006978684 |
| 542 | 0.04617194 | gap | 0.036261134 | gap | 0.072483559 | - | - | 0.003346707 |
| 543 | 0.0396276 | gap | 0.030353827 | gap | 0.024714856 | - | - | 0.00097939 |
| 544 | 0.16122935 | gap | 0.028302198 | gap | 0.19428604 | I | 337 | 0.031324612 |
| 545 | 0.076203639 | gap | 0.086571255 | gap | 0.172970415 | I | 338 | 0.013180975 |
| 546 | 0.109948677 | gap | 0.148860847 | gap | 0.381877958 | D | 339 | 0.041986976 |
| 547 | 0.264949893 | gap | 0.22488304 | gap | 0.397560695 | T | 340 | 0.105333663 |
| 548 | 0.151233137 | gap | 0.186644014 | gap | 0.367972727 | L | 341 | 0.05564967 |
| 549 | 0.181814736 | gap | 0.354140979 | gap | 0.693895927 | W | 342 | 0.126160505 |
| 550 | 0.289215272 | gap | 0.456968264 | L | 0.57901422 | R | 343 | 0.167459755 |
| 551 | 0.204571764 | gap | 0.63617461 | L | 0.91100258 | P | 344 | 0.186365405 |
| 552 | 0.239683486 | gap | 0.368336615 | gap | 0.700261702 | L | 345 | 0.167841166 |
| 553 | 0.174458778 | gap | 0.263605888 | G | 0.420421945 | E | 346 | 0.073346299 |
| 554 | 0.326277699 | gap | 0.758368564 | P | 1.205940328 | I | 347 | 0.393471435 |
| 555 | 0.443542659 | gap | 0.345450851 | R | 1.390039737 | T | 348 | 0.616541921 |
| 556 | 0.684058677 | L | 0.577428936 | L | 0.651932241 | V | 349 | 0.445959906 |
| 557 | 0.632006342 | K | 1.239399657 | R | 1.255776231 | M | 350 | 0.793658541 |
| 558 | 0.431162005 | R | 0.619073022 | R | 1.589439499 | E | 351 | 0.685305921 |
| 559 | 0.857531682 | P | 0.741864627 | R | 1.374602076 | P | 352 | 1.178764831 |
| 560 | 0.359434622 | V | 1.374973241 | L | 1.583161756 | V | 353 | 0.569043147 |
| 561 | 0.8916426 | I | 1.064475337 | I | 0.301732487 | V | 354 | 0.26903754 |
| 562 | 0.625975518 | L | 0.871661268 | I | 0.450945903 | L | 355 | 0.282281096 |
| 563 | 0.63577826 | L | 1.782495006 | G | 1.677343031 | L | 356 | 1.066418233 |
| 564 | 0.626680304 | L | 0.909024971 | I | 0.600143971 | I | 357 | 0.376098407 |
| 565 | 0.410507208 | A | 0.819985722 | G | 0.497648622 | N | 358 | 0.204288346 |
| 566 | 1.258501255 | L | 1.817025915 | L | 0.723464557 | I | 359 | 0.910481053 |
| 567 | 0.697297581 | A | 0.965416159 | Q | 1.54820003 | Y | 360 | 1.079556136 |
| 568 | 0.675155524 | I | 0.793246665 | A | 0.830939277 | I | 361 | 0.561013243 |
| 569 | 0.563696071 | A | 1.101638143 | F | 0.455022848 | A | 362 | 0.256494592 |
| 570 | 0.668428403 | L | 2.094316375 | Q | 2.805762422 | M | 363 | 1.875451294 |
| 571 | 0.655172518 | V | 2.818368258 | Q | 4.414354855 | V | 364 | 2.892163984 |
| 572 | 0.624431378 | Y | 1.538819316 | L | 1.270316564 | Y | 365 | 0.793225523 |
| 573 | 0.742719632 | A | 1.505927489 | T | 1.815866149 | S | 366 | 1.348679437 |
| 574 | 0.71119788 | G | 2.813343031 | G | 2.637973484 | I | 367 | 1.876121149 |
| 575 | 0.577500129 | L | 1.187138768 | I | 1.221717887 | L | 368 | 0.705542238 |
| 576 | 0.625802011 | Y | 2.5750323 | N | 4.612910599 | Y | 369 | 2.886768729 |
| 577 | 0.707495434 | A | 0.832735525 | A | 0.556641372 | L | 370 | 0.393821229 |
| 578 | 0.659401768 | F | 1.646305317 | I | 1.548428793 | F | 371 | 1.021036684 |
| 579 | 0.64002199 | F | 1.169941161 | M | 1.053239888 | F | 372 | 0.674096689 |
| 580 | 0.737654695 | T | 1.774308061 | Y | 3.100669959 | E | 373 | 2.287223752 |
| 581 | 0.60194736 | A | 2.496010209 | Y | 2.671136169 | V | 374 | 1.607883364 |
| 582 | 0.652833211 | F | 1.435139876 | A | 2.089212485 | F | 375 | 1.363907295 |
| 583 | 0.58375023 | P | 0.902594125 | P | 0.713923759 | P | 376 | 0.416753158 |
| 584 | 0.80702071 | I | 0.79577027 | T | 1.889067206 | I | 377 | 1.524516357 |
| 585 | 0.716942606 | V | 1.615934528 | I | 1.692247576 | Y | 378 | 1.213244387 |
| 586 | 0.575368668 | M | 1.465938164 | F | 0.96090598 | F | 379 | 0.552875194 |
| 587 | 0.332432701 | M | 0.02390844 | gap | 0.439604692 | V | 380 | 0.146138975 |
| 588 | 0.473884387 | G | 0.778183662 | Q | 0.864340232 | G | 381 | 0.409597341 |
| 589 | 0.416938026 | T | 0.543761781 | S | 0.777815886 | V | 382 | 0.32430102 |
| 590 | 0.392498505 | Y | 1.032719806 | A | 0.879640135 | K | 383 | 0.345257438 |
| 591 | 0.580854132 | G | 2.466121601 | G | 1.697169026 | H | 384 | 0.985807642 |
| 592 | 0.757813697 | F | 1.040327993 | F | 0.643349616 | F | 385 | 0.487539151 |
| 593 | 0 | gap | 0.086532611 | gap | 0.049664026 | - | - | 0 |
| 594 | 0.81017967 | S | 0.495555802 | gap | 0.219134938 | T | 386 | 0.177538671 |
| 595 | 0.432591704 | E | 0.374337903 | gap | 0.549083182 | L | 387 | 0.237528829 |
| 596 | 0.330765894 | W | 0.549407721 | D | 1.166982503 | V | 388 | 0.385998011 |
| 597 | 0.342903818 | E | 0.513945063 | A | 0.889676765 | E | 389 | 0.30507356 |
| 598 | 0.684877456 | I | 1.264668607 | A | 1.489248523 | L | 390 | 1.01995274 |
| 599 | 1.219953736 | G | 0.727141248 | L | 2.284575199 | G | 391 | 2.787076049 |
| 600 | 0.901627036 | L | 0.814163052 | L | 0.280069049 | T | 392 | 0.252517826 |
| 601 | 0.679502276 | A | 0.509808096 | A | 0.219507722 | T | 393 | 0.149155997 |
| 602 | 0.760615045 | L | 1.237555994 | S | 2.8734264 | Y | 394 | 2.18557135 |
| 603 | 0.881157721 | L | 1.283609203 | V | 0.871606551 | M | 395 | 0.768022842 |
| 604 | 0.717595424 | L | 1.064438137 | I | 0.906687817 | S | 396 | 0.650635028 |
| 605 | 0.553906638 | F | 0.85933295 | T | 0.709258134 | I | 397 | 0.392862788 |
| 606 | 1.069054286 | G | 1.491140939 | G | 0.826496587 | V | 398 | 0.883569719 |
| 607 | 1.051808868 | I | 1.017743269 | A | 0.279201349 | I | 399 | 0.293666455 |
| 608 | 0.80537356 | A | 1.246664562 | V | 1.758729113 | G | 400 | 1.416433926 |
| 609 | 0.478104148 | A | 1.539206901 | N | 2.061764474 | I | 401 | 0.985738147 |
| 610 | 0.842712564 | L | 1.253302914 | V | 0.29611287 | V | 402 | 0.249538035 |
| 611 | 0.878330384 | L | 0.94559338 | L | 0.299291524 | I | 403 | 0.262876839 |
| 612 | 0.801897769 | G | 0.902373408 | F | 0.547861634 | A | 404 | 0.439329021 |
| 613 | 0.498116882 | A | 2.339868433 | T | 2.266013797 | A | 405 | 1.128739726 |
| 614 | 0.328311896 | A | 1.116975717 | L | 1.156173813 | F | 406 | 0.379585617 |
| 615 | 0.684420636 | L | 1.261091864 | V | 1.008599637 | I | 407 | 0.690306405 |
| 616 | 0.459299862 | A | 1.872846709 | A | 2.14702395 | Y | 408 | 0.986127804 |
| 617 | 0.756383372 | G | 0.971113369 | I | 1.055341998 | I | 409 | 0.798243139 |
| 618 | 0.530642975 | R | 0.683795292 | Y | 1.471604394 | P | 410 | 0.780896534 |
| 619 | 0.7127445 | L | 0.850249319 | L | 0.61665766 | V | 411 | 0.439519355 |
| 620 | 0.554545454 | L | 1.502564343 | V | 1.492379855 | I | 412 | 0.827592464 |
| 621 | 0.257679397 | D | 1.908957268 | D | 2.060790357 | R | 413 | 0.531023216 |
| 622 | 0.795585735 | R | 1.68587689 | R | 0.739878134 | Q | 414 | 0.588636489 |
| 623 | 0.515113526 | R | 0.954343381 | F | 1.270261805 | K | 415 | 0.654329038 |
| 624 | 0.473611164 | G | 2.29452816 | G | 1.999237843 | F | 416 | 0.946861363 |
| 625 | 0.134171018 | gap | 0 | gap | 0.053108204 | T | 417 | 0.007125582 |
| 626 | 0.139521953 | gap | 0 | gap | 0.058372528 | K | 418 | 0.008144249 |
| 627 | 0.09144111 | gap | 0 | gap | 0.053722786 | P | 419 | 0.004912471 |
| 628 | 0.192304726 | gap | 0 | gap | 0.079949059 | I | 420 | 0.015374582 |
| 629 | 0.090550577 | gap | 0 | gap | 0.034176238 | - | - | 0.003094678 |
| 630 | 0.126828433 | gap | 0 | gap | 0.050443689 | L | 421 | 0.006397694 |
| 631 | 0.152640341 | gap | 0 | gap | 0.072431319 | R | 422 | 0.011055941 |
| 632 | 0.143207114 | gap | 0 | gap | 0.067834105 | Q | 423 | 0.009714326 |
| 633 | 0.155350081 | gap | 0 | gap | 0.068471768 | E | 424 | 0.010637095 |
| 634 | 0.069834163 | gap | 0 | gap | 0.026842344 | - | - | 0.001874513 |
| 635 | 0.092296263 | gap | 0 | gap | 0.032275054 | Q | 425 | 0.002978867 |
| 636 | 0.158585446 | gap | 0 | gap | 0.061636293 | V | 426 | 0.009774619 |
| 637 | 0.091300832 | gap | 0 | gap | 0.03917061 | F | 427 | 0.003576309 |
| 638 | 0.291911289 | gap | 0 | gap | 0.127919882 | P | 428 | 0.037341258 |
| 639 | 0.379382294 | gap | 0 | gap | 0.169117854 | E | 429 | 0.06416032 |
| 640 | 0.026156145 | gap | 0 | gap | 0.008185666 | - | - | 0.000214105 |
| 641 | 0.027261783 | gap | 0 | gap | 0.009766405 | - | - | 0.00026625 |
| 642 | 0.033064962 | gap | 0 | gap | 0.01008208 | - | - | 0.000333364 |
| 643 | 0.033064962 | gap | 0 | gap | 0.01008208 | - | - | 0.000333364 |
| 644 | 0.028574614 | gap | 0 | gap | 0.010275634 | - | - | 0.000293622 |
| 645 | 0.028574614 | gap | 0 | gap | 0.010275634 | - | - | 0.000293622 |
| 646 | 0.028426899 | gap | 0 | gap | 0.013525219 | - | - | 0.00038448 |
| 647 | 0.033195355 | gap | 0 | gap | 0.012525061 | - | - | 0.000415774 |
| 648 | 0.026151886 | gap | 0 | gap | 0.010245994 | - | - | 0.000267952 |
| 649 | 0.033195355 | gap | 0 | gap | 0.012525061 | - | - | 0.000415774 |
| 650 | 0.033064962 | gap | 0 | gap | 0.01008208 | - | - | 0.000333364 |
| 651 | 0.026151886 | gap | 0 | gap | 0.010245994 | - | - | 0.000267952 |
| 652 | 0.033064962 | gap | 0 | gap | 0.01008208 | - | - | 0.000333364 |
| 653 | 0.028898294 | gap | 0 | gap | 0.009012078 | - | - | 0.000260434 |
| 654 | 0.023907089 | gap | 0 | gap | 0.009819578 | - | - | 0.000234758 |
| 655 | 0.031267719 | gap | 0 | gap | 0.01166791 | - | - | 0.000364829 |
| 656 | 0.029308677 | gap | 0 | gap | 0.010089012 | - | - | 0.000295696 |
| 657 | 0.029308677 | gap | 0 | gap | 0.010089012 | - | - | 0.000295696 |
| 658 | 0.028574614 | gap | 0 | gap | 0.010275634 | - | - | 0.000293622 |
| 659 | 0.026151886 | gap | 0 | gap | 0.010245994 | - | - | 0.000267952 |
| 660 | 0.033195355 | gap | 0 | gap | 0.012525061 | - | - | 0.000415774 |
| 661 | 0.040392463 | gap | 0 | gap | 0.016173615 | - | - | 0.000653292 |
| 662 | 0.027261783 | gap | 0 | gap | 0.009766405 | - | - | 0.00026625 |
| 663 | 0.032057181 | gap | 0 | gap | 0.014377927 | - | - | 0.000460916 |
| 664 | 0.024913332 | gap | 0 | gap | 0.008211808 | - | - | 0.000204583 |
| 665 | 0.031124623 | gap | 0 | gap | 0.01319724 | - | - | 0.000410759 |
| 666 | 0.026151886 | gap | 0 | gap | 0.010245994 | - | - | 0.000267952 |
| 667 | 0.029308677 | gap | 0 | gap | 0.010089012 | - | - | 0.000295696 |
| 668 | 0.031124623 | gap | 0 | gap | 0.01319724 | - | - | 0.000410759 |
| 669 | 0.027261783 | gap | 0 | gap | 0.009766405 | - | - | 0.00026625 |
| 670 | 0.347989351 | gap | 0 | gap | 0.114977336 | V | 430 | 0.040010888 |
| 671 | 0.368590778 | gap | 2.566195927 | R | 2.064013055 | F | 431 | 0.760776177 |
| 672 | 0.549090807 | L | 2.003061245 | R | 1.68351949 | I | 432 | 0.924405075 |
| 673 | 0.284398615 | R | 0.510374719 | P | 0.610329329 | P | 433 | 0.173576816 |
| 674 | 0.697261004 | L | 1.208721036 | L | 0.499012233 | I | 434 | 0.34794177 |
| 675 | 0.561344269 | I | 1.43048931 | L | 0.91646825 | A | 435 | 0.5144542 |
| 676 | 0.592144488 | L | 1.226156694 | L | 0.490999911 | I | 436 | 0.290742891 |
| 677 | 0.559608796 | L | 0.65365455 | I | 0.555118817 | V | 437 | 0.310649373 |
| 678 | 0.73558945 | G | 1.991021141 | G | 1.163219307 | G | 438 | 0.85565185 |
| 679 | 0.603325145 | L | 0.687066502 | G | 0.661680607 | G | 439 | 0.399208548 |
| 680 | 0.72735212 | I | 0.794237179 | A | 0.575514646 | I | 440 | 0.418601798 |
| 681 | 0.616175059 | L | 0.875386044 | G | 1.494307887 | L | 441 | 0.92075525 |
| 682 | 0.52299454 | L | 1.23449001 | M | 1.137415002 | L | 442 | 0.594861835 |
| 683 | 0.737146708 | V | 0.797901167 | A | 0.457454018 | T | 443 | 0.337210724 |
| 684 | 0.773773461 | I | 0.973859449 | I | 0.246731591 | S | 444 | 0.190914357 |
| 685 | 0.848330673 | G | 0.797745662 | C | 0.645570417 | G | 445 | 0.547657186 |
| 686 | 0.804747326 | L | 0.701939537 | L | 0.659178563 | L | 446 | 0.530472186 |
| 687 | 0.82863355 | L | 0.905642191 | L | 0.341033065 | F | 447 | 0.28259144 |
| 688 | 0.755487835 | L | 1.023038795 | I | 0.829297361 | I | 448 | 0.626524067 |
| 689 | 0.624246133 | L | 0.830732441 | L | 0.316307674 | F | 449 | 0.197453843 |
| 690 | 0.727024995 | G | 1.406731952 | A | 1.022806589 | G | 450 | 0.743605956 |
| 691 | 0.518777395 | W | 0.820109102 | I | 1.049476235 | W | 451 | 0.544444548 |
| 692 | 0.680779636 | A | 0.729434285 | V | 0.530448438 | S | 452 | 0.361118494 |
| 693 | 0.385366845 | A | 0.841363542 | G | 1.223625135 | A | 453 | 0.471544557 |
| 694 | 0 | gap | 0.274739964 | V | 0.154096889 | - | - | 0 |
| 695 | 0 | gap | 0.415989808 | F | 0.164665066 | - | - | 0 |
| 696 | 0 | gap | 0.333093872 | A | 0.121588391 | - | - | 0 |
| 697 | 0 | gap | 0.404691666 | L | 0.170584003 | - | - | 0 |
| 698 | 0 | gap | 0.302228728 | gap | 0.167920628 | - | - | 0 |
| 699 | 0 | gap | 0.171769568 | gap | 0.068765886 | - | - | 0 |
| 700 | 0 | gap | 0.15331779 | gap | 0.080247536 | - | - | 0 |
| 701 | 0 | gap | 0.283000709 | gap | 0.165301873 | - | - | 0 |
| 702 | 0 | gap | 0.274238356 | gap | 0.124106077 | - | - | 0 |
| 703 | 0 | gap | 0.220764392 | gap | 0.117754507 | - | - | 0 |
| 704 | 0 | gap | 0.124000028 | gap | 0.065217183 | - | - | 0 |
| 705 | 0 | gap | 0.053536051 | gap | 0.023450175 | - | - | 0 |
| 706 | 0 | gap | 0.051391893 | gap | 0.016018631 | - | - | 0 |
| 707 | 0 | gap | 0.04536783 | gap | 0.017940388 | - | - | 0 |
| 708 | 0 | gap | 0.058414104 | gap | 0.025222135 | - | - | 0 |
| 709 | 0 | gap | 0.04322062 | gap | 0.018352297 | - | - | 0 |
| 710 | 0 | gap | 0.083496889 | gap | 0.040751669 | - | - | 0 |
| 711 | 0 | gap | 0.049961366 | gap | 0.022120053 | - | - | 0 |
| 712 | 0 | gap | 0.105145496 | gap | 0.046336535 | - | - | 0 |
| 713 | 0 | gap | 0.068032745 | gap | 0.037428953 | - | - | 0 |
| 714 | 0 | gap | 0.035857138 | gap | 0.015400408 | - | - | 0 |
| 715 | 0 | gap | 0.077322091 | gap | 0.032083724 | - | - | 0 |
| 716 | 0 | gap | 0.045344237 | gap | 0.021271981 | - | - | 0 |
| 717 | 0 | gap | 0.101443721 | gap | 0.060538563 | - | - | 0 |
| 718 | 0 | gap | 0.052306174 | gap | 0.023641924 | - | - | 0 |
| 719 | 0 | gap | 0.034590205 | gap | 0.018466623 | - | - | 0 |
| 720 | 0 | gap | 0.034616555 | gap | 0.01652165 | - | - | 0 |
| 721 | 0 | gap | 0.093504324 | gap | 0.040320981 | - | - | 0 |
| 722 | 0 | gap | 0.043561982 | gap | 0.017342197 | - | - | 0 |
| 723 | 0 | gap | 0.061507987 | gap | 0.034985936 | - | - | 0 |
| 724 | 0 | gap | 0.066598227 | gap | 0.036640519 | - | - | 0 |
| 725 | 0 | gap | 0.056633827 | gap | 0.02839195 | - | - | 0 |
| 726 | 0 | gap | 0.055910514 | gap | 0.031313728 | - | - | 0 |
| 727 | 0 | gap | 0.048743541 | gap | 0.022032494 | - | - | 0 |
| 728 | 0 | gap | 0.091597157 | gap | 0.047060247 | - | - | 0 |
| 729 | 0 | gap | 0.089369266 | gap | 0.047569014 | - | - | 0 |
| 730 | 0 | gap | 0.10317636 | gap | 0.060672978 | - | - | 0 |
| 731 | 0 | gap | 0.039274566 | gap | 0.018906993 | - | - | 0 |
| 732 | 0 | gap | 0.058636928 | gap | 0.035220851 | - | - | 0 |
| 733 | 0 | gap | 0.049883346 | gap | 0.025807514 | - | - | 0 |
| 734 | 0 | gap | 0.05296075 | gap | 0.024093523 | - | - | 0 |
| 735 | 0 | gap | 0.037045845 | gap | 0.022204422 | - | - | 0 |
| 736 | 0 | gap | 0.014603671 | gap | 0.005266279 | - | - | 0 |
| 737 | 0 | gap | 0.045742279 | gap | 0.021543077 | - | - | 0 |
| 738 | 0 | gap | 0.030264718 | gap | 0.01686518 | - | - | 0 |
| 739 | 0 | gap | 0.041320404 | gap | 0.022793696 | - | - | 0 |
| 740 | 0 | gap | 0.043401683 | gap | 0.019709604 | - | - | 0 |
| 741 | 0 | gap | 0.064496621 | gap | 0.037263755 | - | - | 0 |
| 742 | 0 | gap | 0.04625404 | gap | 0.017457878 | - | - | 0 |
| 743 | 0 | gap | 0.05765 | gap | 0.025475587 | - | - | 0 |
| 744 | 0 | gap | 0.046278744 | gap | 0.022817209 | - | - | 0 |
| 745 | 0 | gap | 0.048088376 | gap | 0.02229815 | - | - | 0 |
| 746 | 0 | gap | 0.095634449 | gap | 0.040391289 | - | - | 0 |
| 747 | 0 | gap | 0.041022311 | gap | 0.019402586 | - | - | 0 |
| 748 | 0 | gap | 0.03574987 | gap | 0.018296029 | - | - | 0 |
| 749 | 0 | gap | 0.05529861 | gap | 0.021138717 | - | - | 0 |
| 750 | 0 | gap | 0.077651466 | gap | 0.038351661 | - | - | 0 |
| 751 | 0 | gap | 0.034765811 | gap | 0.01484309 | - | - | 0 |
| 752 | 0 | gap | 0.068869566 | gap | 0.04205972 | - | - | 0 |
| 753 | 0 | gap | 0.07917785 | gap | 0.041106933 | - | - | 0 |
| 754 | 0 | gap | 0.049943133 | gap | 0.019472448 | - | - | 0 |
| 755 | 0 | gap | 0.097913868 | gap | 0.041479498 | - | - | 0 |
| 756 | 0 | gap | 0.067256189 | gap | 0.035522859 | - | - | 0 |
| 757 | 0 | gap | 0.043533611 | gap | 0.02836154 | - | - | 0 |
| 758 | 0 | gap | 0.032187202 | gap | 0.014361613 | - | - | 0 |
| 759 | 0 | gap | 0.096615004 | gap | 0.040886099 | - | - | 0 |
| 760 | 0 | gap | 0.080179403 | gap | 0.033109862 | - | - | 0 |
| 761 | 0 | gap | 0.04074901 | gap | 0.028037322 | - | - | 0 |
| 762 | 0 | gap | 0.066366149 | gap | 0.031393791 | - | - | 0 |
| 763 | 0 | gap | 0.086210597 | gap | 0.040751172 | - | - | 0 |
| 764 | 0 | gap | 0.042580319 | gap | 0.020733514 | - | - | 0 |
| 765 | 0 | gap | 0.034689485 | gap | 0.013574902 | - | - | 0 |
| 766 | 0 | gap | 0.045666006 | gap | 0.017129827 | - | - | 0 |
| 767 | 0 | gap | 0.043093315 | gap | 0.019461339 | - | - | 0 |
| 768 | 0 | gap | 0.047849158 | gap | 0.021925374 | - | - | 0 |
| 769 | 0 | gap | 0.036907828 | gap | 0.011468974 | - | - | 0 |
| 770 | 0 | gap | 0.058111794 | gap | 0.031281735 | - | - | 0 |
| 771 | 0 | gap | 0.041144581 | gap | 0.024549158 | - | - | 0 |
| 772 | 0 | gap | 0.053781703 | gap | 0.024483275 | - | - | 0 |
| 773 | 0 | gap | 0.080166719 | gap | 0.040798467 | - | - | 0 |
| 774 | 0 | gap | 0.052914339 | gap | 0.024100206 | - | - | 0 |
| 775 | 0 | gap | 0.077326344 | gap | 0.027989135 | - | - | 0 |
| 776 | 0 | gap | 0.089269395 | gap | 0.042720354 | - | - | 0 |
| 777 | 0 | gap | 0.15342399 | gap | 0.091570289 | - | - | 0 |
| 778 | 0 | gap | 0.201207566 | gap | 0.121801633 | - | - | 0 |
| 779 | 0 | gap | 0.437394797 | gap | 0.184852585 | - | - | 0 |
| 780 | 0 | gap | 0.032150782 | gap | 0.01630457 | - | - | 0 |
| 781 | 0 | gap | 0.04036451 | gap | 0.017834161 | - | - | 0 |
| 782 | 0 | gap | 0.092193431 | gap | 0.053818764 | - | - | 0 |
| 783 | 0.304145178 | S | 0.32532261 | S | 0.342282321 | N | 454 | 0.104103517 |
| 784 | 0.232417722 | P | 0.5047579 | P | 0.477480356 | R | 455 | 0.110974897 |
| 785 | 0.32961729 | W | 0.417576157 | A | 0.521029957 | T | 456 | 0.171740482 |
| 786 | 0.363328624 | gap | 0.456157267 | A | 0.499231543 | T | 457 | 0.181385109 |
| 787 | 0.419363432 | gap | 0.894328398 | G | 0.953727284 | H | 458 | 0.399958347 |
| 788 | 0.34422789 | gap | 0.511295403 | I | 0.554415789 | W | 459 | 0.190845377 |
| 789 | 0.324479621 | gap | 0.890966038 | V | 0.37884081 | V | 460 | 0.122926123 |
| 790 | 0.398331441 | gap | 0.824841509 | A | 0.629086963 | G | 461 | 0.250585117 |
| 791 | 0.534042736 | P | 1.265100502 | I | 1.099287475 | P | 462 | 0.587066491 |
| 792 | 0.613952136 | L | 0.961779998 | V | 0.556823688 | L | 463 | 0.341863092 |
| 793 | 0.782960094 | V | 0.756591726 | F | 0.577545511 | F | 464 | 0.452195088 |
| 794 | 0.685888242 | G | 0.992743962 | I | 0.907769309 | G | 465 | 0.622628295 |
| 795 | 0.493593425 | L | 0.719011699 | C | 1.175514379 | A | 466 | 0.580226169 |
| 796 | 0.612069461 | A | 0.994466145 | L | 0.451647405 | A | 467 | 0.276439584 |
| 797 | 0.716583527 | L | 1.618729632 | F | 1.519707146 | T | 468 | 1.088997107 |
| 798 | 0.876719199 | L | 1.719376801 | I | 0.997450243 | T | 469 | 0.874483778 |
| 799 | 0.542035849 | I | 1.004221146 | A | 0.693621168 | A | 470 | 0.375967539 |
| 800 | 0.699701749 | A | 1.059172374 | F | 0.371978616 | S | 471 | 0.260274088 |
| 801 | 0.935528188 | G | 2.120278088 | F | 3.470498767 | G | 472 | 3.246749424 |
| 802 | 0.591759075 | G | 1.17457471 | A | 0.737194807 | A | 473 | 0.436241717 |
| 803 | 0.634535569 | F | 0.438573499 | W | 0.649196367 | F | 474 | 0.411938186 |
| 804 | 0.415559761 | A | 1.63395469 | S | 1.829915467 | L | 475 | 0.760439234 |
| 805 | 0.616735873 | I | 1.000465908 | W | 1.733104663 | I | 476 | 1.068867817 |
| 806 | 0.53578116 | L | 2.135534435 | G | 2.804122009 | F | 477 | 1.502395743 |
| 807 | 0.51676615 | P | 1.906461062 | P | 1.514223605 | Q | 478 | 0.782499503 |
| 808 | 0.873961685 | A | 1.257219844 | V | 1.97991005 | T | 479 | 1.730365524 |
| 809 | 0.498177198 | A | 0.842326547 | P | 0.660019583 | L | 480 | 0.328806706 |
| 810 | 0.606347399 | L | 2.139942131 | W | 3.18964172 | F | 481 | 1.934030962 |
| 811 | 0.628819831 | A | 1.175304983 | V | 1.476247295 | N | 482 | 0.928293575 |
| 812 | 0.578280125 | L | 1.150146941 | I | 0.963885841 | F | 483 | 0.557396025 |
| 813 | 0.701834713 | L | 0.683383748 | L | 0.892051219 | M | 484 | 0.626072511 |
| 814 | 0.859051346 | A | 1.56353826 | S | 1.86283835 | G | 485 | 1.600273793 |
| 815 | 0.49794648 | D | 2.730204355 | E | 3.011280681 | A | 486 | 1.499456615 |
| 816 | 0.430407048 | L | 1.226048818 | I | 1.911970745 | S | 487 | 0.822925685 |
| 817 | 0.385250551 | Y | 1.9364938 | F | 2.133312143 | F | 488 | 0.821859678 |
| 818 | 0.363341622 | A | 1.893611817 | P | 2.260033915 | K | 489 | 0.821164388 |
| 819 | 0.64170181 | P | 1.030994011 | L | 1.526269113 | P | 490 | 0.979409653 |
| 820 | 0.515804411 | D | 0.632974981 | R | 0.680655826 | H | 491 | 0.351085277 |
| 821 | 0.35304348 | R | 0.583752756 | I | 1.09311718 | Y | 492 | 0.385917894 |
| 822 | 0.498267926 | A | 2.34298043 | R | 3.002453442 | I | 493 | 1.496026251 |
| 823 | 1.366114499 | G | 1.18208505 | S | 0.529094058 | A | 494 | 0.722803064 |
| 824 | 0.626772655 | S | 0.900376887 | A | 1.040808169 | S | 495 | 0.652350099 |
| 825 | 0.920423533 | V | 1.678261158 | A | 2.148086276 | V | 496 | 1.977149158 |
| 826 | 0.526251401 | F | 0.541122245 | I | 0.309321269 | F | 497 | 0.162780751 |
| 827 | 1.712581539 | A | 1.338451973 | S | 0.443322565 | A | 498 | 0.759226041 |
| 828 | 0.562565425 | I | 1.243871804 | I | 0.873741349 | S | 499 | 0.491536674 |
| 829 | 0.714493869 | N | 1.484869739 | A | 1.871956282 | N | 500 | 1.337501287 |
| 830 | 0.641787228 | G | 0.872615251 | V | 0.612724433 | D | 501 | 0.393238715 |
| 831 | 0.637758278 | L | 0.747931735 | A | 0.278738883 | L | 502 | 0.17776803 |
| 832 | 0.749885944 | F | 0.69948035 | A | 0.594288118 | F | 503 | 0.445648306 |
| 833 | 0.563100613 | R | 1.719931547 | N | 1.848815423 | R | 504 | 1.041069098 |
| 834 | 0.457446338 | S | 2.117157914 | W | 4.473510896 | S | 505 | 2.046391179 |
| 835 | 0.81474297 | L | 1.026137091 | L | 0.238212672 | V | 506 | 0.1940821 |
| 836 | 0.957402366 | G | 0.763111411 | A | 0.94354717 | I | 507 | 0.903354294 |
| 837 | 0.638319945 | A | 1.931232991 | N | 1.505602992 | A | 508 | 0.961056419 |
| 838 | 0.827808085 | A | 1.041039159 | F | 1.495199987 | S | 509 | 1.237738638 |
| 839 | 0.86913024 | I | 1.11427305 | I | 0.307270041 | V | 510 | 0.267057684 |
| 840 | 1.198581657 | G | 1.902518024 | I | 3.635057814 | F | 511 | 4.356913617 |
| 841 | 1.129782593 | P | 1.295275143 | S | 1.843953694 | P | 512 | 2.083266785 |
| 842 | 0.737488759 | L | 0.772725784 | L | 1.173974848 | L | 513 | 0.865793254 |
| 843 | 0.827487878 | L | 0.715669462 | F | 0.668763389 | F | 514 | 0.553393598 |
| 844 | 1.00829804 | G | 1.725741069 | F | 2.709437773 | G | 515 | 2.731920796 |
| 845 | 0.750411084 | G | 1.376305452 | P | 2.458606515 | A | 516 | 1.84496558 |
| 846 | 0.532966631 | A | 0.556420248 | S | 0.831745558 | P | 517 | 0.443292628 |
| 847 | 0.981369728 | L | 1.261842423 | I | 0.278445811 | L | 518 | 0.27325829 |
| 848 | 0.592587448 | F | 0.554100318 | L | 1.430566593 | F | 519 | 0.847735807 |
| 849 | 0.366754911 | D | 0.590179414 | D | 0.795345752 | D | 520 | 0.29169696 |
| 850 | 0.487289112 | N | 0.515832746 | A | 0.657876649 | N | 521 | 0.320576128 |
| 851 | 0 | gap | 0.259906321 | gap | 0.131516347 | - | - | 0 |
| 852 | 0 | gap | 0.070800448 | gap | 0.034599842 | - | - | 0 |
| 853 | 0 | gap | 0.193468175 | gap | 0.077460238 | - | - | 0 |
| 854 | 0 | gap | 0.095622086 | gap | 0.051211577 | - | - | 0 |
| 855 | 0 | gap | 0.103224374 | gap | 0.049443594 | - | - | 0 |
| 856 | 0 | gap | 0.122653507 | gap | 0.07469699 | - | - | 0 |
| 857 | 0 | gap | 0.15536142 | gap | 0.079062038 | - | - | 0 |
| 858 | 0 | gap | 0.03223759 | gap | 0.014651065 | - | - | 0 |
| 859 | 0 | gap | 0.024586533 | gap | 0.012047518 | - | - | 0 |
| 860 | 0 | gap | 0.03223759 | gap | 0.014651065 | - | - | 0 |
| 861 | 0 | gap | 0.026872881 | gap | 0.011585986 | - | - | 0 |
| 862 | 0.360205749 | L | 0.666924507 | G | 0.43973055 | L | 522 | 0.158393472 |
| 863 | 0.597549624 | G | 0.46455158 | G | 0.527809739 | A | 523 | 0.315392511 |
| 864 | 0.243516016 | F | 0.796612787 | G | 0.822923528 | T | 524 | 0.200395059 |
| 865 | 0.158898797 | gap | 0 | gap | 0.070975822 | P | 525 | 0.011277973 |
| 866 | 0.051797277 | gap | 0 | gap | 0.019631223 | E | 526 | 0.001016844 |
| 867 | 0.024679098 | gap | 0 | gap | 0.008121918 | - | - | 0.000200442 |
| 868 | 0.028338411 | gap | 0 | gap | 0.010184397 | - | - | 0.00028861 |
| 869 | 0.032861548 | gap | 0 | gap | 0.010005107 | - | - | 0.000328783 |
| 870 | 0.04229778 | gap | 0 | gap | 0.013625591 | - | - | 0.000576332 |
| 871 | 0.040024736 | gap | 0 | gap | 0.016019169 | - | - | 0.000641163 |
| 872 | 0.02703226 | gap | 0 | gap | 0.009680363 | - | - | 0.000261682 |
| 873 | 0.032861548 | gap | 0 | gap | 0.010005107 | - | - | 0.000328783 |
| 874 | 0.025907421 | gap | 0 | gap | 0.01014618 | - | - | 0.000262861 |
| 875 | 0.02593597 | gap | 0 | gap | 0.00810454 | - | - | 0.000210199 |
| 876 | 0.025907421 | gap | 0 | gap | 0.01014618 | - | - | 0.000262861 |
| 877 | 0.025907421 | gap | 0 | gap | 0.01014618 | - | - | 0.000262861 |
| 878 | 0.028707297 | gap | 0 | gap | 0.008940817 | - | - | 0.000256667 |
| 879 | 0.040024736 | gap | 0 | gap | 0.016019169 | - | - | 0.000641163 |
| 880 | 0.032861548 | gap | 0 | gap | 0.010005107 | - | - | 0.000328783 |
| 881 | 0.023660436 | gap | 0 | gap | 0.009717666 | - | - | 0.000229924 |
| 882 | 0.086688423 | gap | 0 | gap | 0.03525563 | Y | 527 | 0.003056255 |
| 883 | 0.357987381 | P | 0.63334505 | G | 0.864458143 | P | 528 | 0.309465107 |
| 884 | 0.59580801 | V | 0.841984262 | T | 0.92886735 | V | 529 | 0.553426608 |
| 885 | 0.527936035 | G | 2.11538608 | F | 2.482037622 | A | 530 | 1.3103571 |
| 886 | 0.681898139 | W | 0.874581933 | F | 0.60376346 | W | 531 | 0.41170518 |
| 887 | 0.542244732 | G | 1.115894385 | I | 0.792287724 | G | 532 | 0.429613845 |
| 888 | 0.592392115 | L | 1.651035843 | F | 1.850251905 | S | 533 | 1.096074639 |
| 889 | 0.522528382 | G | 1.468926021 | A | 0.820403397 | S | 534 | 0.428684059 |
| 890 | 0.82073147 | A | 0.930989231 | G | 0.457777085 | V | 535 | 0.37571206 |
| 891 | 0.770169009 | L | 0.815341535 | L | 0.532416352 | L | 536 | 0.410050574 |
| 892 | 0.545064284 | G | 0.678816902 | C | 0.372895573 | G | 537 | 0.203252058 |
| 893 | 0.785587902 | I | 1.1663647 | V | 0.536846239 | F | 538 | 0.421739911 |
| 894 | 1.009674045 | L | 1.155087967 | L | 0.175583849 | I | 539 | 0.177282455 |
| 895 | 0.593166874 | Y | 1.030289845 | S | 0.999247375 | T | 540 | 0.592720442 |
| 896 | 0.844558447 | A | 0.745759349 | V | 0.420367069 | L | 541 | 0.355024558 |
| 897 | 0.817648105 | L | 0.997897806 | I | 0.611568168 | V | 542 | 0.500047554 |
| 898 | 0.87509009 | L | 1.414403668 | F | 1.242766136 | M | 543 | 1.08753233 |
| 899 | 0.59055632 | L | 1.074520772 | V | 0.9402507 | I | 544 | 0.555270993 |
| 900 | 0.48298553 | P | 0.82124518 | Y | 0.92019179 | A | 545 | 0.444439319 |
| 901 | 0.786327822 | I | 0.930771672 | F | 0.541815322 | I | 546 | 0.426044462 |
| 902 | 0.767158847 | L | 0.998275739 | F | 1.030858125 | P | 547 | 0.79083193 |
| 903 | 0.40996941 | R | 1.3617471 | V | 1.368189077 | V | 548 | 0.560915669 |
| 904 | 0.290308915 | L | 1.595756921 | P | 1.626242049 | L | 549 | 0.472112564 |
| 905 | 0.497950766 | L | 2.808447995 | E | 3.998761816 | F | 550 | 1.991186508 |
| 906 | 0.384261675 | R | 2.575850031 | T | 2.638898665 | Y | 551 | 1.014027621 |
| 907 | 0.450363208 | A | 1.887526291 | K | 1.302161864 | L | 552 | 0.586445794 |
| 908 | 0.363080788 | K | 2.082202076 | G | 3.762320169 | N | 553 | 1.366026172 |
| 909 | 0.444541062 | G | 0.960351977 | R | 0.964477842 | G | 554 | 0.428750004 |
| 910 | 0.435694725 | A | 1.174887675 | T | 1.184897621 | P | 555 | 0.516253644 |
| 911 | 0.550646545 | K | 1.793654027 | L | 3.355795266 | K | 556 | 1.847857069 |
| 912 | 0.288445206 | gap | 1.776469343 | E | 1.766676084 | L | 557 | 0.509589247 |
| 913 | 0.179702626 | gap | 0 | gap | 0.08930927 | - | - | 0.01604911 |
| 914 | 0.132950631 | gap | 0 | gap | 0.064430133 | - | - | 0.008566027 |
| 915 | 0.094841092 | gap | 0 | gap | 0.026566834 | - | - | 0.002519628 |
| 916 | 0.037057918 | gap | 0 | gap | 0.011724482 | - | - | 0.000434485 |
| 917 | 0.030142224 | gap | 0 | gap | 0.010441456 | - | - | 0.000314729 |
| 918 | 0.037375494 | gap | 0 | gap | 0.011382084 | - | - | 0.000425411 |
| 919 | 0.035342094 | gap | 0 | gap | 0.014257348 | - | - | 0.000503885 |
| 920 | 0.045113278 | gap | 0 | gap | 0.017181158 | - | - | 0.000775098 |
| 921 | 0.045716234 | gap | 0 | gap | 0.019431313 | - | - | 0.000888326 |
| 922 | 0.04898511 | gap | 0 | gap | 0.018755584 | - | - | 0.000918744 |
| 923 | 0.04898511 | gap | 0 | gap | 0.018755584 | - | - | 0.000918744 |
| 924 | 0.034555497 | gap | 0 | gap | 0.013466401 | - | - | 0.000465338 |
| 925 | 0.118358642 | gap | 0 | gap | 0.042658743 | - | - | 0.005049031 |
| 926 | 0.036957813 | gap | 0 | gap | 0.009780374 | - | - | 0.000361461 |
| 927 | 0.068200798 | gap | 0 | gap | 0.027091241 | - | - | 0.001847644 |
| 928 | 0.105167768 | gap | 0 | gap | 0.046960773 | - | - | 0.00493876 |
| 929 | 0.068170042 | gap | 0 | gap | 0.031373932 | - | - | 0.002138762 |
| 930 | 0.072107041 | gap | 0 | gap | 0.032099562 | - | - | 0.002314604 |
| 931 | 0.075659158 | gap | 0 | gap | 0.0347012 | - | - | 0.002625464 |
| 932 | 0.081128861 | gap | 0 | gap | 0.031794919 | - | - | 0.002579486 |
| 933 | 0.096624707 | gap | 0 | gap | 0.033755662 | - | - | 0.003261631 |
| 934 | 0.081735006 | gap | 0 | gap | 0.028038496 | - | - | 0.002291727 |
| 935 | 0.13438667 | gap | 0 | gap | 0.058217984 | - | - | 0.007823721 |
| 936 | 0.063255472 | gap | 0 | gap | 0.034089772 | - | - | 0.002156365 |
| 937 | 0.106775099 | gap | 0 | gap | 0.039711427 | - | - | 0.004240192 |
| 938 | 0.434797367 | gap | 1.818302298 | E | 1.484759049 | R | 558 | 0.645569326 |
| 939 | 0.419578075 | gap | 1.5225927 | I | 2.235646677 | A | 559 | 0.93802833 |
| 940 | 0.370597246 | gap | 0.729022376 | D | 0.679893093 | R | 560 | 0.251966508 |
| 941 | 0.438031303 | gap | 0.554039025 | E | 0.429956343 | S | 561 | 0.188334337 |
| 942 | 0.275357365 | gap | 0.467814161 | L | 0.833479474 | K | 562 | 0.229504712 |
| 943 | 0.25876455 | gap | 0.797541113 | F | 0.950368787 | Y | 563 | 0.245921751 |
| 944 | 0.338959915 | gap | 0.417173562 | E | 0.696400025 | A | 564 | 0.236051693 |
| 945 | 0.240086128 | gap | 0.530592353 | K | 0.452788446 | N | 565 | 0.108708225 |
| 946 | 0.136237168 | gap | 0.573648681 | G | 0.571599192 | - | - | 0.077873055 |
| 947 | 0.201010035 | gap | 0.291834172 | gap | 0.550548552 | - | - | 0.110665784 |
| 948 | 0.261472887 | gap | 0.268654315 | K | 0.78894953 | - | - | 0.206288911 |
| 949 | 0.160774946 | gap | 0.256873149 | gap | 0.617131014 | - | - | 0.099219206 |
| 950 | 0.214152773 | gap | 0.342280388 | W | 0.96342824 | - | - | 0.206320829 |
| 951 | 0.066318854 | gap | 0.390912843 | K | 0.585620676 | - | - | 0.038837692 |
| 952 | 0.106440908 | gap | 0.545184539 | S | 0.341261159 | - | - | 0.036324147 |
| 953 | 0.134671637 | gap | 0.342503113 | gap | 0.424767486 | - | - | 0.057204133 |
| 954 | 0.053635023 | gap | 0.290100471 | gap | 0.55838479 | - | - | 0.029948981 |
| 955 | 0.286713438 | gap | 0.245645346 | gap | 0.907854657 | - | - | 0.26029413 |
| 956 | 0.088542249 | gap | 0.358598344 | gap | 0.210863171 | - | - | 0.018670299 |
| 957 | 0.172523143 | gap | 0.165538405 | gap | 0.227708541 | - | - | 0.039284993 |
| 958 | 0.03280832 | gap | 0.228497004 | gap | 0.206133925 | - | - | 0.006762908 |
| 959 | 0.092632468 | gap | 0.106319626 | gap | 0.224334039 | - | - | 0.020780616 |
| 960 | 0.06467687 | gap | 0.158633124 | gap | 0.180616487 | - | - | 0.011681709 |
| 961 | 0.035011169 | gap | 0.076663833 | gap | 0.142101468 | - | - | 0.004975138 |
| 962 | 0.042935357 | gap | 0.193935512 | gap | 0.273604403 | - | - | 0.011747303 |
| 963 | 0.084021813 | gap | 0.086356887 | gap | 0.198741592 | - | - | 0.016698629 |
| 964 | 0.039180128 | gap | 0.080324574 | gap | 0.124657508 | - | - | 0.004884097 |
| 965 | 0.037218022 | gap | 0.051818424 | gap | 0.078801155 | - | - | 0.002932823 |
| 966 | 0 | gap | 0.05861221 | gap | 0.030635695 | - | - | 0 |
| 967 | 0 | gap | 0.060413018 | gap | 0.030459952 | - | - | 0 |
| 968 | 0 | gap | 0.067515505 | gap | 0.025120118 | - | - | 0 |
| 969 | 0 | gap | 0.040977988 | gap | 0.013448049 | - | - | 0 |
| 970 | 0 | gap | 0.031575632 | gap | 0.016166861 | - | - | 0 |
| 971 | 0 | gap | 0.031575632 | gap | 0.016166861 | - | - | 0 |
| 972 | 0 | gap | 0.027240277 | gap | 0.010537588 | - | - | 0 |
| 973 | 0 | gap | 0.031720044 | gap | 0.014942485 | - | - | 0 |
| 974 | 0 | gap | 0.021472103 | gap | 0.011195326 | - | - | 0 |
| 975 | 0 | gap | 0.046928665 | gap | 0.022596887 | - | - | 0 |
| 976 | 0 | gap | 0.028467658 | gap | 0.012486974 | - | - | 0 |
| 977 | 0 | gap | 0.031285184 | gap | 0.015496679 | - | - | 0 |
| 978 | 0 | gap | 0.030464447 | gap | 0.011061246 | - | - | 0 |
| 979 | 0 | gap | 0.07035579 | gap | 0.031203957 | - | - | 0 |
| 980 | 0 | gap | 0.038440862 | gap | 0.01735874 | - | - | 0 |
| 981 | 0 | gap | 0.037442542 | gap | 0.013641091 | - | - | 0 |
| 982 | 0 | gap | 0.039006913 | gap | 0.016309772 | - | - | 0 |
| 983 | 0 | gap | 0.035030551 | gap | 0.014370917 | - | - | 0 |
| 984 | 0 | gap | 0.0683443 | gap | 0.026722885 | - | - | 0 |
| 985 | 0 | gap | 0.066110382 | gap | 0.035454676 | - | - | 0 |
| 986 | 0 | gap | 0.03973175 | gap | 0.023270294 | - | - | 0 |
| 987 | 0 | gap | 0.042588341 | gap | 0.017753744 | - | - | 0 |
| 988 | 0 | gap | 0.036478258 | gap | 0.020007436 | - | - | 0 |
| 989 | 0 | gap | 0.04968869 | gap | 0.020499585 | - | - | 0 |
| 990 | 0 | gap | 0.050540465 | gap | 0.026398795 | - | - | 0 |
| 991 | 0 | gap | 0.048750524 | gap | 0.023675073 | - | - | 0 |
| 992 | 0 | gap | 0.123967548 | gap | 0.049668491 | - | - | 0 |
| 993 | 0 | gap | 0.050053336 | gap | 0.026625158 | - | - | 0 |
| 994 | 0 | gap | 0.047385406 | gap | 0.020116543 | - | - | 0 |
| 995 | 0 | gap | 0.081334997 | gap | 0.040796483 | - | - | 0 |
| 996 | 0 | gap | 0.03689845 | gap | 0.021582926 | - | - | 0 |
| 997 | 0 | gap | 0.033726055 | gap | 0.016501 | - | - | 0 |
| 998 | 0 | gap | 0.046472951 | gap | 0.023881579 | - | - | 0 |
| 999 | 0 | gap | 0.036888871 | gap | 0.013810706 | - | - | 0 |
| 1000 | 0 | gap | 0.04846599 | gap | 0.018365992 | - | - | 0 |
| 1001 | 0 | gap | 0.074572899 | gap | 0.037592406 | - | - | 0 |
| 1002 | 0 | gap | 0.201053629 | gap | 0.105738279 | - | - | 0 |
| 1003 | 0 | gap | 0.173953511 | gap | 0.086593917 | - | - | 0 |
| 1004 | 0 | gap | 0.267413801 | gap | 0.136465304 | - | - | 0 |
| 1005 | 0 | gap | 0.252044895 | gap | 0.113250158 | - | - | 0 |
| 1006 | 0 | gap | 0.182483618 | gap | 0.085210261 | - | - | 0 |
| 1007 | 0 | gap | 0.233608027 | gap | 0.123397668 | - | - | 0 |
| 1008 | 0 | gap | 0.271362897 | gap | 0.155276169 | - | - | 0 |
| 1009 | 0 | gap | 0.204123103 | gap | 0.13309918 | - | - | 0 |
| 1010 | 0 | gap | 0.186190713 | gap | 0.093918977 | - | - | 0 |
| 1011 | 0 | gap | 0.372627708 | gap | 0.192180002 | - | - | 0 |
| 1012 | 0 | gap | 0.212732838 | gap | 0.073017778 | - | - | 0 |
| 1013 | 0 | gap | 0.211250035 | gap | 0.06972127 | - | - | 0 |
| 1014 | 0 | gap | 0.22600332 | gap | 0.132468656 | - | - | 0 |
| 1015 | 0 | gap | 0.191770926 | gap | 0.11485936 | - | - | 0 |
| 1016 | 0 | gap | 0.186328595 | gap | 0.126155457 | - | - | 0 |
| 1017 | 0 | gap | 0.211040935 | gap | 0.102335839 | - | - | 0 |
| 1018 | 0 | gap | 0.209698661 | gap | 0.093987917 | - | - | 0 |
| 1019 | 0 | gap | 0.232294589 | gap | 0.09479528 | - | - | 0 |
| 1020 | 0 | gap | 0.15798769 | gap | 0.090930352 | - | - | 0 |
| 1021 | 0 | gap | 0.189861492 | gap | 0.09141801 | - | - | 0 |
| 1022 | 0 | gap | 0.109576942 | gap | 0.054521543 | - | - | 0 |
| 1023 | 0 | gap | 0.217371748 | gap | 0.111680829 | - | - | 0 |
| 1024 | 0 | gap | 0.153031331 | gap | 0.075579084 | - | - | 0 |
| 1025 | 0 | gap | 0.191563058 | gap | 0.098800563 | - | - | 0 |
| 1026 | 0 | gap | 0.139698872 | gap | 0.064039315 | - | - | 0 |
| 1027 | 0 | gap | 0.146263814 | gap | 0.069534341 | - | - | 0 |
| 1028 | 0 | gap | 0.154206815 | gap | 0.072890766 | - | - | 0 |
| 1029 | 0 | gap | 0.098484576 | gap | 0.053598302 | - | - | 0 |
| 1030 | 0 | gap | 0.148597637 | gap | 0.061765867 | - | - | 0 |
| 1031 | 0 | gap | 0.028293291 | gap | 0.011212652 | - | - | 0 |
| 1032 | 0 | gap | 0.019935254 | gap | 0.007757144 | - | - | 0 |
| 1033 | 0 | gap | 0.022715957 | gap | 0.007438993 | - | - | 0 |
| 1034 | 0 | gap | 0.032563685 | gap | 0.015441579 | - | - | 0 |
| 1035 | 0 | gap | 0.026929943 | gap | 0.009692275 | - | - | 0 |
| 1036 | 0 | gap | 0.019935254 | gap | 0.007757144 | - | - | 0 |
| 1037 | 0 | gap | 0.021062005 | gap | 0.007780251 | - | - | 0 |
| 1038 | 0 | gap | 0.019935254 | gap | 0.007757144 | - | - | 0 |
| 1039 | 0 | gap | 0.023025526 | gap | 0.009817294 | - | - | 0 |
| 1040 | 0 | gap | 0.025140542 | gap | 0.012663282 | - | - | 0 |
| 1041 | 0 | gap | 0.021981981 | gap | 0.009340253 | - | - | 0 |
| 1042 | 0 | gap | 0.032563685 | gap | 0.015441579 | - | - | 0 |
| 1043 | 0 | gap | 0.023530601 | gap | 0.008663473 | - | - | 0 |
| 1044 | 0 | gap | 0.023025526 | gap | 0.009817294 | - | - | 0 |
| 1045 | 0 | gap | 0.022594666 | gap | 0.012822612 | - | - | 0 |
| 1046 | 0 | gap | 0.032563685 | gap | 0.015441579 | - | - | 0 |
| 1047 | 0 | gap | 0.032563685 | gap | 0.015441579 | - | - | 0 |
| 1048 | 0 | gap | 0.023530601 | gap | 0.008663473 | - | - | 0 |
| 1049 | 0 | gap | 0.02094098 | gap | 0.009736136 | - | - | 0 |
| 1050 | 0 | gap | 0.02584924 | gap | 0.013788418 | - | - | 0 |
| 1051 | 0 | gap | 0.023530601 | gap | 0.008663473 | - | - | 0 |
| 1052 | 0 | gap | 0.026864093 | gap | 0.012018041 | - | - | 0 |
| 1053 | 0 | gap | 0.026929943 | gap | 0.009692275 | - | - | 0 |
| 1054 | 0 | gap | 0.034541673 | gap | 0.013144751 | - | - | 0 |
| 1055 | 0 | gap | 0.02094098 | gap | 0.009736136 | - | - | 0 |
| 1056 | 0 | gap | 0.034541673 | gap | 0.013144751 | - | - | 0 |
| 1057 | 0 | gap | 0.026929943 | gap | 0.009692275 | - | - | 0 |
| 1058 | 0 | gap | 0.034541673 | gap | 0.013144751 | - | - | 0 |
| 1059 | 0 | gap | 0.026929943 | gap | 0.009692275 | - | - | 0 |
| 1060 | 0 | gap | 0.02094098 | gap | 0.009736136 | - | - | 0 |
| 1061 | 0 | gap | 0.019935254 | gap | 0.007757144 | - | - | 0 |
| 1062 | 0 | gap | 0.02584924 | gap | 0.013788418 | - | - | 0 |
| 1063 | 0 | gap | 0.02094098 | gap | 0.009736136 | - | - | 0 |
| 1064 | 0 | gap | 0.025206159 | gap | 0.011151039 | - | - | 0 |
| 1065 | 0 | gap | 0.034541673 | gap | 0.013144751 | - | - | 0 |
| 1066 | 0 | gap | 0.032563685 | gap | 0.015441579 | - | - | 0 |
| 1067 | 0 | gap | 0.032563685 | gap | 0.015441579 | - | - | 0 |
| 1068 | 0 | gap | 0.021062005 | gap | 0.007780251 | - | - | 0 |
| 1069 | 0 | gap | 0.023945477 | gap | 0.009744846 | - | - | 0 |
| 1070 | 0 | gap | 0.023945477 | gap | 0.009744846 | - | - | 0 |
| 1071 | 0 | gap | 0.021062005 | gap | 0.007780251 | - | - | 0 |
| 1072 | 0 | gap | 0.026864093 | gap | 0.012018041 | - | - | 0 |
| 1073 | 0 | gap | 0.021062005 | gap | 0.007780251 | - | - | 0 |
| 1074 | 0 | gap | 0.019935254 | gap | 0.007757144 | - | - | 0 |
| 1075 | 0 | gap | 0.026864093 | gap | 0.012018041 | - | - | 0 |
| 1076 | 0 | gap | 0.023025526 | gap | 0.009817294 | - | - | 0 |
| 1077 | 0 | gap | 0.025140542 | gap | 0.012663282 | - | - | 0 |
| 1078 | 0 | gap | 0.02584924 | gap | 0.013788418 | - | - | 0 |
| 1079 | 0 | gap | 0.023025526 | gap | 0.009817294 | - | - | 0 |
| 1080 | 0 | gap | 0.023025526 | gap | 0.009817294 | - | - | 0 |
| 1081 | 0 | gap | 0.029831587 | gap | 0.011016415 | - | - | 0 |
| 1082 | 0 | gap | 0.039901448 | gap | 0.018198324 | - | - | 0 |
| 1083 | 0 | gap | 0.073397468 | gap | 0.030060526 | - | - | 0 |
| 1084 | 0 | gap | 0.033193369 | gap | 0.010555823 | - | - | 0 |
| 1085 | 0 | gap | 0.042093896 | gap | 0.019550512 | - | - | 0 |
| 1086 | 0 | gap | 0.035494449 | gap | 0.020057177 | - | - | 0 |
| 1087 | 0 | gap | 0.035158494 | gap | 0.017841556 | - | - | 0 |
| 1088 | 0 | gap | 0.031153867 | gap | 0.012310247 | - | - | 0 |
| 1089 | 0 | gap | 0.031153867 | gap | 0.012310247 | - | - | 0 |
| 1090 | 0 | gap | 0.045096636 | gap | 0.018973744 | - | - | 0 |
| 1091 | 0 | gap | 0.031123676 | gap | 0.014727886 | - | - | 0 |
| 1092 | 0 | gap | 0.034900014 | gap | 0.013717169 | - | - | 0 |
| 1093 | 0 | gap | 0.034311523 | gap | 0.016959282 | - | - | 0 |
| 1094 | 0 | gap | 0.05210595 | gap | 0.019739827 | - | - | 0 |
| 1095 | 0 | gap | 0.034015246 | gap | 0.013444104 | - | - | 0 |
| 1096 | 0 | gap | 0.045573577 | gap | 0.02379079 | - | - | 0 |
| 1097 | 0 | gap | 0.051010751 | gap | 0.019792362 | - | - | 0 |
| 1098 | 0 | gap | 0.042182575 | gap | 0.020015014 | - | - | 0 |
| 1099 | 0 | gap | 0.034057679 | gap | 0.015981899 | - | - | 0 |
| 1100 | 0 | gap | 0.031561513 | gap | 0.014014829 | - | - | 0 |
| 1101 | 0 | gap | 0.036970496 | gap | 0.017640871 | - | - | 0 |
| 1102 | 0 | gap | 0.031753859 | gap | 0.014055397 | - | - | 0 |
| 1103 | 0 | gap | 0.031734298 | gap | 0.014451429 | - | - | 0 |
| 1104 | 0 | gap | 0.033249092 | gap | 0.018264045 | - | - | 0 |
| 1105 | 0 | gap | 0.034572189 | gap | 0.012669569 | - | - | 0 |
| 1106 | 0 | gap | 0.031133915 | gap | 0.012967973 | - | - | 0 |
| 1107 | 0 | gap | 0.031041944 | gap | 0.018856964 | - | - | 0 |
| 1108 | 0 | gap | 0.051010751 | gap | 0.019792362 | - | - | 0 |
| 1109 | 0 | gap | 0.038516638 | gap | 0.013868332 | - | - | 0 |
| 1110 | 0 | gap | 0.073397468 | gap | 0.030060526 | - | - | 0 |
| 1111 | 0 | gap | 0.034009797 | gap | 0.016074695 | - | - | 0 |
| 1112 | 0 | gap | 0.03169604 | gap | 0.014016789 | - | - | 0 |
| 1113 | 0 | gap | 0.035366359 | gap | 0.016238389 | - | - | 0 |
| 1114 | 0 | gap | 0.040295562 | gap | 0.020320588 | - | - | 0 |
| 1115 | 0 | gap | 0.048692564 | gap | 0.023994201 | - | - | 0 |
| 1116 | 0 | gap | 0.033413923 | gap | 0.015193969 | - | - | 0 |
| 1117 | 0 | gap | 0.051010751 | gap | 0.019792362 | - | - | 0 |
| 1118 | 0 | gap | 0.032186608 | gap | 0.015126382 | - | - | 0 |
| 1119 | 0 | gap | 0.073397468 | gap | 0.030060526 | - | - | 0 |
| 1120 | 0 | gap | 0.043522591 | gap | 0.020040717 | - | - | 0 |
| 1121 | 0 | gap | 0.073397468 | gap | 0.030060526 | - | - | 0 |
| 1122 | 0 | gap | 0.030127269 | gap | 0.015426083 | - | - | 0 |
| 1123 | 0 | gap | 0.045573577 | gap | 0.02379079 | - | - | 0 |
| 1124 | 0 | gap | 0.043482632 | gap | 0.020252845 | - | - | 0 |
| 1125 | 0 | gap | 0.032033275 | gap | 0.017603899 | - | - | 0 |
| 1126 | 0 | gap | 0.050923802 | gap | 0.01985864 | - | - | 0 |
| 1127 | 0 | gap | 0.035795363 | gap | 0.013563171 | - | - | 0 |
| 1128 | 0 | gap | 0.031561513 | gap | 0.014014829 | - | - | 0 |
| 1129 | 0 | gap | 0.037228471 | gap | 0.017152679 | - | - | 0 |
| 1130 | 0 | gap | 0.030660286 | gap | 0.014504095 | - | - | 0 |
| 1131 | 0 | gap | 0.045573577 | gap | 0.02379079 | - | - | 0 |
| 1132 | 0 | gap | 0.035135951 | gap | 0.021786949 | - | - | 0 |
| 1133 | 0 | gap | 0.040404855 | gap | 0.015899915 | - | - | 0 |
| 1134 | 0 | gap | 0.034243227 | gap | 0.014963145 | - | - | 0 |
| 1135 | 0 | gap | 0.0391539 | gap | 0.020781915 | - | - | 0 |
| 1136 | 0 | gap | 0.037956599 | gap | 0.01203361 | - | - | 0 |
| 1137 | 0 | gap | 0.077358734 | gap | 0.029165806 | - | - | 0 |
| 1138 | 0 | gap | 0.033546055 | gap | 0.012493094 | - | - | 0 |
| 1139 | 0 | gap | 0.076058678 | gap | 0.034111671 | - | - | 0 |
| 1140 | 0 | gap | 0.03119002 | gap | 0.014497086 | - | - | 0 |
| 1141 | 0 | gap | 0.051010751 | gap | 0.019792362 | - | - | 0 |
| 1142 | 0 | gap | 0.033249092 | gap | 0.018264045 | - | - | 0 |
| 1143 | 0 | gap | 0.050302613 | gap | 0.032753459 | - | - | 0 |
| 1144 | 0 | gap | 0.031561513 | gap | 0.014014829 | - | - | 0 |
| 1145 | 0 | gap | 0.043482632 | gap | 0.020252845 | - | - | 0 |
| 1146 | 0 | gap | 0.03631719 | gap | 0.017976798 | - | - | 0 |
| 1147 | 0 | gap | 0.019072427 | gap | 0.009310899 | - | - | 0 |

**Supplementary DataSet DS1: RE across DHA1 and SP families, CRE and CRE_S_ scores for the entire MSA.** RE across each of the family is calculated from their individual PRALINETM alignments as described in Materials and Methods. The dataset shows these scores for all the positions of the MSA. MFA across each family is listed for each alignment column. If the MFA matches with the CaMdr1p residue at a particular alignment position and has a high CRE_S_ score, it was mutated to alanine for further analysis. (MFA-Most frequent amino acid in the respective alignment position in the MSA).
