# Supplementary material for: Employing Information Theoretic Measures and Mutagenesis to Identify Residues Critical for Drug-Proton Antiport Function in Mdr1p of Candida albicans
Source: PLoS One. 2010 Jun 10;5(6):e11041. doi: 10.1371/journal.pone.0011041 (PMC2883579; doi:10.1371/journal.pone.0011041)
Supplement: Supplementary Data S3 — List of yeast strains used in this study. (0.05 MB DOC) [file pone.0011041.s003.doc]

|  | **Strain** | **Genotype** | **Source** |
| --- | --- | --- | --- |
|  | AD1-8u^-^ | (Mata, pdr1-3, his1, ura3, Δyor1::hisG,  Δsnq2::hisG, Δpdr5::hisG, Δpdr10::hisG,  Δpdr11::hisG, Δycf1::hisG, Δpdr3::hisG,  Δpdr15::hisG) | [23, 24] |
|  | WT-CaMDR1-GFP | AD1-8u^-^ cells harboring CaMDR1-GFP ORF integrated at PDR5 locus | [19] |
|  | KKCaMDR1-D235A | CaMDR1-GFP cells carrying D235A mutation in CaMDR1 ORF and integrated at PDR5 locus | [8] |
|  | KKCaMDR1-G264L | CaMDR1-GFP cells carrying G264L mutation in CaMDR1 ORF and integrated at PDR5 locus | This study |
|  | KKCaMDR1-P261A | CaMDR1-GFP cells carrying P261A mutation in CaMDR1 ORF and integrated at PDR5 locus | This study |
|  | KKCaMDR1-G133L | CaMDR1-GFP cells carrying G133L mutation in CaMDR1 ORF and integrated at PDR5 locus | This study |
|  | KKCaMDR1-A231G | CaMDR1-GFP cells carrying A231G mutation in CaMDR1 ORF and integrated at PDR5 locus | This study |
|  | KKCaMDR1-G472L | CaMDR1-GFP cells carrying G472L mutation in CaMDR1 ORF and integrated at PDR5 locus | This study |
|  | KKCaMDR1-V364L | CaMDR1-GFP cells carrying V364A mutation in CaMDR1 ORF and integrated at PDR5 locus | This study |
|  | KKCaMDR1-Y369A | CaMDR1-GFP cells carrying Y369A mutation in CaMDR1 ORF and integrated at PDR5 locus | This study |
|  | KKCaMDR1-G391L | CaMDR1-GFP cells carrying G391L mutation in CaMDR1 ORF and integrated at PDR5 locus | This study |
|  | KKCaMDR1-G515L | CaMDR1-GFP cells carrying G515L mutation in CaMDR1 ORF and integrated at PDR5 locus | This study |
|  | KKCaMDR1-M132A | CaMDR1-GFP cells carrying M132A mutation in CaMDR1 ORF and integrated at PDR5 locus | This study |
|  | KKCaMDR1-F277A | CaMDR1-GFP cells carrying F277A mutation in CaMDR1 ORF and integrated at PDR5 locus | [8] |
|  | KKCaMDR1-P139A | CaMDR1-GFP cells carrying P139A mutation in CaMDR1 ORF and integrated at PDR5 locus | This study |
|  | KKCaMDR1-L246A | CaMDR1-GFP cells carrying L246A mutation in CaMDR1 ORF and integrated at PDR5 locus | This study |
|  | KKCaMDR1-P512A | CaMDR1-GFP cells carrying P512A mutation in CaMDR1 ORF and integrated at PDR5 locus | This study |
|  | KKCaMDR1-S232A | CaMDR1-GFP cells carrying S232A mutation in CaMDR1 ORF and integrated at PDR5 locus | This study |
|  | KKCaMDR1-S505A | CaMDR1-GFP cells carrying S505A mutation in CaMDR1 ORF and integrated at PDR5 locus | This study |
|  | KKCaMDR1-P257A | CaMDR1-GFP cells carrying P257A mutation in CaMDR1 ORF and integrated at PDR5 locus | This study |
|  | KKCaMDR1-V496A | CaMDR1-GFP cells carrying V496A mutation in CaMDR1 ORF and integrated at PDR5 locus | This study |

**Supplementary Data S3: List of yeast strains used in this study**
