# Supplementary material for: Employing Information Theoretic Measures and Mutagenesis to Identify Residues Critical for Drug-Proton Antiport Function in Mdr1p of Candida albicans
Source: PLoS One. 2010 Jun 10;5(6):e11041. doi: 10.1371/journal.pone.0011041 (PMC2883579; doi:10.1371/journal.pone.0011041)
Supplement: Supplementary Data S4 — List of oligonucleotides used for site-directed mutagenesis. (0.06 MB DOC) [file pone.0011041.s004.doc]

| **Primer Name** | **Primer Sequence** |
| --- | --- |
| G264L/F | 5’-GGTCCATTCTTT**TTA**TCAATTTTAACTG-3’ |
| G264L/R | 5’-CAGTTAAAATTGA**TAA**AAAGAATGGACC -3’ |
| P261A/F | 5’-GTGGTCCTAGTTTTGGT**GCT**TTCTTTGGTTC-3' |
| P261A/R | 5’-GAACCAAAGAA**AGC**ACCAAAACTAGGACCAC-3' |
| G133L/F | 5’-CTTCAGTTTATATG**TTA**TCAGCAGTTTATAC-3’ |
| G133L/R | 5’-GTATAAACTGCTGA**TAA**CATATAAACTGAAG-3’ |
| A231G/F | 5’-GCTACTGGTGGT**GGA**AGTGTTGCTGATG-3’ |
| A231G/R | 5’-CATCAGCAACACT**TCC**ACCACCAGTAGC-3’ |
| G472L/F | 5’-CTACTACTGCTTCT**TTA**GCATTTTTGATTTTC-3’ |
| G472L/R | 5’-GAAAATCAAAAATGC**TAA**AGAAGCAGTAGTAG-3’ |
| V364A/F | 5’-CATTGCCATG**GCG**TACAGTATTCTTTAC-3’ |
| V364A/R | 5’-GTAAAGAATACTGTA**CGC**CATGGCAATG-3’ |
| Y369A/F | 5’-GTACAGTATTCTT**GCC**TTGTTTTTCGAAG-3’ |
| Y369A/R | 5’-CTTCGAAAAACAA**GGC**AAGAATACTGTAC-3’ |
| G391L/F | 5’-CTCGTTGAATTG**CTT**ACCACATATATG-3’ |
| G391L/R | 5’-CATATATGTGGT**AAG**CAATTCAACGAG-3’ |
| G515L/F | 5’-GTTCCCATTATTT**CTT**GCTCCTTTGTTTG-3’ |
| G515L/R | 5’-CAAACAAAGGAGC**AAG**AAATAATGGGAAC-3’ |
| M132A/F | 5’-CAACTTCAGTTTAT**GCG**GGATCAGCAGTTTATAC-3’ |
| M132A/R | 5’-GTATAAACTGCTGATCC**CGC**ATAAACTGAAGTTG-3’ |
| P139A/F | 5’-CAGCAGTTTATACC**GCT**GGTATTGAAGAATTA-3’ |
| P139A/R | 5’-TAATTCTTCAATACC**AGC**GGTATAAACTGCTG-3’ |
| L246A/F | 5’-GAATTTACCAGTTGGG**GCT**GCCGCTTGGAGTTTGGGTG-3' |
| L246A/R | 5’-CACCCAAACTCCAAGCGGC**AGC**CCCAACTGGTAAATTC-3' |
| P512A/F | 5'-GCATCAGTGTTC**GCA**TTATTTGGTGCTC-3’ |
| P512A/R | 5'-GAGCACCAAATAA**TGC**GAACACTGATGC -3’ |
| S232A/F | 5’-CTACTGGTGGTGCA**GCT**GTTGCTGATGTG-3’ |
| S232A/R | 5’-CACATCAGCAAC**AGC**TGCACCACCAGTAG-3’ |
| S505A/F | 5’-GATTTGTTCAGA**GCA**GTCATTGCATCAG-3’ |
| S505A/R | 5’-CTGATGCAATGAC**TGC**TCTGAACAAATC-3’ |
| P257A/F | 5’-GCTGTTTGTGGT**GCT**AGTTTTGGTCC-3’ |
| P257A/R | 5’-GGACCAAAACT**AGC**ACCACAAACAGC-3’ |
| V496A/F | 5’-CATTATATTGCTTCA**GCT**TTTGCATCAAATG-3’ |
| V496A/R | 5’-CATTTGATGCAAA**AGC**TGAAGCAATATAATG-3’ |

**Supplementary Data S4: List of oligonucleotides used for site-directed mutagenesis.** The respective codons are marked in bold and underlined.
